# Immunosuppressive Sesquiterpene Pyridine Alkaloids from *Tripterygium wilfordii* Hook. f.

**DOI:** 10.3390/molecules27217274

**Published:** 2022-10-26

**Authors:** Yadan Wang, Jiangong Yan, Zhongmou Zhang, Minghui Chen, Xianfu Wu, Shuangcheng Ma

**Affiliations:** 1 National Institutes for Food and Drug Control, Beijing 102629, China; 2 School of Traditional Chinese Pharmacy, China Pharmaceutical University, Nanjing 211100, China; 3 State Key Laboratory of Bioactive Substance and Function of Natural Medicines, Institute of Materia Medica, Chinese Academy of Medical Sciences and Peking Union Medical College, Beijing 100050, China

**Keywords:** sesquiterpene pyridine alkaloids, *Tripterygium wilfordii*, immunosuppressive

## Abstract

*Tripterygium wilfordii* Hook. f. is a well-known traditional Chinese medicine used to treat autoimmune diseases. Sesquiterpene pyridine alkaloids (SPAs) are a major class of components found in this herb that have piqued the interest of researchers due to their complex and diverse structures as well as significant biological activities. In this study, ten new SPAs, wilfordatine A–J (**1**–**10**), were isolated from the roots of *T. wilfordii*, along with ten known analogues (**11**–**20**). Their structures were primarily elucidated by extensive 1D and 2D NMR spectroscopic analysis. To search for more immunosuppressive ingredients related to the clinical efficacy of *T. wilfordii*, the total alkaloids (TA) and compounds **4**, **5**, and **9**–**16** were tested for their inhibitory effects on nuclear factor-kappa B (NF-κB) pathway in Lipopolysaccharide (LPS) induced HEK293/NF-κB-Luc cells. Among them, TA, compounds **5**, **11**, and **16** showed potent immunosuppressive activity, with IC_50_ values of 7.25 μg/mL, 8.75 μM, 0.74 μM, and 15.66 μM, respectively, and no influence on the cell viability at a concentration of 100 μg/mL (TA) or 100 μM (**5**, **11**, and **16**). Accordingly, TA, **5**, **11**, and **16**, especially **11**, were identified as promising candidates for further investigation into their potential use as immunosuppressive agents.

## 1. Introduction

*Tripterygium wilfordii* Hook. f., a member of the Celastraceae family, has been used medicinally in China for the treatment of rheumatoid arthritis and other autoimmune diseases for centuries [1,2]. Chemical studies on this plant resulted in the isolation of a number of sesquiterpenoid pyridine alkaloids (SPAs) with a variety of biological activities, including anti-inflammatory, insecticidal, anti-HIV, and antitumor activities [3,4,5,6,7,8,9]. SPAs are characterized by a macrocyclic diacetone skeleton composed of a polyoxygenated dihydro-β-agarofuran sesquiterpenoid core and a pyridine dicarboxylic acid moiety. According to the origin of the pyridine dicarboxylic acid, SPAs are mainly classified into four subtypes: wilfordate, evoninate, iso-wilfordate, and iso-evoninate [10]. In addition, the multiple hydroxyl groups of the SPAs are typically esterified by various organic acids, such as acetic, furoic, benzoic, and cinnamic acids. Due to their complex and diverse structures, as well as significant activities, SPAs have attracted continuous attention from researchers.

**Figure 1:**
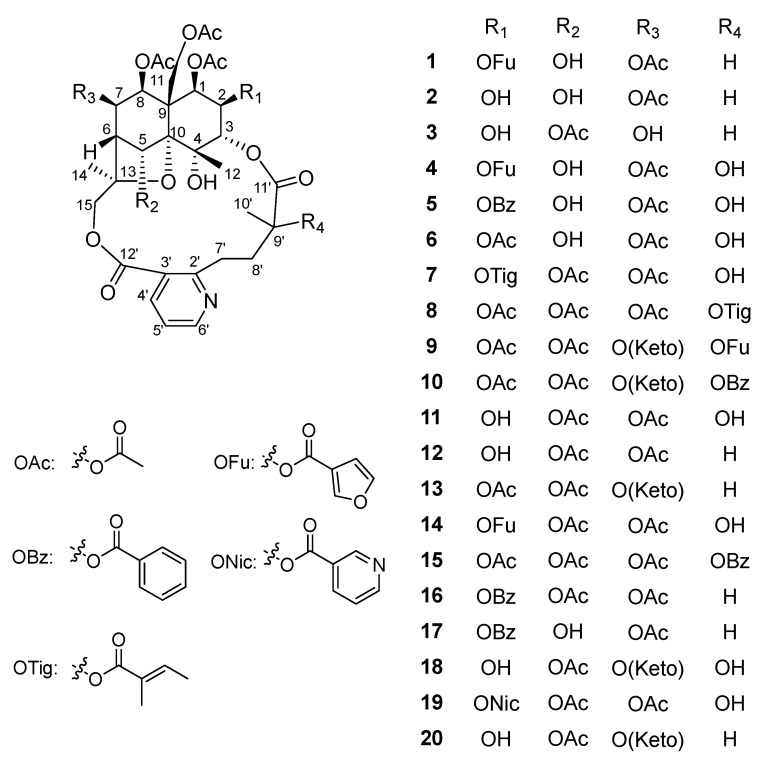
Chemical structures of the isolated SPAs (**1**–**20**).

The total alkaloids of *T. wilfordii* have been reported to possess good therapeutic effects on collagen-induced arthritis in rats through significant immunosuppressive activity. One of the molecular mechanisms is the inhibition of nuclear factor-kappa B (NF-κB) pathway [11]. To search for more immunosuppressive SPA ingredients related to the clinical efficacy of *T. wilfordii*, we conducted a systematic study of the chemical constituents of the roots of *T. wilfordii.* As a result, ten new SPAs, wilfordatine A-J (**1**–**10**), were isolated, along with ten known analogues (Figure 1). Herein, we report the isolation and structural elucidation of these SPAs, as well as their inhibitory effects on nuclear factor-kappa B (NF-κB) pathway in the HEK293/NF-κB-Luc cells induced by Lipopolysaccharide (LPS).

## 2. Results and Discussion

The CHCl_3_-soluble fraction of the ethanol extract of the root of *T. wilfordii* was acid extracted and alkaline-precipitated to obtain the total alkaloids (TA), which were subsequently separated by ODS chromatography and preparative HPLC to afford ten new SPAs (**1**–**10**), along with ten known analogues (**11**–**20**).

**Figure 2:**
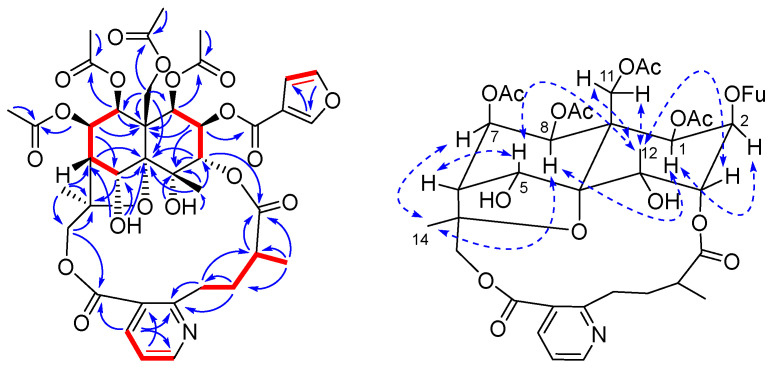
Key 2D NMR correlations of **1** [red lines for ^1^H-^1^H COSY, blue arrows for HMBC (from H to C), dashed two-way arrows for ROESY].

Compound **1** was purified as white amorphous powder with a molecular formula of C_39_H_45_NO_18_, which was inferred from the HRESIMS data (*m*/*z* 816.2715 [M + H]^+^, calcd. 816.2715). The IR spectrum indicated the presence of hydroxy (3404 cm^−1^), carbonyl (1747 cm^−1^), and ester (1257 cm^−1^) groups. The ^1^H-NMR spectroscopic data (Table 1) of **1** showed signals for three methyl groups at *δ*_H_ 1.89 (3H, s, H-12), 1.67 (3H, s, H-14), and 1.18 (3H, d, *J* = 6.6 Hz, H-10′); six oxygenated methines at *δ*_H_ 5.71 (1H, d, *J* = 3.6 Hz, H-1), 5.52 (1H, dd, *J* = 5.4, 4.2 Hz, H-7), 5.39 (1H, d, *J* = 6.0 Hz, H-8), 5.38 (1H, d, *J* = 2.4 Hz, H-5), 5.37 (1H, t, *J* = 3.0 Hz, H-2), and 5.08 (1H, d, *J* = 2.4 Hz, H-3); two sets of oxygenated methylenes at *δ*_H_ 5.43 (1H, d, *J* = 13.2 Hz, H-11a), 4.37 (1H, d, *J* = 13.2 Hz, H-11b), 5.87 (1H, d, *J* = 12.6 Hz, H-15a), and 3.73 (1H, d, *J* = 12.6 Hz, H-15b); two aliphatic methines at *δ*_H_ 2.44 (1H, d, *J* = 4.2 Hz, H-6), and 2.33 (1H, m, H-9′); two sets of aliphatic methylenes at *δ*_H_ 4.09 (1H, m, H-7a), 2.87 (1H, m, H-7b), 2.38 (1H, m, H-8a), and 1.85 (1H, m, H-8b); a 2,3-disubstituted pyridine at *δ*_H_ 8.76 (1H, d, *J* = 4.8 Hz, H-6′), 8.37 (1H, d, *J* = 7.8 Hz, H-4′), and 7.29 (1H, dd, *J* = 7.8, 4.8 Hz, H-5′); two hydroxy groups at *δ*_H_ 6.37 (1H, s, 4-OH) and 6.01 (1H, s, 5-OH); four acetyloxy groups at *δ*_H_ 2.15 (3H, s, 7-OAc), 2.07 (3H, s, 11-OAc), 1.93 (3H, s, 8-OAc), and 1.85 (3H, s, 1-OAc); and a 3-furanoyloxy group at *δ*_H_ 8.20 (1H, s, 2-OFu-2), 7.49 (1H, s, 2-OFu-5), and 6.80 (1H, t, *J* = 0.6 Hz, 2-OFu-4). The ^13^C-NMR spectroscopic data (Table 2) confirmed the presence of the aforementioned groups, in addition to showing three oxygenated quaternary carbon signals at *δ*_C_ 71.7 (C-4), 92.6 (C-10), and 85.0 (C-13); one aliphatic quaternary carbon signal at *δ*_C_ 50.8 (C-9); and seven ester carbonyl signals at *δ*_C_ 169.5 (1-OAc), 170.0 (7-OAc), 169.0 (8-OAc), 170.1 (11-OAc), 161.0 (2-OFu), 175.3 (C-11′), and 167.1 (C-12′). The ^1^H-^1^H COSY spectrum of **1** showed five isolated spin systems of H-1/H-2/H-3, H-5/H-6/H-7/H-8, H-4′/H-5′/H-6′, H-7′/H-8′/H-9′/H-10′, and H-OFu-4/H-OFu-5 (Figure 2). The first two spin systems, together with the HMBC correlations for H-1/C-8, C-9; H-2/C-4, C-9; H-3/C-4, C-10, C-12; H-5/C-10, C-13; H-6/C-10; H-7/C-9; H-8/C-1, C-9, C-11; H-11/C-1, C-8, C-9, C-10; H-12/C-3, C-4, C-10; H-14/C-6, C-13, C-15; and H-15/C-13, C-14 (Figure 2), suggested the presence of a polyoxygenated dihydroagarofuran core unit, while the next two spin systems combined with the HMBC correlations for H-4′/C-2′, C-6′, C-12′; H-5′/C-3′, C-6′; H-6′/C-2′, C-4′, C-5′; H-8′/C-2′; H-9′/C-11′; and H-10′/C-11′ (Figure 2) constructed a wilforic acid moiety. The two moieties were deduced to be linked by C-3-O-C-11′ and C-15-O-C-12′ based on the HMBC correlations for H-3/C-11′ and H-15/C-12′. Therefore, compound **1** was concluded to be a wilfordate-type SPA with one 3-furanoyloxy, four acetoxy, and two hydroxy groups attached. The positions of the ester groups were unequivocally determined by the HMBC correlations for H-1/δ_C_ 169.5 (1-OAc); H-7/δ_C_ 170.0 (7-OAc); H-8/δ_C_ 169.0 (8-OAc); H-11/δ_C_ 170.1 (11-OAc); and H-2/δ_C_ 161.0 (2-OFu). Additionally, the two hydroxy groups were assigned to C-4 and C-5, respectively, according to the HMBC correlations for 4-OH/C-4, C-12 and 5-OH/C-5, C-6, C-10.

The relative configuration of **1** was established by the ROESY experiment and coupling constant analysis. The ROESY correlations between H-8 and H-1, H-8 and H-14, and H-7 and H-14 placed these protons on the same face of the dihydroagarofuran skeleton (α-orientation). Similarly, the corrections between H-12 and H-5, H-12 and H-11, and H-12 and H-3 placed them on the other face of the dihydroagarofuran skeleton (β-orientation). Further analysis of the small coupling constants between H-1 and H-2 (*J*_1,2_ = 3.6 Hz) indicated that H-2 was equatorial. Thus, **1** was identified as 2β-furanoyloxy-1β,7β,8β,11 -tetraacetoxy-4α,5α-dihydroxy-3α,15-[2′-methyl-4′-(3′′-carboxy-2′′-pyridyl) butanoic acid] dicarbolactone dihydro-β-agarofuran and was named wilfordatine A.

Wilfordatine B (**2**) was isolated as white amorphous powder. Its molecular formula was deduced to be C_34_H_43_NO_16_ by the HRESIMS data (*m*/*z* 722.2645 [M + H]^+^, calcd. 722.2660). The ^1^H- and ^13^C-NMR data (Table 1 and Table 2) of **2** were closely similar to those of **1**, except for the absence of a set of 3-furanoyloxy group signals and a noticeable upfield chemical shift for H-2 (*δ*_H_ 3.97) compared to that (*δ*_H_ 5.37) in **1**, indicating that the furanoyloxy group at C-2 in **1** is replaced by a hydroxy group in **2**. The relative configuration of **2** was determined to be identical to that of **1** based on the ROESY correlations for H-8/H-1, H-14, H-12/H-3, H-5, and H-11 (Figure S1) as well as the coupling constant analysis. Thus, the structure of wilfordatine B (**2**) was identified to be 1β,7β,8β,11-tetraacetoxy-2β,4α,5α-trihydroxy-3α,15-[2′-methyl-4′-(3′′-carboxy-2′′-pyridyl) butanoic acid] dicarbolactone dihydro-β-agarofuran.

Wilfordatine C (**3**) was isolated as white amorphous powder with the same molecular formula of compound **2** determined by HRESIMS data (*m*/*z* 722.2670 [M + H]^+^, calcd. 722.2660). The ^1^H- and ^13^C-NMR data of **3** (Table 1 and Table 2) were comparable to those of **2**, indicating that these two compounds have the same skeleton and substituent groups, with the main differences being the locations of the ester groups. In contrast to **2** (*δ*_H_ 5.29 for H-5, *δ*_H_ 5.52 for H-7), **3** showed a downfield shift for H-5 (*δ*_H_ 6.70) but an upfield shift for H-7 (δ_H_ 4.38), suggesting an acetoxy group at C-5 and a free hydroxy group at C-7, as corroborated by the key HMBC correlation for H-5/*δ*_C_ 169.9 (5-OAc) (Figure S2). The relative configuration of **3** was determined to be the same as those of **1** and **2** by the ROESY experiment (Figure S2). Thus, the structure of wilfordatine C (**3**) was identified to be 1β,5α,8β,11-tetraacetoxy-2β,4α,7β-trihydroxy-3α,15-[2′-methyl-4′-(3′′-carboxy-2′′-pyridyl) butanoic acid] dicarbolactone dihydro-β-agarofuran.

Wilfordatine D (**4**) was isolated as white amorphous powder with a molecular formula of C_39_H_45_NO_19_ determined by HRESIMS data (*m*/*z* 832.2690 [M + H]^+^, calcd. 832.2664). A comparison of the ^1^H- and ^13^C-NMR data (Table 1 and Table 2) between **4** and **1** revealed that **4** was nearly identical to **1**, with the wilforic acid moiety being the only variation. In **1**, the proton signal of H-10′ was observed as a doublet (*J* = 6.6 Hz), whereas in **4**, it was observed as a singlet. Furthermore, **4** showed a downfield shift for C-9′ (*δ*_C_ 78.0) relative to **1** (*δ*_C_ 38.1), indicating that C-9′ in **4** was oxygenated. By analyzing the HMBC spectrum, the four acetoxy groups and the 3-furanoyloxy group were assigned to C-1, C-7, C-8, C-11, and C-2, respectively (Figure S3). Accordingly, C-9′ was assigned a hydroxy group. Thus, the structure of wilfordatine D (**4**) was identified to be 2β-furanoyloxy-1β,7β,8β,11-tetraacetoxy-4α,5α-dihydroxy-3α,15-[2′-methyl-2′-hydroxy-4′-(3′′-carboxy-2′′ -pyridyl) butanoic acid] dicarbolactone dihydro-β-agarofuran.

Wilfordatine E (**5**) was isolated as white amorphous powder with a molecular formula of C_41_H_47_NO_18_ determined by HRESIMS data (*m*/*z* 842.2870 [M + H]^+^, calcd. 842.2871). The ^1^H- and ^13^C-NMR data (Table 1 and Table 2) of **5** were analogous to those of **4**, except for the absence of a set of 3-furanoyloxy group signals and the presence of a set of benzoyloxy group signals at *δ*_H_ 8.01 (2H, d, *J* = 8.4 Hz, 2-OBz-2, 6), 7.63 (1H, t, *J* = 7.8 Hz, 2-OBz-4), and 7.50 (2H, t, *J* = 7.8 Hz, 2-OBz-3, 5). The benzoyloxy group was assigned to C-2 based on the HMBC correlations for H-2/δ_C_ 164.9 (2-OBz) (Figure S4). Thus, the structure of wilfordatine E (**5**) was identified to be 2β-benzoyloxy-1β,7β,8β,11- tetraacetoxy-4α,5α-dihydroxy-3α,15-[2′-methyl-2′-hydroxy-4′-(3′′-carboxy-2′′-pyridyl) butanoic acid] dicarbolactone dihydro-β-agarofuran.

Wilfordatine F (**6**) was isolated as white amorphous powder with a molecular formula of C_36_H_45_NO_18_ determined by HRESIMS data (*m*/*z* 780.2752 [M + H]^+^, calcd. 780.2715). The ^1^H- and ^13^C-NMR data (Table 1 and Table 2) of **6** were extremely comparable to those of **5**, with the exception of the absence of a set of benzoyloxy group signals and the presence of an extra acetyloxy group signal, indicating that the benzoyloxy group in **5** was replaced by an acetoxy group at C-2 in **6**. Thus, the structure of wilfordatine F (**6**) was identified to be 1β,2β,7β,8β,11-pentaacetoxy-4α,5α-dihydroxy-3α,15-[2′-methyl -2′-hydroxy-4′-(3′′-carboxy-2′′-pyridyl) butanoic acid] dicarbolactone dihydro-β- agarofuran.

Wilfordatine G (**7**) was isolated as white amorphous powder with a molecular formula of C_41_H_51_NO_1__9_ determined by HRESIMS data (*m*/*z* 862.3159 [M + H]^+^, calcd. 862.3134). The ^1^H- and ^13^C-NMR data (Table 1 and Table 2) of **7** were analogous to those of **5**, except for the absence of a set of benzoyloxy group signals and the existence of an extra acetoxy group signal and a set of tigloyloxy group signals at *δ*_H_ 1.88 (3H, s, 2-OTig-1), 6.96 (1H, q, *J* = 7.2 Hz, 2-OTig-3), and 1.87 (3H, d, *J* = 7.8 Hz, 2-OTig-4). Furthermore, **7** showed a downfield shift for H-5 (δ_H_ 6.95) compared to **5** (δ_H_ 5.26), indicating that C-5 was attached with an ester group. Based on the corresponding HMBC correlations, the tigloyloxy group was allocated at C-2, and the extra acetyloxy group was allocated at C-5 (Figure S6). Thus, the structure of wilfordatine G (**7**) was identified to be 1β,5α,7β,8β,11-pentaacetoxy-2β-tigloyoxy-4α-droxy-3α,15-[2′-methyl-2′-hydroxy-4′-(3′′-carboxy-2′′-pyridyl) butanoic acid] dicarbolactone dihydro-β-agarofuran.

Wilfordatine H (**8**) was isolated as white amorphous powder with a molecular formula of C_43_H_53_NO_20_ determined by HRESIMS data (*m*/*z* 904.3234 [M + H]^+^, calcd. 904.3239). The ^1^H- and ^13^C-NMR data (Table 1 and Table 2) of **8** were similar to those of **7**, but **8** contained an extra acetoxy group, and the chemical shifts of C-9′ (δ_C_ 80.5) and C-8′ (δ_C_ 21.9) were significantly different from those of **7** (δ_C_ 78.0 for C-9′, and δ_C_ 28.4 for C-8′), indicating that the hydroxy group at C-9′ was esterified. Based on the corresponding HMBC correlations, the six acetoxy groups were allocated at C-1, C-2, C-5, C-7, C-8, and C-11 (Figure S7). Accordingly, the tigloyloxy group was allocated at C-9′. Thus, the structure of wilfordatine H (**8**) was identified to be 1β,2β,5α,7β,8β,11-hexaacetoxy-4α -droxy-3α,15-[2′-methyl-2′-tigloyloxy-4′-(3′′-carboxy-2′′-pyridyl) butanoic acid] dicarbolactone dihydro-β-agarofuran.

**Figure 3:**
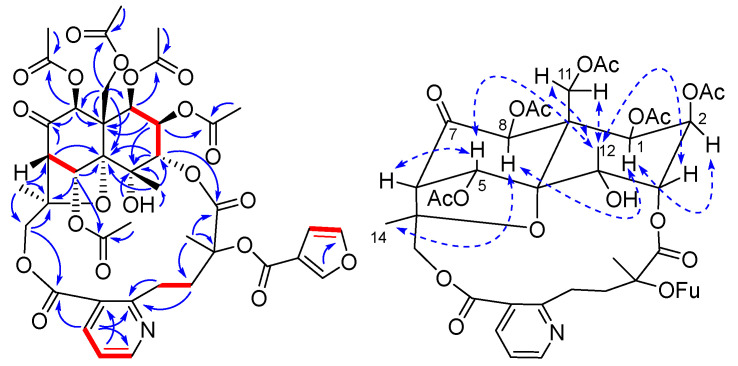
Key 2D NMR correlations of **9** [red lines for ^1^H-^1^H COSY, blue arrows for HMBC (from H to C), dashed two-way arrows for ROESY].

Wilfordatine I (**9**) was isolated as white amorphous powder with a molecular formula of C_41_H_45_NO_20_ determined by HRESIMS data (*m*/*z* 872.2642 [M + H]^+^, calcd. 872.2613). The ^1^H- and ^13^C-NMR data (Table 1 and Table 2) of **9** were analogous to those of **8**, with the significant difference that an oxygenated methine at C-7 in **8** is replaced by a keto carbonyl carbon (δ_C_ 195.5) in **9**, resulting in about 10 ppm downfield shifts of C-6 (δ_C_ 62.2) and C-8 (δ_C_ 79.4) in the ^13^C-NMR spectrum of **9**. Additionally, the key HMBC corrections for H-6/C-7 and H-8/C-7 supported the assignment of the keto carbonyl at C-7 (Figure 3). In addition, the NMR spectra of **9** lacked one set of tigloyloxy group signals and one acetoxy group signal compared to those of **8**, but it did show an additional set of 3-furoyloxy group signals at *δ*_H_ 7.82 (1H, s, 2-OFu-2), 7.32 (1H, s, 2-OFu-5), and 6.59 (1H, t, *J* = 0.6 Hz, 2-OFu-4)]. The acetoxy groups were assigned to C-1, C-2, C-8, and C-11 by the corresponding HMBC correlations (Figure 3). Accordingly, the 3-furoyloxy group was assigned to C-9′, as demonstrated by the chemical shift of C-9′ (δ_C_ 80.5) compared to that of **8** (δ_C_ 81.5). Thus, the structure of wilfordatine I (**9**) was identified to be 1β,2β,5α,8β,11-pentaacetoxy-4α-droxy-7-oxo-3α,15-[2′-methyl-2′-(3-furoyloxy)-4′-(3′′-carboxy-2′′-pyridyl) butanoic acid] dicarbolactone dihydro-β- agarofuran.

Wilfordatine J (**10**) was isolated as white amorphous powder with a molecular formula of C_43_H_47_NO_19_ determined by HRESIMS data (m/z 882.2830 [M + H]^+^, calcd. 882.2821). The ^1^H- and ^13^C-NMR data (Table 1 and Table 2) of **10** were closely similar to those of **9**, except for the absence of a set of 3-furoyloxy group signals and the presence of a set of benzoyloxy group signals at *δ*_H_ 7.81 (2H, dd, *J* = 8.4, 1.2 Hz, 9′-OBz-2, 6), 7.35 (2H, t, *J* = 7.8 Hz, 9′-OBz-3, 5), and 7.49 (1H, t, *J* = 7.8 Hz, 9′-OBz-4). The positions of the five acetoxy groups were determined by the HMBC experiment, and they were assigned to C-1, C-2, C-5, C-8, and C-11 (Figure S8). Accordingly, the benzoyloxy group was assigned to C-9′. Thus, the structure of wilfordatine J (**10**) was identified to be 1β,2β,5α,8β,11- pentaacetoxy-4α-droxy-7-oxo-3α,15-[2′-methyl-2′-(3-benzoyloxy)-4′-(3′′-carboxy-2′′-pyridyl)butanoic acid] dicarbolactone dihydro-β-agarofuran.

In addition to the ten new SPAs mentioned above, ten known analogues were also isolated from *T. wilfordii* and identified as tripfordine A (**11**) [7], wilforjine (**12**) [12], evonimine (**13**) [13], wilfortrine (**14**) [14], wilfornine A (**15**) [15], wilforine (**16**) [16], wilforzine (**17**) [17], Chiapenine ES-Ⅳ (**18**) [18], 9′-hydroxy-2-nicotinoylwilforine (**19**) [19], and tripterygiumine U (**20**) [3] by comparison of their NMR and HRMS data with the literature values.

The inhibitory effects of TA, **4**, **5**, and **9**–**16** on NF-κB pathway in HEK293/NF-κB-Luc cells induced by LPS were evaluated at a concentration of 100 μM (100 μg/mL for TA). As shown in Table 3, the tested compounds all inhibited NF-κB to varying degrees in the luciferase assay but had no effect on the cell viability in cell counting kit-8 (CCK-8) assay. It is worth noting that the NF-κB inhibitory rates of TA, **5**, **11,** and **16** were greater than 50%, and their IC_50_ values were further determined to be 7.25 μg/mL, 8.75 μM, 0.74 μM, and 15.66 μM, respectively.

SPAs are common characteristic metabolites of *T. wilfordii,* of which wilfordate-type are the most abundant. Compared to the majority of reported wilfordate-type SPAs, the new compounds reported herein have different types of ester groups and substitution positions; for example, tigloyloxy groups, which are rare in reported compounds, are first present in the structure of compounds **7** and **8**. One the other hand, the absolute configuration of wilfordate-type SPAs, particularly in C-9′ position, is a difficult problem for the current study, and only a few reports have solved it, using single-crystal X-ray crystallographic analysis [20,21]. Unfortunately, however, we did not obtained the single crystal using various solvent systems such as methanol–water. Furthermore, we attempted to determine the absolute configuration using the electronic circular dichroism (ECD) method, but due to the macrolide unit, the molecules are more flexible and have many conformations, making the calculation very difficult. Therefore, this issue needs to be further investigated.

Diterpenoids such as triptolide are currently considered as the most active components of *T. wilfordii* due to their significant immunosuppressive and anti-inflammatory activities, but the high toxicity and low content in the plant limit their further development as drug candidates [22,23]. In contrast, the current study shows that SPAs had almost no cytotoxicity, while some of them, such as compounds **5**, **11**, and **16**, had potent NF-κB inhibitory effects, although the activity may be relatively weak compared to the diterpenoids. Overall, we believe that the above SPAs, particularly **11,** are valuable for further investigation of their potential use as immunosuppressive agents.

## 3. Materials and Methods

### 3.1. General Experimental Procedures

Optical rotations were recorded on a Rudolph Research Analytical Autopol III polarimeter. UV spectra were obtained using a Shmadzu UV-2700 UV–visible spectrophotometer, and IR spectra were obtained using a Nicolet iN10 MX spectrometer. NMR experiments were conducted on a Bruker AV-600 spectrometer in CDCl_3_ with TMS as the internal standard at 25 °C. In the HMBC experiment, the ^1^*J*_C-H_ was set to 120–170 Hz, and long-range *J*_C-H_ was set to 8 Hz. In the ROESY experiment, the mixing time was set to 200 ms. HRESIMS spectra were recorded on a Waters Xevo Q-Tof MS spectrometer. Preparative HPLC was conducted on a Waters LC Prep 150 System using various column, such as Waters XBridge Prep OBD C_18_ column (30 × 150 mm, 10 μm), Waters XSelected CSH Prep C_18_ column (19 × 250 mm, 5 μm), and YMC-Pack Ph column (10 × 250 mm, 5 μm). Neutral alumina (100–200 mesh, Sinopharm Chemical Reagent Co., Ltd., Shanghai, China) and ODS (50 μm, YMC, Kyoto, Japan) were used for column chromatography.

### 3.2. Plant Material

The roots of *Tripterygium wilfordii* Hook. f. were collected in August 2020 from Hunan Province, PR China, and identified by Professor Shuai Kang, National Institutes for Food and Drug Control. A voucher specimen (No. 10106900006) has been deposited in the herbarium of Institute for Control of Chinese Traditional Medicine and Ethnic Medicine, National Institutes for Food and Drug Control, Beijing 100050, China.

### 3.3. Extraction and Isolation

The roots of *T. wilfordii* (50 kg) were powdered and extracted with 95% ethanol (250 L × 2 h × 3) under reflux. The alcohol extract was evaporated under reduced pressure to afford a residue, which was then suspended in water, and partitioned with CHCl_3_. A total of 120 g of the CHCl_3_-soluble extract was dissolved in EtOAc and partitioned three times with a 5% HCl aqueous solution. Then, ammonium hydroxide was added to the HCl aqueous layer to adjust the pH to 8~9. After filtration, the residue was dissolved with EtOAc and chromatographed over a neutral alumina column eluting with EtOAc. After recovering EtOAc by evaporation and drying, 21.36 g of the total alkaloids of *T. wilfordii* (TA) was obtained.

The TA (12.76 g) was separated by ODS chromatography with a gradient of CH_3_OH-H_2_O (35:65–100:0 *v*/*v*) to afford nine fractions (Fr.1–Fr.9). Fr. 1 (427 mg) was purified by preparative HPLC on a Waters XSelected CSH Prep C_18_ column using acetonitrile—0.05% trifluoroacetic acid aqueous solution (23:77–45:55, *v*/*v*) as mobile phase with gradient elution (detected at 220 nm, 8 mL/min), yielding five subfractions (Fr. 1-1–Fr. 1-5). Fr. 1-1 (8 mg), Fr. 1-2 (22 mg), Fr. 1-3 (12 mg), and Fr. 1-5 (143 mg) were repeatedly purified by semipreparative HPLC on a YMC-Pack Ph column using acetonitrile–H_2_O (2.5 mL/min) to afford **3** (1.25 mg, t_R_ = 21.78 min), **11** (9.90 mg, t_R_ = 24.36 min), **18** (2.01 mg, t_R_ = 25.22 min), and **12** (45.48 mg, t_R_ = 33.45 min), respectively. Fr. 2 (587 mg) was separated by preparative HPLC on a Waters XBridge Prep OBD C_18_ column using acetonitrile—H_2_O (30:70, *v*/*v*) as mobile phase (15 mL/min) to obtain six subfractions (Fr. 2-1–Fr. 2-6). Fr. 2-1 (22 mg), Fr. 2-2 (66 mg), and Fr. 2-4 (58 mg) were repeatedly purified on a Waters XSelected CSH Prep C_18_ column using acetonitrile—0.05% trifluoroacetic acid aqueous solution (8 mL/min) to afford **2** (1.71 mg, t_R_ = 31.30 min), **6** (3.74 mg, t_R_ = 35.30 min), and **20** (7.29 mg, t_R_ = 37.17 min), respectively. Similarly, Fr.4 (2.56 g), Fr.5 (3.40 g), Fr.6 (2.74 6g), and Fr.7 (1.16 g) were separated by preparative HPLC on a Waters XBridge Prep OBD C_18_ column using acetonitrile—H_2_O (35:65 for Fr.4, 40:60 for Fr.5, 45:55 for Fr.6, and 55:45 for Fr.7, *v*/*v*) as mobile phase (15 mL/min) to obtain several subfractions, which were further purified by semipreparative HPLC on a YMC-Pack Ph column. As a result, **13** (274.99 mg, t_R_ = 45.87 min) and **19** (8.58 mg, t_R_ = 52.81 min) were obtained from Fr.4; **4** (37.06 mg, t_R_ = 27.37 min) and **9** (30.75 mg, t_R_ = 51.04 min) were obtained from Fr.5; **1** (24.06 mg, t_R_ = 47.15 min), **5** (48.80 mg, t_R_ = 38.90 min), **7** (2.65 mg, t_R_ = 49.10 min), **8** (8.57 mg, t_R_ = 43.78 min), **10** (18.72 mg, t_R_ = 41.52 min), **14** (60.39 mg, t_R_ = 52.77 min), and **15** (153.36 mg, t_R_ = 56.42 min) were obtained from Fr.6; and **16** (99.69 mg, t_R_ = 32.26 min) and **17** (23.76 mg, t_R_ = 44.50 min) were obtained from Fr.7.

### 3.4. Characterization of Compounds ***1***–***10***

Wilfordatine A (**1**): white amorphous powder; ${\left[\alpha \right]}_{D}^{20}$ + 10.0 (*c* 0.05, MeOH); UV $\lambda_{max}^{MeOH}$(log *ε*): 227 (4.03), 267 (3.59) nm; IR (KBr) υ_max_: 3404, 2976, 2943, 2365, 1747, 1571, 1508, 1435, 1372, 1307, 1257, 1231, 1158, 1131, 1080, 1046, 1002, 874, 759, 603 cm^−1^; HRESIMS *m*/*z* 816.2715 [M + H]^+^ (calcd. for C_39_H_46_NO_18_, 816.2715); ^1^H- and ^13^C-NMR data, see Table 1 and Table 2.

Wilfordatine B (**2**): white amorphous powder; ${\left[\alpha \right]}_{D}^{20}$ + 9.6 (*c* 0.10, MeOH); UV $\lambda_{max}^{MeOH}$(log *ε*): 224 (3.50), 269 (3.13) nm; HRESIMS *m*/*z* 722.2645 [M + H]^+^ (calcd. for C_34_H_44_NO_16_, 722.2660); ^1^H- and ^13^C-NMR data, see Table 1 and Table 2.

Wilfordatine C (**3**): white amorphous powder; ${\left[\alpha \right]}_{D}^{20}$ − 45.3 (*c* 0.03, MeOH); UV $\lambda_{max}^{MeOH}$(log *ε*): 224 (3.96), 269 (3.56) nm; IR (KBr) υ_max_: 3469, 2922, 2851, 2361, 2342, 1741, 1647, 1585, 1434 1372, 1239, 1162, 1134, 1046, 1006, 883, 604 cm^−1^; HRESIMS *m*/*z* 722.2670 [M + H]^+^ (calcd. for C_34_H_44_NO_16_, 722.2660); ^1^H- and ^13^C-NMR data, see Table 1 and Table 2.

Wilfordatine D (**4**): white amorphous powder; ${\left[\alpha \right]}_{D}^{20}$ + 8.6 (*c* 0.06, MeOH); UV $\lambda_{max}^{MeOH}$(log *ε*): 225 (4.03), 268 (3.63) nm; IR (KBr) υ_max_: 3408, 2988, 2361, 1747, 1572, 1508, 1442, 1372, 1307, 1257, 1232, 1158, 1136, 1081, 1051, 1002, 952, 917, 874, 760, 713, 603 cm^−1^; HRESIMS *m*/*z* 832.2690 [M + H]^+^ (calcd. for C_39_H_46_NO_19_, 832.2664); ^1^H- and ^13^C-NMR data, see Table 1 and Table 2.

Wilfordatine E (**5**): white amorphous powder; ${\left[\alpha \right]}_{D}^{20}$ + 9.5 (*c* 0.07, MeOH); UV $\lambda_{max}^{MeOH}$(log *ε*): 229 (4.23), 268 (3.63) nm; IR (KBr) υ_max_: 3411, 2988, 2360, 1747, 1585, 1571, 1451, 1371, 1315,1272, 1252, 1232, 1171, 1141, 1117, 1052, 1003, 913, 870, 745, 712, 600 cm^−1^; HRESIMS *m*/*z* 842.2870 [M + H]^+^ (calcd. for C_41_H_48_NO_18_, 842.2871); ^1^H- and ^13^C-NMR data, see Table 1 and Table 2.

Wilfordatine F (**6**): white amorphous powder; ${\left[\alpha \right]}_{D}^{20}$ − 15.5 (*c* 0.05, MeOH); UV $\lambda_{max}^{MeOH}$(log *ε*): 222 (3.81), 269 (3.52) nm; IR (KBr) υ_max_: 3422, 2989, 1748, 1584, 1437, 1373, 1261, 1235, 1142, 1047, 1002, 946, 914, 870, 833, 763, 621, 603 cm^−1^; HRESIMS *m*/*z* 780.2752 [M + H]^+^ (calcd. for C_36_H_46_NO_18_, 780.2715); ^1^H- and ^13^C-NMR data, see Table 1 and Table 2.

Wilfordatine G (**7**): white amorphous powder; ${\left[\alpha \right]}_{D}^{20}$ − 12.4 (*c* 0.06, MeOH); UV $\lambda_{max}^{MeOH}$(log *ε*): 221 (4.22), 269 (3.58) nm; IR (KBr) υ_max_: 3466, 2994, 2934, 1748, 1648, 1571, 1441, 1371, 1314, 1232, 1095, 1054, 1007, 939, 883, 765, 731, 601 cm^−1^; HRESIMS *m*/*z* 862.3159 [M + H]^+^ (calcd. for C_41_H_52_NO_19_, 862.3134); ^1^H- and ^13^C-NMR data, see Table 1 and Table 2.

Wilfordatine H (**8**): white amorphous powder; ${\left[\alpha \right]}_{D}^{20}$ − 17.0 (*c* 0.05, MeOH); UV $\lambda_{max}^{MeOH}$(log *ε*): 220 (3.52), 266 (4.21) nm; IR (KBr) υ_max_: 3556, 3477, 3153, 3006, 2963, 1758, 1586, 1572, 1509, 1434, 1372, 1318, 1219, 1177, 1159, 1083, 1042, 1008, 969, 915, 874, 764, 628, 609 cm^−1^; HRESIMS *m*/*z* 904.3234 [M + H]^+^ (calcd. for C_43_H_54_NO_20_, 904.3239); ^1^H- and ^13^C-NMR data, see Table 1 and Table 2.

Wilfordatine I (**9**): white amorphous powder; ${\left[\alpha \right]}_{D}^{20}$ − 31.4 (*c* 0.06, MeOH); UV $\lambda_{max}^{MeOH}$(log *ε*): 225 (3.96), 263 (3.60) nm; IR (KBr) υ_max_: 3547, 3484, 2998, 2963, 2361, 2343, 1758, 1724, 1572, 1508, 1436, 1373, 1319, 1219, 1177, 1143, 1083, 1042, 1008, 969, 874, 765, 629, 604 cm^−1^; HRESIMS *m*/*z* 872.2642 [M + H]^+^ (calcd. for C_41_H_46_NO_20_, 872.2613); ^1^H- and ^13^C-NMR data, see Table 1 and Table 2.

Wilfordatine J (**10**): white amorphous powder; ${\left[\alpha \right]}_{D}^{20}$ − 49.2 (*c* 0.06, MeOH); UV $\lambda_{max}^{MeOH}$(log *ε*): 229 (4.21), 268 (3.67) nm; IR (KBr) υ_max_: 3548, 3483, 2998, 2943, 1756, 1725, 1586, 1569, 1452, 1437, 1372, 1288, 1248, 1218, 1135, 1084, 1042, 1008, 942, 914, 868, 760, 716, 628, 592 cm^−1^; HRESIMS *m*/*z* 882.2830 [M + H]^+^ (calcd. for C_43_H_48_NO_19_, 882.2821); ^1^H- and ^13^C-NMR data, see Table 1 and Table 2.

### 3.5. Cell Viability Assay

The viability of HEK293 cells was determined by a CCK-8 assay according to the established protocol [24]. Briefly, 5 × 10^3^ cells/well HEK293 cells were cultured in 96-well plates at 37 °C under an atmosphere of 5% CO_2_ for 24 h and then treated with the tested compounds. After 48 h of incubation, CCK-8 reagent (20 μL) was added into each well and continuously incubated for 2 h at 37 °C. Absorbance at a wavelength of 450 nm was measured by a microplate reader, which was further used to calculate the cell viability. All the experiments were performed in triplicate.

### 3.6. Immunosuppressive Activity Assay

The NF-κB inhibitory activities were measured using the HEK293/NF-κB-Luc cells, which were generated as described previously [25]. Briefly, HEK293 cells were co-transfected with a luciferase reporter plasmid, which contained an NF-κB binding site and pcDNA3.1, using Lipofectamine 2000 at a concentration of 10:1. The HEK293/ NF-κB-Luc cells were plated in 48-well plates and cultured in DMEM supplemented with 10% fetal bovine serum (FBS) for 16 h. Then, the cells were treated with the tested compounds, followed by stimulation with 1 μg/mL of LPS for 24 h. The cells were rinsed twice with phosphate-buffered saline (PBS, pH 7.4) and lysed with passive lysis buffer. Then, the inhibitory effects on NF-κB were analyzed using the luciferase assay system according to the manufacturer’s instructions. All the experiments were performed in triplicate.

## 4. Conclusions

In summary, 20 wilfordate-type SPAs, including 10 new ones and 10 known ones, were obtained from the roots of *T. wilfordii*. Their structures were mainly elucidated by spectroscopic analysis. The total alkaloids and compounds 5, 11, and 16 were found to have potent NF-κB inhibitory effects with IC_50_ values at μg/mL or μM level and no effects on the cell viability. The results obtained in this study highlight the bioactive potential of SPAs, which can be used as promising compounds for future optimization and development of potential immunosuppressive agents.

## Figures and Tables

**Table 1:** ^1^H-NMR spectroscopic data for compounds **1**–**10** (600 MHz, CDCl_3_).

Position	1	2	3	4	5	6	7	8	9	10
	*δ*_H_ (*J* in Hz)	*δ*_H_ (*J* in Hz)	*δ*_H_ (*J* in Hz)	*δ*_H_ (*J* in Hz)	*δ*_H_ (*J* in Hz)	*δ*_H_ (*J* in Hz)	*δ*_H_ (*J* in Hz)	*δ*_H_ (*J* in Hz)	*δ*_H_ (*J* in Hz)	*δ*_H_ (*J* in Hz)
1	5.71, d (3.6)	5.58, d (3.6)	5.57, d (3.6)	5.70, d (3.6)	5.76, d (3.6)	5.57, d (3.0)	5.63, d (3.6)	5.56, d (4.2)	5.65, d (3.0)	5.76, d (3.6)
2	5.37, t (3.0)	3.97, q (2.4)	3.97, t (3.0)	5.35, t (3.6)	5.47, t (3.0)	5.13, t (3.0)	5.24, t (3.0)	5.11, t (2.4)	5.17, t (3.0)	5.47, t (3.0)
3	5.08, d (2.4)	5.10, d (3.0)	5.01, d (3.0)	5.10, d (3.0)	5.16, d (3.0)	5.00, d (2.4)	4.97, d (3.0)	4.93, d (2.4)	4.95, d (2.4)	4.98, d (3.0)
5	5.38, d (2.4)	5.29, d (1.8)	6.70, s	5.33, d (3.6)	5.26, d (3.0)	5.35, s	6.95, s	6.89, s	6.66, s	6.63, s
6	2.44, d (4.2)	2.42, d (3.6)	2.36, d (3.6)	2.46, d (3.6)	2.47, d (4.2)	2.46, d (3.6)	2.36, d (4.2)	2.32, d (4.2)	2.96, s	2.96, s
7	5.52, dd (5.4, 4.2)	5.52, dd (5.4, 4.2)	4.38, d (4.2)	5.50, dd (5.4, 4.2)	5.51, t (3.0)	5.48, d (3.6)	5.52, dd (5.4, 4.2)	5.55, dd (6.0, 3.6)		
8	5.39, d (6.0)	5.34, d (6.0)	5.31, d (6.0)	5.36, d (5.4)	5.35, d (6.0)	5.32, d (6.0)	5.37, d (6.0)	5.28, d (6.0)	5.47, s	5.41, s
11a	5.43, d (13.2)	5.34, d (12.6)	5.37, d (13.2)	5.46, d (13.2)	5.47, d (13.2)	5.23, d (13.2)	5.33, d (13.2)	5.19, d (13.2)	4.82, d (13.2)	4.80, d (13.2)
11b	4.37, d (13.2)	4.61, d (12.6)	4.45, d (13.2)	4.38, d (13.2)	4.48, d (13.2)	4.52, d (13.2)	4.37, d (13.2)	4.46, d (13.2)	4.43, d (13.2)	4.43, d (13.2)
12	1.89, s	1.90, d (0.6)	1.57, s	1.94, d (1.2)	2.04, s	1.89, s	1.61, s	1.56, s	1.63, s	1.64, s
14	1.67, s	1.64, s	1.59, s	1.64, s	1.62, s	1.61, s	1.64, s	1.57, s	1.36, s	1.22, s
15a	5.87, d (12.6)	5.86, d (12.0)	5.68, d (12.0)	5.90, d (12.6)	5.90, d (12.6)	5.96, d (12.6)	5.86, d (12.0)	5.49, d (12.0)	5.80, d (12.0)	5.82, d (12.6)
15b	3.73, d (12.6)	3.72, d (12.0)	3.86, d (12.0)	3.70, d (12.6)	3.71, d (12.6)	3.73, d (12.6)	3.71, d (12.0)	3.92, d (12.0)	3.78, d (12.0)	3.72, d (12.6)
4′	8.37, d (7.8)	8.34, dd (7.8, 1.8)	8.30, dd (7.8, 1.8)	8.15, dd (7.8, 1.8)	8.16, d (7.8)	8.47, d (7.8)	8.20, d (7.8)	8.13, dd (7.8, 1.8)	8.17, dd (7.8, 1.8)	8.14, dd (7.8, 1.8)
5′	7.29, dd (7.8, 4.8)	7.28, dd (7.8, 4.8)	7.27, dd (7.8, 4.8)	7.22, dd (7.8, 4.8)	7.22, dd (7.8, 4.8)	7.52, dd (7.8, 4.8)	7.28, dd (7.8, 4.8)	7.28, dd (7.8, 4.8)	7.34, dd (7.8, 4.8)	7.35, dd (7.8, 4.8)
6′	8.76, d (4.8)	8.75, dd (4.8, 1.8)	8.74, dd (4.8, 1.8)	8.69, dd (4.8, 1.8)	8.70, d (4.8)	8.92, d (4.8)	8.75, d (4.8)	8.72, dd (4.8, 1.8)	8.77, dd (4.8, 1.8)	8.79, dd (4.8, 1.8)
7′a	4.09, m	4.05, m	3.85, m	4.09, m	4.09, m	4.22, m	4.09, m	3.61, m	3.82, m	3.83, m
7′b	2.87, m	2.87, m	2.99, m	2.84, m	2.85, m	3.10, m	2.92, m	2.95, m	2.98, m	3.00, m
8′a	2.38, m	2.33, m	2.22, m	2.45, m	2.45, m	2.69, m	2.55, m	2.65, m	2.67, m	2.73, m
8′b	1.85, m	1.87, m	2.03, m	2.16, m	2.19, m	2.10, m	2.16, m	2.20, m	2.28, m	2.36, m
9′	2.33, m	2.28, m	2.38, m							
10′	1.18, d (6.6)	1.12, d (6.6)	1.16, d (7.2)	1.48, s	1.51, s	1.45, s	1.45, s	1.73, s	1.81, s	1.84, s
1-OAc	1.85, s	1.95, s	2.00, s	1.87, s	1.87, s	1.87, s	1.86, s	1.82, s	1.80, s	2.17, s
2-OAc						2.15, s		2.15, s	2.16, s	1.65, s
5-OAc			2.16, s				2.19, s	2.16, s	2.20, s	2.20, s
7-OAc	2.15, s	2.16, s		2.17, s	2.17, s	2.17, s	2.17, s	2.15, s		
8-OAc	1.93, s	2.17, s	2.32, s	1.93, s	1.88, s	1.95, s	2.18, s	1.96, s	2.03, s	1.99, s
11-OAc	2.07, s	1.96, s	2.13, s	2.02, s	1.76, s	2.16, s	1.98, s	2.26, s	2.00, s	1.98, s
2-OFu-2	8.20, s			8.17, br s						
2-OFu-4	6.80, t (0.6)			6.78, br s						
2-OFu-5	7.49, s			7.50, t (1.8)						
2-OBz-2, 6					8.01, d (8.4)					
2-OBz-3, 5					7.50, d (7.8)					
2-OBz-4					7.63, t (7.8)					
2-OTig-1							1.88, s			
2-OTig-3							6.96, q (7.2)			
2-OTig-4							1.87, d (7.8)			
9′-OTig-1								1.75, s		
9′-OTig-3								6.71, q (6.6)		
9′-OTig-4								1.74, d (6.6)		
9′-OFu-2									7.82, s	
9′-OFu-4									6.59, d (1.2)	
9′-OFu-5									7.32, t (1.8)	
9′-OBz-2, 6										7.81, dd (8.4, 1.2)
9′-OBz-3, 5										7.35, t (7.8)
9′-OBz-4										7.49, t (7.8)
4-OH	6.37, s	6.19, d (1.2)	4.69, d (1.2)	6.30, d (1.2)	6.31, s	6.07, s	5.03, s	4.15, s	4.71, s	4.74, s
5-OH	6.01, s	6.02, d (3.6)		5.82, d (3.6)	5.81, d (3.6)					

**Table 2:** ^13^C-NMR spectroscopic data for compounds **1**–**10** (150 MHz, CDCl_3_).

Position	1	2	3	4	5	6	7	8	9	10
	*δ*_C_, Type	*δ*_C_, Type	*δ*_C_, Type	*δ*_C_, Type	*δ*_C_, Type	*δ*_C_, Type	*δ*_C_, Type	*δ*_C_, Type	*δ*_C_, Type	*δ*_C_, Type
1	73.5, CH	75.7, CH	75.0, CH	73.1, CH	73.2, CH	73.2, CH	73.2, CH	72.3, CH	70.3, CH	70.3, CH
2	69.3, CH	70.6, CH	70.4, CH	68.9, CH	69.4, CH	68.7, CH	68.6, CH	69.6, CH	69.5, CH	69.5, CH
3	75.0, CH	76.6, CH	77.6, CH	76.0, CH	76.1, CH	76.1, CH	77.0, CH	78.2, CH	77.8, CH	77.8, CH
4	71.7, C	71.8, C	69.5, C	71.6, C	71.6, C	71.8, C	69.8, C	69.7, C	69.9, C	69.9, C
5	74.2, CH	74.1, CH	72.7, CH	74.1, CH	74.1, CH	74.1, CH	73.6, CH	73.9, CH	73.8, CH	73.8, CH
6	52.5, CH	52.3, CH	53.7, CH	52.4, CH	52.3, CH	52.0, CH	51.1, CH	51.1, CH	62.2, CH	62.2, CH
7	69.1, CH	69.2, CH	67.8, CH	69.0, CH	69.2, CH	69.0, CH	68.9, CH	68.8, CH	195.5, C	195.7, C
8	71.0, CH	71.5, CH	73.9, CH	70.9, CH	71.2, CH	70.9, CH	70.7, CH	71.6, CH	79.4, CH	79.4, CH
9	50.8, C	51.1, C	53.4, C	50.7, C	50.5, C	50.8, C	52.0, C	52.2, C	52.5, C	52.5, C
10	92.6, C	93.0, C	93.8, C	93.0, C	93.0, C	93.5, C	94.3, C	93.4, C	94.9, C	94.9, C
11	60.9, CH_2_	61.1, CH_2_	61.3, CH_2_	60.9, CH_2_	61.1, CH_2_	60.5, CH_2_	60.4, CH_2_	60.3, CH_2_	60.2, CH_2_	60.2, CH_2_
12	23.4, CH_3_	23.7, CH_3_	23.1, CH_3_	23.4, CH_3_	23.9, CH_3_	23.1, CH_3_	22.5, CH_3_	23.2, CH_3_	24.1, CH_3_	24.1, CH_3_
13	85.0, C	84.9, C	84.2, C	85.3, C	85.4, C	85.1, C	84.7, C	84.2, C	86.4, C	86.4, C
14	18.2, CH_3_	18.2, CH_3_	17.8, CH_3_	18.0, CH_3_	18.0, CH_3_	18.4, CH_3_	17.9, CH_3_	17.8, CH_3_	18.2, CH_3_	18.1, CH_3_
15	71.1, CH_2_	71.1, CH_2_	70.4, CH_2_	70.7, CH_2_	70.7, CH_2_	71.5, CH_2_	70.0, CH_2_	70.2, CH_2_	70.3, CH_2_	70.2, CH_2_
2′	165.2, C	164.9, C	163.5, C	165.4, C	165.3, C	164.8, C	164.9, C	161.5, C	162.4, C	162.2, C
3′	123.8, C	124.1, C	124.8, C	125.1, C	124.1, C	127.6, C	123.9, C	125.0, C	125.0, C	125.1, C
4′	138.7, CH	138.6, CH	138.8, CH	137.9, CH	137.9, CH	141.5, CH	138.5, CH	138.6, CH	138.5, CH	138.2, CH
5′	121.2, CH	121.2, CH	121.2, CH	120.7, CH	120.7, CH	122.3, CH	120.9, CH	121.3, CH	121.4, CH	121.3, CH
6′	153.6, CH	153.4, CH	152.9, CH	152.5, CH	152.5, CH	148.2, CH	152.5, CH	152.4, CH	152.4, CH	152.6, CH
7′	33.0, CH_2_	32.9, CH_2_	33.5, CH_2_	31.5, CH_2_	31.6, CH_2_	28.7, CH_2_	38.2, CH_2_	38.1, CH_2_	37.9, CH_2_	38.1, CH_2_
8′	33.5, CH_2_	33.5, CH_2_	33.2, CH_2_	39.0, CH_2_	39.1, CH_2_	38.4, CH_2_	33.5, CH_2_	33.2, CH_2_	39.0, CH_2_	39.1, CH_2_
9′	38.1, CH	38.3, CH	38.7, CH	78.0, C	77.9, C	78.2, C	78.0, C	80.5, C	81.5, C	81.8, C
10′	19.0, CH_3_	18.8, CH_3_	18.5, CH_3_	27.5, CH_3_	27.3, CH_3_	28.5, CH_3_	28.4, CH_3_	21.9, CH_3_	22.1, CH_3_	22.1, CH_3_
11′	175.3, C	175.7, C	175.6, C	172.6, C	172.6, C	172.0, C	172.3, C	171.6, C	171.2, C	171.3, C
12′	167.1, C	167.1, C	167.2, C	168.4, C	168.3, C	166.7, C	167.5, C	167.7, C	167.4, C	167.5, C
1-OAc	20.5, CH_3_ /169.5, C	20.5, CH_3_ /169.5, C	20.9, CH_3_ /169.5, C	20.5, CH_3_ /169.9, C	20.3, CH_3_ /170.2, C	20.5, CH_3_ /169.9, C	20.5, CH_3_ /169.6, C	20.3, CH_3_ /168.1, C	20.0, CH_3_ /168.0, C	20.0, CH_3_ /168.0, C
2-OAc						21.3, CH_3_ /168.6, C		21.0, CH_3_ /168.3, C	21.0, CH_3_ /168.1, C	21.0, CH_3_ /167.8, C
5-OAc			21.6, CH_3_ /169.9, C				21.6, CH_3_ /169.8, C	21.3, CH_3_ /169.8, C	21.4, CH_3_ /169.3, C	21.4, CH_3_ /169.3, C
7-OAc	21.0, CH_3_ /170.0, C	21.0, CH_3_ /170.0, C		21.0, CH_3_ /170.0, C	20.9, CH_3_ /170.0, C	21.0, CH_3_ /170.0, C	21.0, CH_3_ /170.1, C	21.1, CH_3_ /170.0, C		
8-OAc	20.4, CH_3_ /169.0, C	21.5, CH_3_ /169.4, C	21.1, CH_3_ /169.3, C	20.4, CH_3_ /169.0, C	20.6, CH_3_ /169.0, C	20.4, CH_3_ /169.0, C	20.5, CH_3_ /169.0, C	20.4, CH_3_ /168.8, C	20.2, CH_3_ /168.8, C	20.2, CH_3_ /168.8, C
11-OAc	21.1, CH_3_ /170.1, C	20.8, CH_3_ /169.1, C	20.9, CH_3_ /169.2, C	21.0, CH_3_ /170.0, C	20.7, CH_3_ /169.7, C	21.0, CH_3_ /169.6, C	21.2, CH_3_ /170.3, C	21.3, CH_3_ /170.2, C	20.4, CH_3_ /169.6, C	20.4, CH_3_ /169.6, C
2-OFu-2	148.5, CH			148.5, CH						
2-OFu-3	118.4, C			118.3, C						
2-OFu-4	109.7, CH			109.6, CH						
2-OFu-5	144.3, CH			144.4, CH						
2-OFu-6	161.0, C			160.9, C						
2-OBz-1					128.8, C					
2-OBz-2, 6					129.7, CH					
2-OBz-3, 5					128.8, CH					
2-OBz-4					133.9, CH					
2-OBz-7					164.9, C					
2-OTig-1							12.2, CH_3_			
2-OTig-2							127.4, CH			
2-OTig-3							140.0, C			
2-OTig-4							14.7, CH_3_			
2-OTig-5							166.0, C			
9′-OTig-1								11.7, CH_3_		
9′-OTig-2								128.4, CH		
9′-OTig-3								138.9, C		
9′-OTig-4								14.5, CH_3_		
9′-OTig-5								167.4, C		
9′-OFu-2									149.0, CH	
9′-OFu-3									118.7, C	
9′-OFu-4									110.2, CH	
9′-OFu-5									143.3, CH	
9′-OFu-6									162.1, C	
9′-OBz-1										129.5, C
9′-OBz-2, 6										130.2, CH
9′-OBz-3, 5										128.2, CH
9′-OBz-4										133.1, CH
9′-OBz-7										165.8, C

**Table 3:** NF-κB inhibitory effects of TA, **4**, **5**, and **9**–**16** in the HEK293/NF-κB-Luc cells induced by LPS.

Compounds	Cell Viability ^a^ (%) (N = 3)	NF-κB Inhibitory Rates (%) (N = 3)	IC_50_ (μM)
TA	95.59 ± 6.56	64.22 ± 4.53	7.25 ^b^
**4**	100.94 ± 3.58	7.27 ± 2.28	
**5**	93.85 ± 2.20	65.17 ± 6.12	8.75
**9**	99.51 ± 5.71	37.43 ± 1.99	
**10**	102.85 ± 0.89	21.73 ± 4.42	
**11**	101.60 ± 3.66	64.61 ± 5.15	0.74
**12**	93.50 ± 2.76	23.04 ± 3.43	
**13**	100.13 ± 6.40	26.49 ± 5.99	
**14**	93.76 ± 1.82	40.20 ± 3.92	
**15**	110.06 ± 5.83	8.73 ± 2.07	
**16**	102.76 ± 2.21	69.07 ± 4.36	15.66
Blank control	100.00 ± 3.33		
JSH23 ^c^		74.56 ± 1.83	

^a^ TA was tested at a concentration of 100 μg/mL, and compounds **4**, **5**, and **9**–**16** were tested at the concentration of 100 μM. ^b^ The unit of IC_50_ value of TA are expressed as μg/mL. ^c^ JSH23 was used as positive control for NF-κB inhibition.

## Data Availability

The data presented in this study are available in article and Supplementary Materials.

## Supplementary Materials

The following supporting information can be downloaded at: https://www.mdpi.com/article/10.3390/molecules27217274/s1. Figures S1–S8: Key 2D NMR correlations of compounds **2**–**8** and **10**; Figures S9–S97: 1D-, 2D-NMR, HRESIMS, UV, and IR spectra for the new compounds except for the IR spectrum of **2**; Figures S98–S117: ^1^H- and ^13^C-NMR spectra for the known compounds.

## Sample Availability

Not available.
