# Supplementary material for: Immunosuppressive Sesquiterpene Pyridine Alkaloids from Tripterygium wilfordii Hook. f
Source: Molecules. 2022 Oct 26;27(21):7274. doi: 10.3390/molecules27217274 (PMC9654820; doi:10.3390/molecules27217274)
Supplement: Supplementary file 1 [file molecules-27-07274-s001.zip › molecules-1957488-supplementary.pdf]

molecules

Supplementary Materials

Immunosuppressive Sesquiterpene Pyridine Alkaloids from *Tripterygium wilfordii* Hook. f.

Yadan Wang [1,†], Jiangong Yan [1,†], Zhongmou Zhang [1,2], Minghui Chen [1], Xianfu Wu [1,3,*] and Shuangcheng Ma [1,*]

[1] National Institutes for Food and Drug Control, Beijing 102629, China
[2] School of Traditional Chinese Pharmacy, China Pharmaceutical University, Nanjing 211100, China
[3] State Key Laboratory of Bioactive Substance and Function of Natural Medicines, Institute of Materia Medica, Chinese Academy of Medical Sciences and Peking Union Medical College, Beijing 100050, China
* Correspondence: wuxf99@163.com (X.W.); masc@nifdc.org.cn (S.M.); Tel.: +86-10-5385-2026 (X.W.); +86-10-5385-2076 (S.M.)
† These authors contributed equally to this work.

Supplementary data

Figure S1: Key 2D NMR correlations of compound **2** 6
Figure S2: Key 2D NMR correlations of compound **3** 6
Figure S3: Key 2D NMR correlations of compound **4** 6
Figure S4: Key 2D NMR correlations of compound **5** 7
Figure S5: Key 2D NMR correlations of compound **6** 7
Figure S6: Key 2D NMR correlations of compound **7** 7
Figure S7: Key 2D NMR correlations of compound **8** 8
Figure S8: Key 2D NMR correlations of compound **10** 8
Figure S9: UV spectrum of compound **1** 8
Figure S10: IR spectrum of compound **1** 9
Figure S11: HRESIMS spectrum of compound **1** 9
Figure S12: ^{1}H-NMR spectrum of compound **1** ($CDCl_3$, 600 MHz) 10
Figure S13: ^{13}C-NMR spectrum of compound **1** ($CDCl_3$, 150 MHz) 10
Figure S14: ^{1}H-^{1}H COSY spectrum of compound **1** 11
Figure S15: HSQC spectrum of compound **1** 11
Figure S16: HMBC spectrum of compound **1** 12
Figure S17: ROESY spectrum of compound **1** 12
Figure S18: UV spectrum of compound **2** 13
Figure S19: HRESIMS spectrum of compound **2** 13
Figure S20: ^{1}H-NMR spectrum of compound **2** ($CDCl_3$, 600 MHz) 14
Figure S21: ^{13}C-NMR spectrum of compound **2** ($CDCl_3$, 150 MHz) 14
Figure S22: ^{1}H-^{1}H COSY spectrum of compound **2** 15
Figure S23: HSQC spectrum of compound **2** 15
Figure S24: HMBC spectrum of compound **2** 16
Figure S25: ROESY spectrum of compound **2** 16
Figure S26: UV spectrum of compound **3** 17
Figure S27: IR spectrum of compound **3** 17
Figure S28: HRESIMS spectrum of compound **3** 17
Figure S29: ^{1}H-NMR spectrum of compound **3** ($CDCl_3$, 600 MHz) 18

Figure S30: ^{13}C-NMR spectrum of compound **3** ($CDCl_3$, 150 MHz) 18
Figure S31: ^{1}H-^{1}H COSY spectrum of compound **3** 19
Figure S32: HSQC spectrum of compound **3** 19
Figure S33: HMBC spectrum of compound **3** 20
Figure S34: ROESY spectrum of compound **3** 20
Figure S35: UV spectrum of compound **4** 21
Figure S36: IR spectrum of compound **4** 21
Figure S37: HRESIMS spectrum of compound **4** 21
Figure S38: ^{1}H-NMR spectrum of compound **4** ($CDCl_3$, 600 MHz) 22
Figure S39: ^{13}C-NMR spectrum of compound **4** ($CDCl_3$, 150 MHz) 22
Figure S40: ^{1}H-^{1}H COSY spectrum of compound **4** 23
Figure S41: HSQC spectrum of compound **4** 23
Figure S42: HMBC spectrum of compound **4** 24
Figure S43: ROESY spectrum of compound **4** 24
Figure S44: UV spectrum of compound **5** 25
Figure S45: IR spectrum of compound **5** 25
Figure S46: HRESIMS spectrum of compound **5** 25
Figure S47: ^{1}H-NMR spectrum of compound **5** ($CDCl_3$, 600 MHz) 26
Figure S48: ^{13}C-NMR spectrum of compound **5** ($CDCl_3$, 150 MHz) 26
Figure S49: ^{1}H-^{1}H COSY spectrum of compound **5** 27
Figure S50: HSQC spectrum of compound **5** 27
Figure S51: HMBC spectrum of compound **5** 28
Figure S52: ROESY spectrum of compound **5** 28
Figure S53: UV spectrum of compound **6** 29
Figure S54: IR spectrum of compound **6** 29
Figure S55: HRESIMS spectrum of compound **6** 29
Figure S56: ^{1}H-NMR spectrum of compound **6** ($CDCl_3$, 600 MHz) 30
Figure S57: ^{13}C-NMR spectrum of compound **6** ($CDCl_3$, 150 MHz) 30
Figure S58: ^{1}H-^{1}H COSY spectrum of compound **6** 31
Figure S59: HSQC spectrum of compound **6** 31
Figure S60: HMBC spectrum of compound **6** 32
Figure S61: ROESY spectrum of compound **6** 32

Figure S62: UV spectrum of compound **7** 33

Figure S63: IR spectrum of compound **7**..33

Figure S64: HRESIMS spectrum of compound **7**..33

Figure S65: ^{1}H-NMR spectrum of compound **7** ($CDCl_3$, 600 MHz)..................................34

Figure S66: ^{13}C-NMR spectrum of compound **7** ($CDCl_3$, 150 MHz) 34

Figure S67: ^{1}H-^{1}H COSY spectrum of compound **7**..35

Figure S68: HSQC spectrum of compound **7**.. 35

Figure S69: HMBC spectrum of compound **7**.. 36

Figure S70: ROESY spectrum of compound **7**...36

Figure S71: UV spectrum of compound **8**.. 37

Figure S72: IR spectrum of compound **8**..37

Figure S73: HRESIMS spectrum of compound **8**..37

Figure S74: ^{1}H-NMR spectrum of compound **8** ($CDCl_3$, 600 MHz)..................................38

Figure S75: ^{13}C-NMR spectrum of compound **8** ($CDCl_3$, 150 MHz) 38

Figure S76 ^{1}H-^{1}H COSY spectrum of compound **8**..39

Figure S77: HSQC spectrum of compound **8**.. 39

Figure S78: HMBC spectrum of compound **8**.. 40

Figure S79: ROESY spectrum of compound **8**...40

Figure S80: UV spectrum of compound **9**.. 41

Figure S81: IR spectrum of compound **9**..41

Figure S82: HRESIMS spectrum of compound **9**..41

Figure S83: ^{1}H-NMR spectrum of compound **9** ($CDCl_3$, 600 MHz)..................................42

Figure S84: ^{13}C-NMR spectrum of compound **9** ($CDCl_3$, 150 MHz) 42

Figure S85: ^{1}H-^{1}H COSY spectrum of compound **9**..43

Figure S86: HSQC spectrum of compound **9**.. 43

Figure S87: HMBC spectrum of compound **9**.. 44

Figure S88: ROESY spectrum of compound **9**...44

Figure S89: UV spectrum of compound **10**.. 45

Figure S90: IR spectrum of compound **10**..45

Figure S91: HRESIMS spectrum of compound **10**..45

Figure S92: ^{1}H-NMR spectrum of compound **10** ($CDCl_3$, 600 MHz)................................46

Figure S93: ^{13}C-NMR spectrum of compound **10** ($CDCl_3$, 150 MHz) 46

Figure S94: ^{1}H-^{1}H COSY spectrum of compound **10**..47
Figure S95: HSQC spectrum of compound **10**..47
Figure S96: HMBC spectrum of compound **10**..48
Figure S97: ROESY spectrum of compound **10**..48
Figure S98: ^{1}H-NMR spectrum of compound **11** ($CDCl_3$, 600 MHz)..................................49
Figure S99: ^{13}C-NMR spectrum of compound **11** ($CDCl_3$, 150 MHz)..................................49
Figure S100: ^{1}H-NMR spectrum of compound **12** ($CDCl_3$, 600 MHz)................................50
Figure S101: ^{13}C-NMR spectrum of compound **12** ($CDCl_3$, 150 MHz)................................50
Figure S102: ^{1}H-NMR spectrum of compound **13** ($CDCl_3$, 600 MHz)................................51
Figure S103: ^{13}C-NMR spectrum of compound **13** ($CDCl_3$, 150 MHz)................................51
Figure S104: ^{1}H-NMR spectrum of compound **14** ($CDCl_3$, 600 MHz)................................52
Figure S105: ^{13}C-NMR spectrum of compound **14** ($CDCl_3$, 150 MHz)................................52
Figure S106: ^{1}H-NMR spectrum of compound **15** ($CDCl_3$, 600 MHz)................................53
Figure S107: ^{13}C-NMR spectrum of compound **15** ($CDCl_3$, 150 MHz)................................53
Figure S108: ^{1}H-NMR spectrum of compound **16** ($CDCl_3$, 600 MHz)................................54
Figure S109: ^{13}C-NMR spectrum of compound **16** ($CDCl_3$, 150 MHz)................................54
Figure S110: ^{1}H-NMR spectrum of compound **17** ($CDCl_3$, 600 MHz)................................55
Figure S111: ^{13}C-NMR spectrum of compound **17** ($CDCl_3$, 150 MHz)................................55
Figure S112: ^{1}H-NMR spectrum of compound **18** ($CDCl_3$, 600 MHz)................................56
Figure S113: ^{13}C-NMR spectrum of compound **18** ($CDCl_3$, 150 MHz)................................56
Figure S114: ^{1}H-NMR spectrum of compound **19** ($CDCl_3$, 600 MHz)................................57
Figure S115: ^{13}C-NMR spectrum of compound **19** ($CDCl_3$, 150 MHz)................................57
Figure S116: ^{1}H-NMR spectrum of compound **20** ($CDCl_3$, 600 MHz)................................58
Figure S117: ^{13}C-NMR spectrum of compound **20** ($CDCl_3$, 150 MHz)................................58
Figure S118: NF-κB inhibitory effect of Total alkaloids of *T. wilfordii* (TA).................. 59
Figure S119: NF-κB inhibitory effect of compound **5**..59
Figure S120: NF-κB inhibitory effect of compound **11**..60
Figure S121: NF-κB inhibitory effect of compound **16**..60

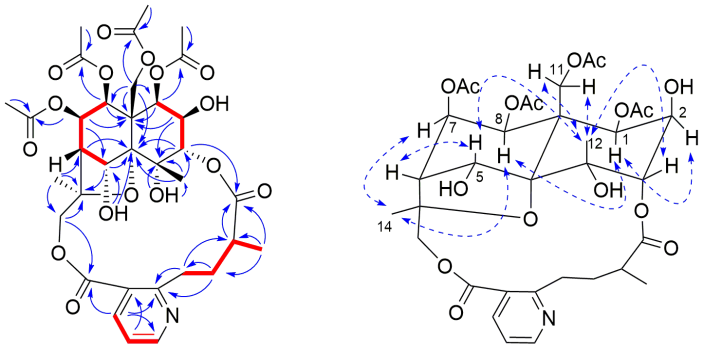

Figure S1. Key 2D NMR correlations of **2** [red lines for ^{1}H-^{1}H COSY, blue arrows for HMBC (from H to C), dashed two-way arrows for ROESY]

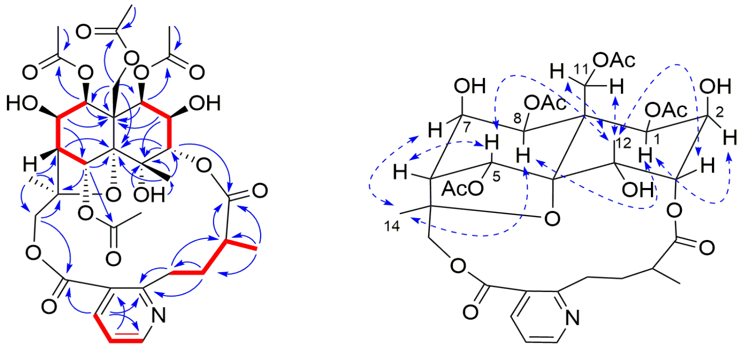

Figure S2. Key 2D NMR correlations of **3** [red lines for ^{1}H-^{1}H COSY, blue arrows for HMBC (from H to C), dashed two-way arrows for ROESY]

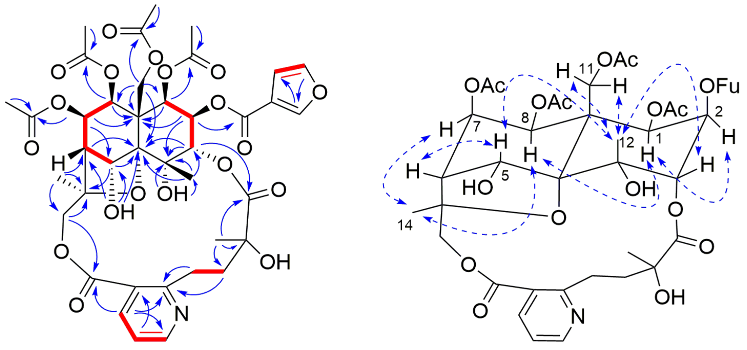

Figure S3. Key 2D NMR correlations of **4** [red lines for ^{1}H-^{1}H COSY, blue arrows for HMBC (from H to C), dashed two-way arrows for ROESY]

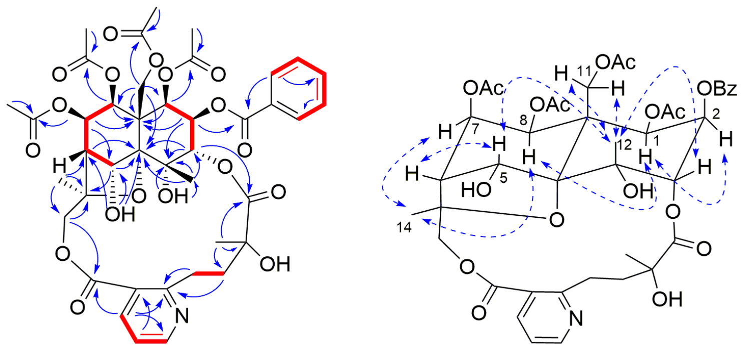

Figure S4. Key 2D NMR correlations of **5** [red lines for ^{1}H-^{1}H COSY, blue arrows for HMBC (from H to C), dashed two-way arrows for ROESY]

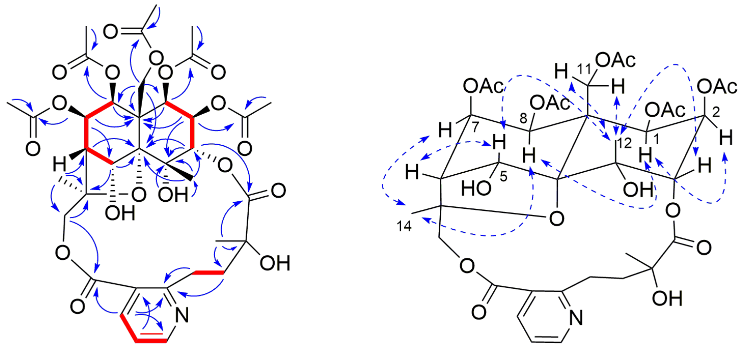

Figure S5. Key 2D NMR correlations of **6** [red lines for ^{1}H-^{1}H COSY, blue arrows for HMBC (from H to C), dashed two-way arrows for ROESY]

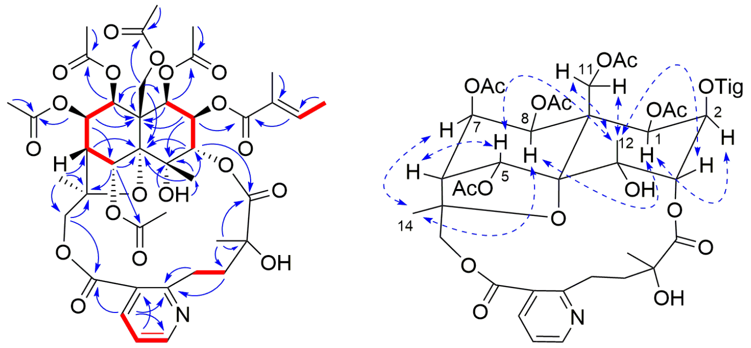

Figure S6. Key 2D NMR correlations of **7** [red lines for ^{1}H-^{1}H COSY, blue arrows for HMBC (from H to C), dashed two-way arrows for ROESY]

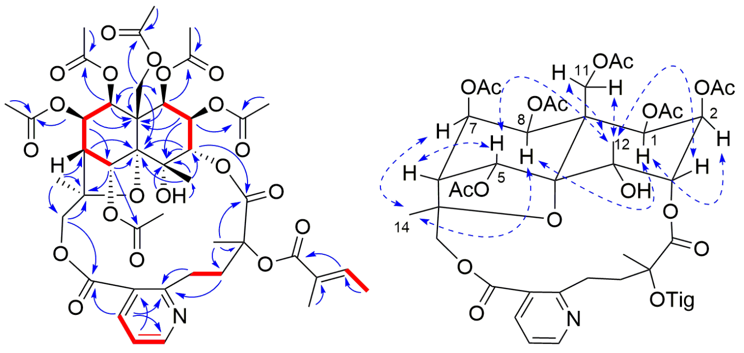

Figure S7. Key 2D NMR correlations of **8** [red lines for ^{1}H-^{1}H COSY, blue arrows for HMBC (from H to C), dashed two-way arrows for ROESY]

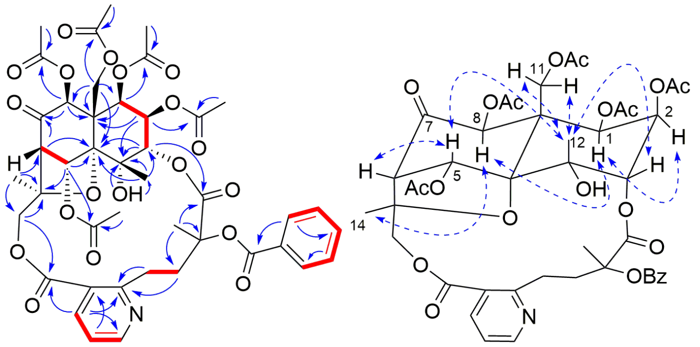

Figure S8. Key 2D NMR correlations of **10** [red lines for ^{1}H-^{1}H COSY, blue arrows for HMBC (from H to C), dashed two-way arrows for ROESY]

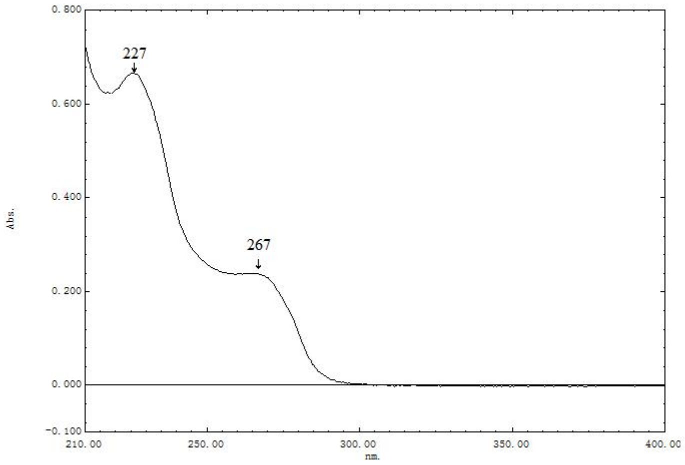

Figure S9. UV spectrum of compound **1**

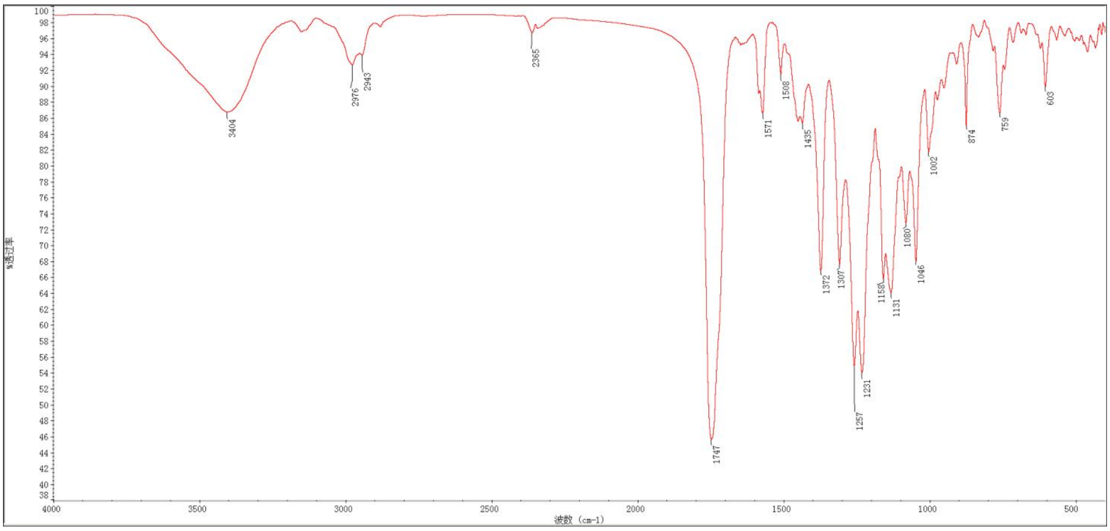

Figure S10. IR spectrum of compound **1**

Single Mass Analysis
Tolerance = 5.0 mDa / DBE: min = -1.5, max = 50.0
Element prediction: Off
Monoisotopic Mass, Even Electron Ions
623 formula(e) evaluated with 3 results within limits (up to 50 closest results for each mass)
Elements Used:
C: 0-50 H: 0-100 N: 1-3 O: 1-50

Mass	Calc. Mass	mDa	PPM	DBE	Formula	C	H	N	O
816.2715	816.2715	0.0	0.0	17.5	C39 H46 N O18	39	46	1	18
	816.2733	-1.8	-2.2	4.5	C27 H50 N3 O25	27	50	3	25
	816.2675	4.0	4.9	13.5	C34 H46 N3 O20	34	46	3	20

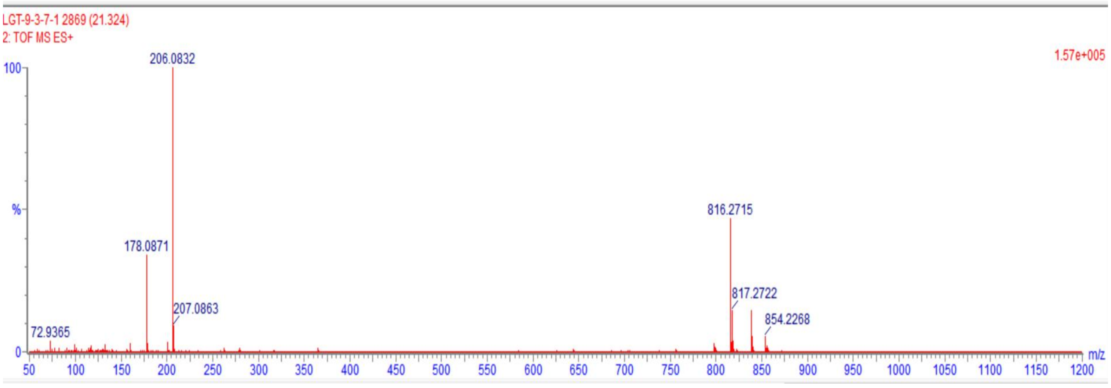

Figure S11. HRESIMS spectrum of compound **1**

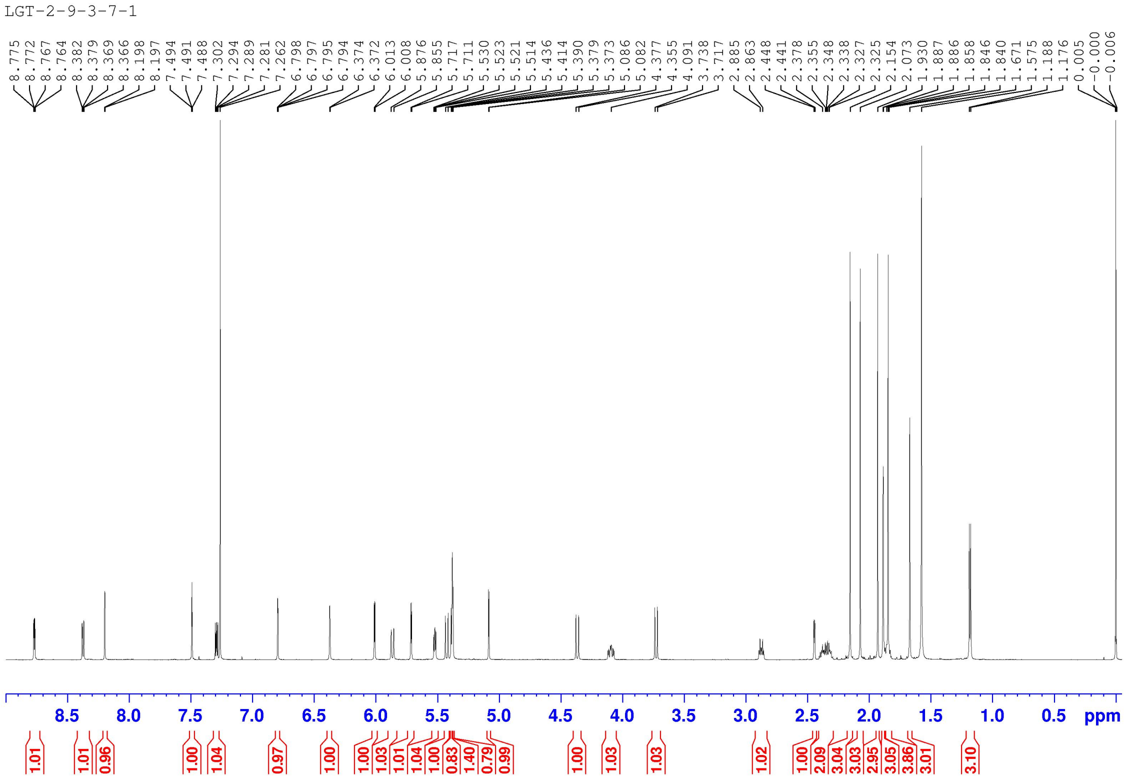

Figure S12. ^{1}H-NMR spectrum of compound **1** ($CDCl_3$, 600 MHz)

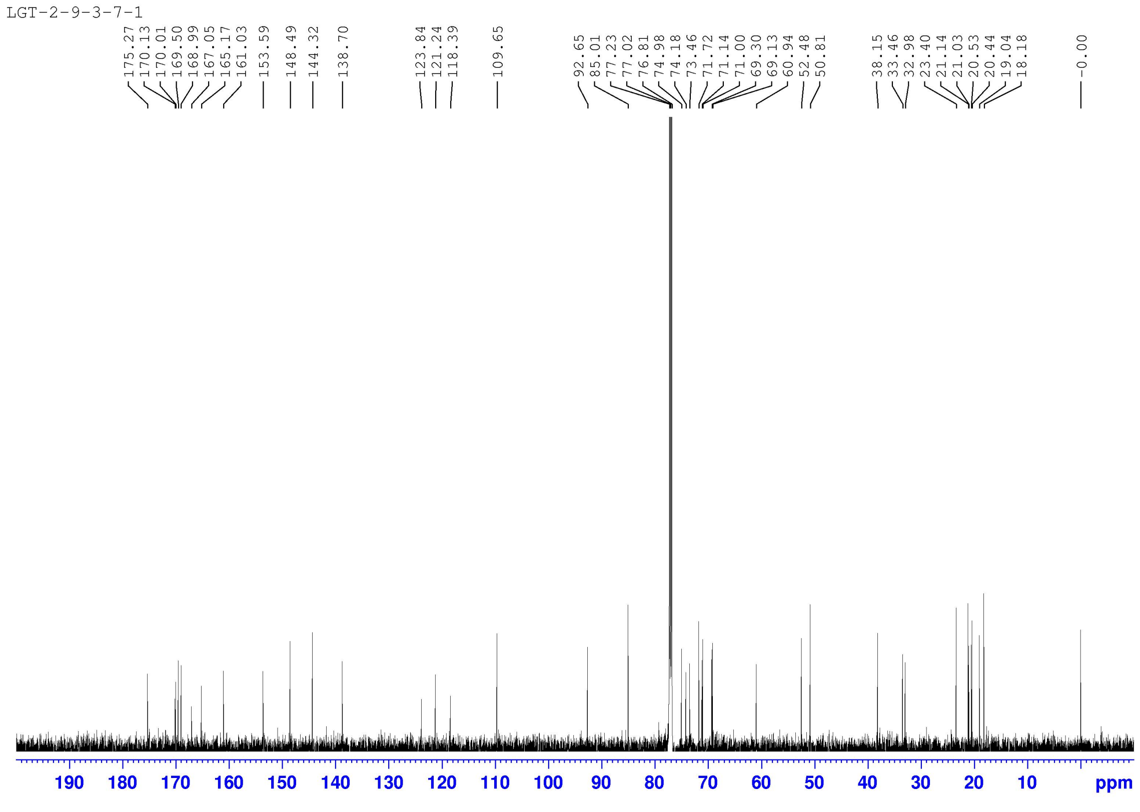

Figure S13. ^{13}C-NMR spectrum of compound **1** ($CDCl_3$, 150 MHz)

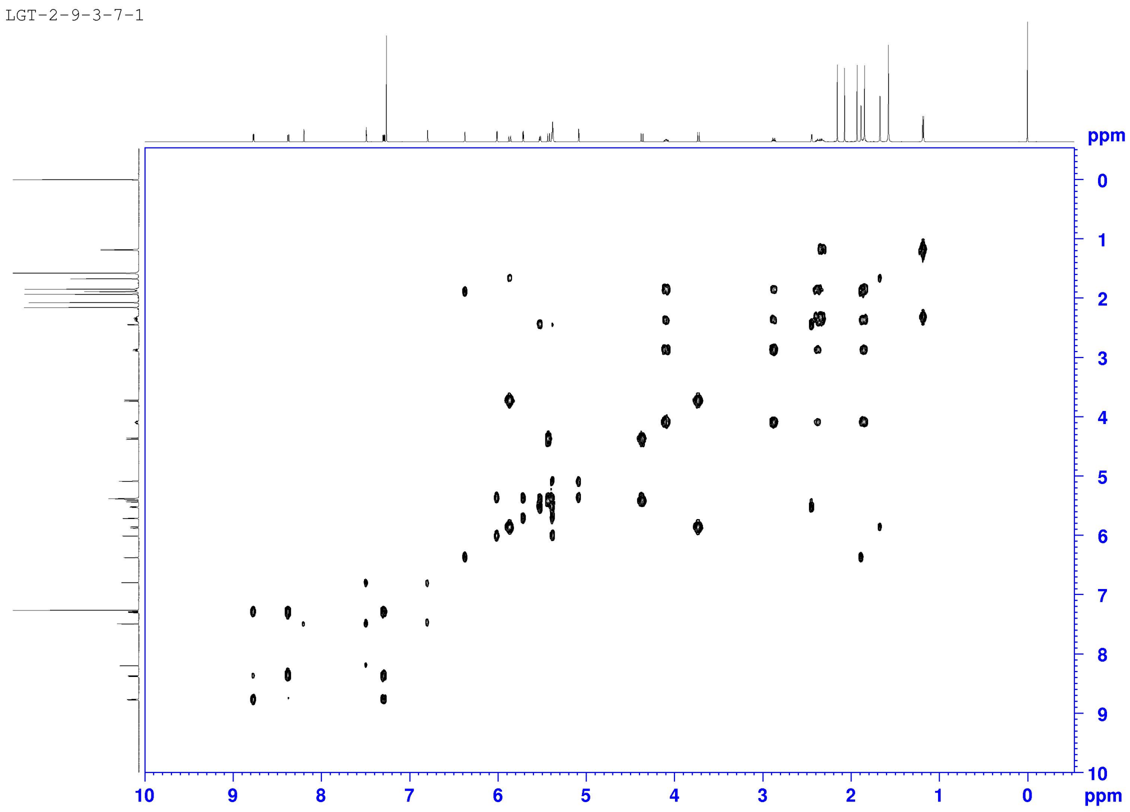

Figure S14. ^{1}H-^{1}H COSY spectrum of compound **1**

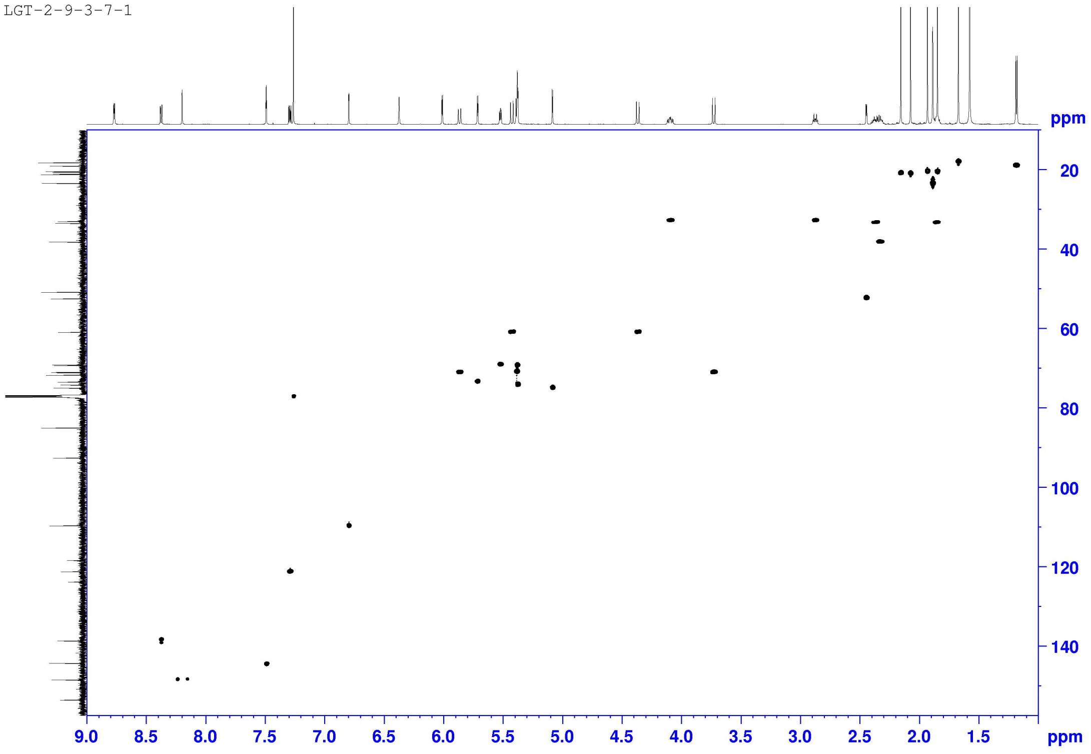

Figure S15. HSQC spectrum of compound **1**

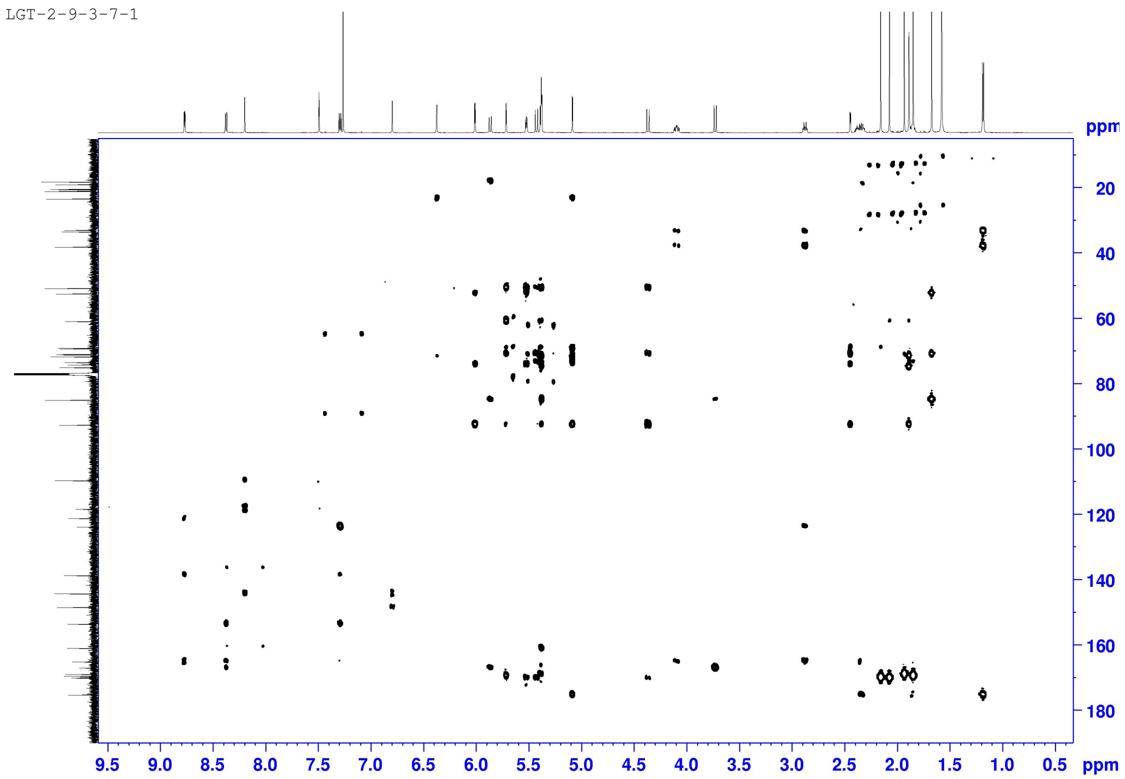

Figure S16. HMBC spectrum of compound **1**

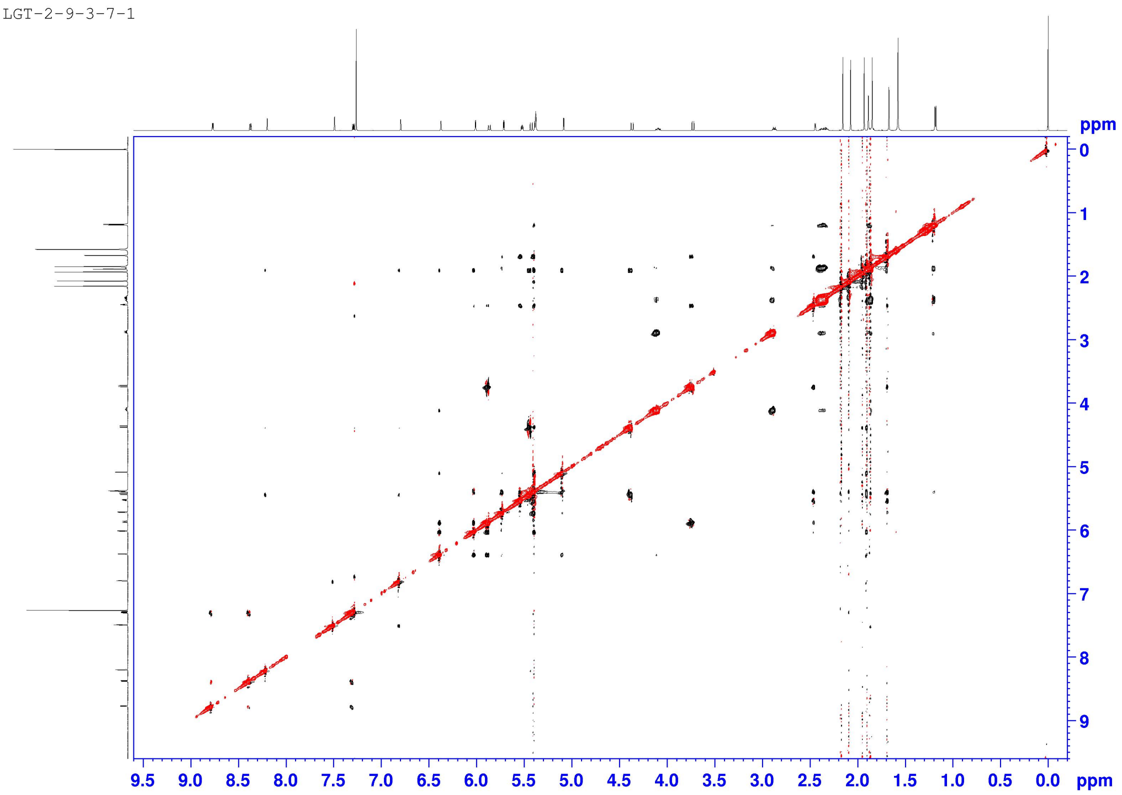

Figure S17. ROESY spectrum of compound **1**

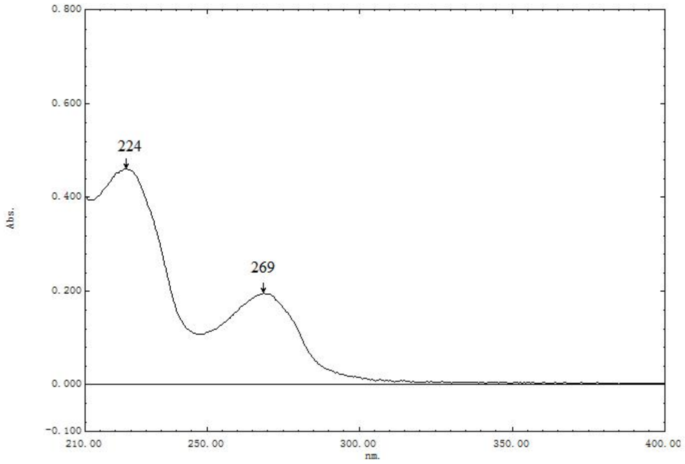

Figure S18. UV spectrum of compound **2**

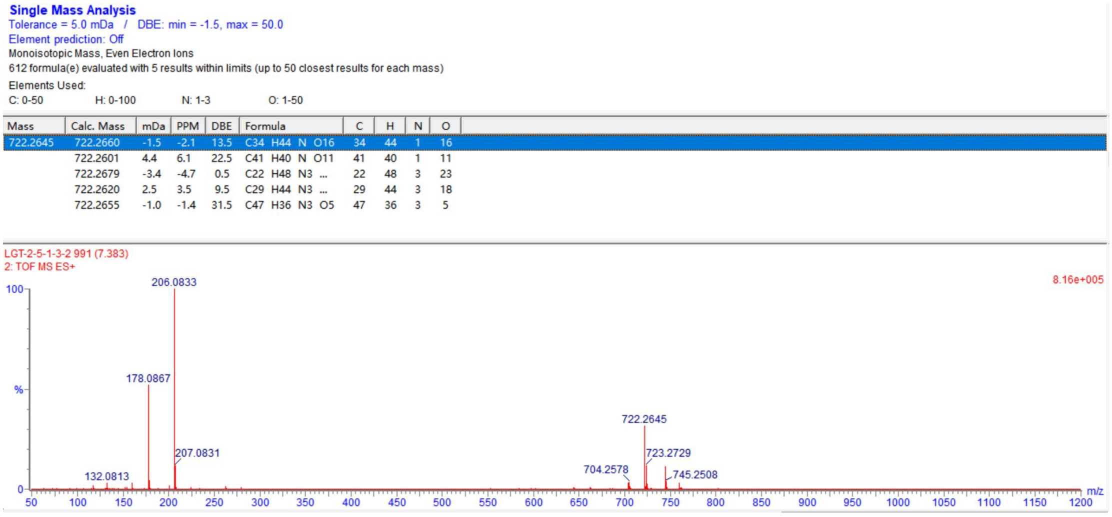

Mass	Calc. Mass	mDa	PPM	DBE	Formula	C	H	N	O
722.2645	722.2660	-1.5	-2.1	13.5	C34 H44 N O16	34	44	1	16
	722.2601	4.4	6.1	22.5	C41 H40 N O11	41	40	1	11
	722.2679	-3.4	-4.7	0.5	C22 H48 N3 ...	22	48	3	23
	722.2620	2.5	3.5	9.5	C29 H44 N3 ...	29	44	3	18
	722.2655	-1.0	-1.4	31.5	C47 H36 N3 O5	47	36	3	5

Figure S19. HRESIMS spectrum of compound **2**

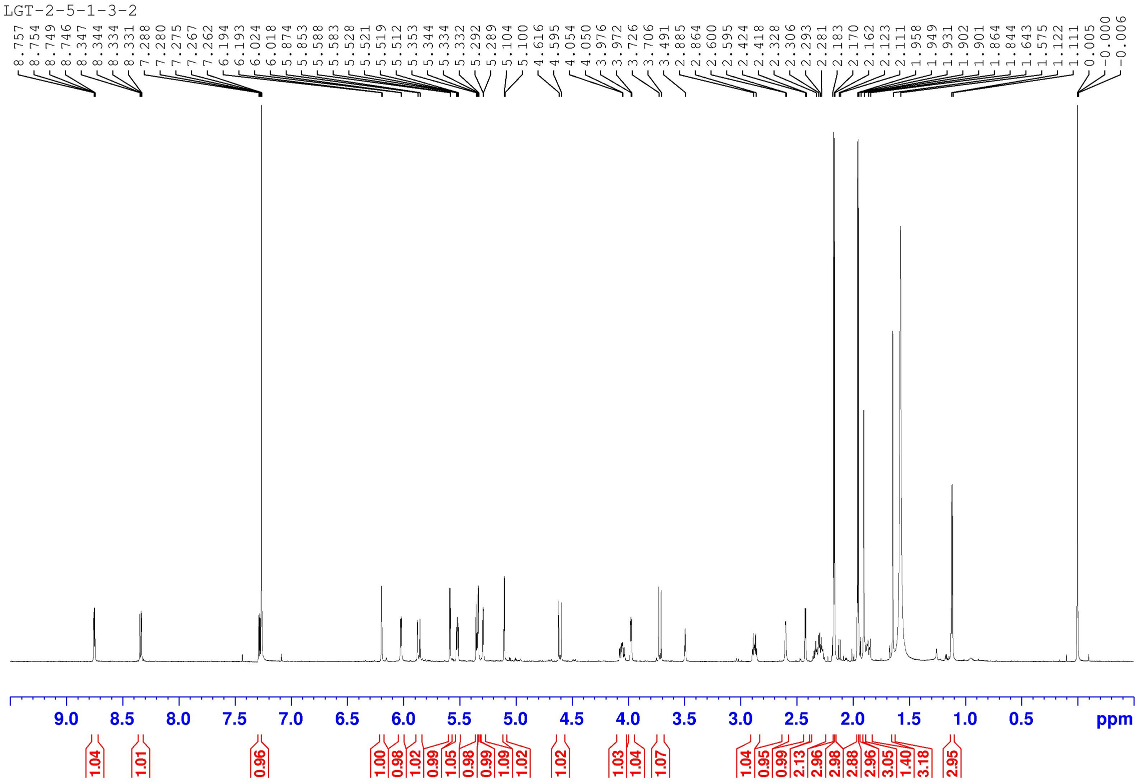

Figure S20. ^{1}H-NMR spectrum of compound **2** ($CDCl_3$, 600 MHz)

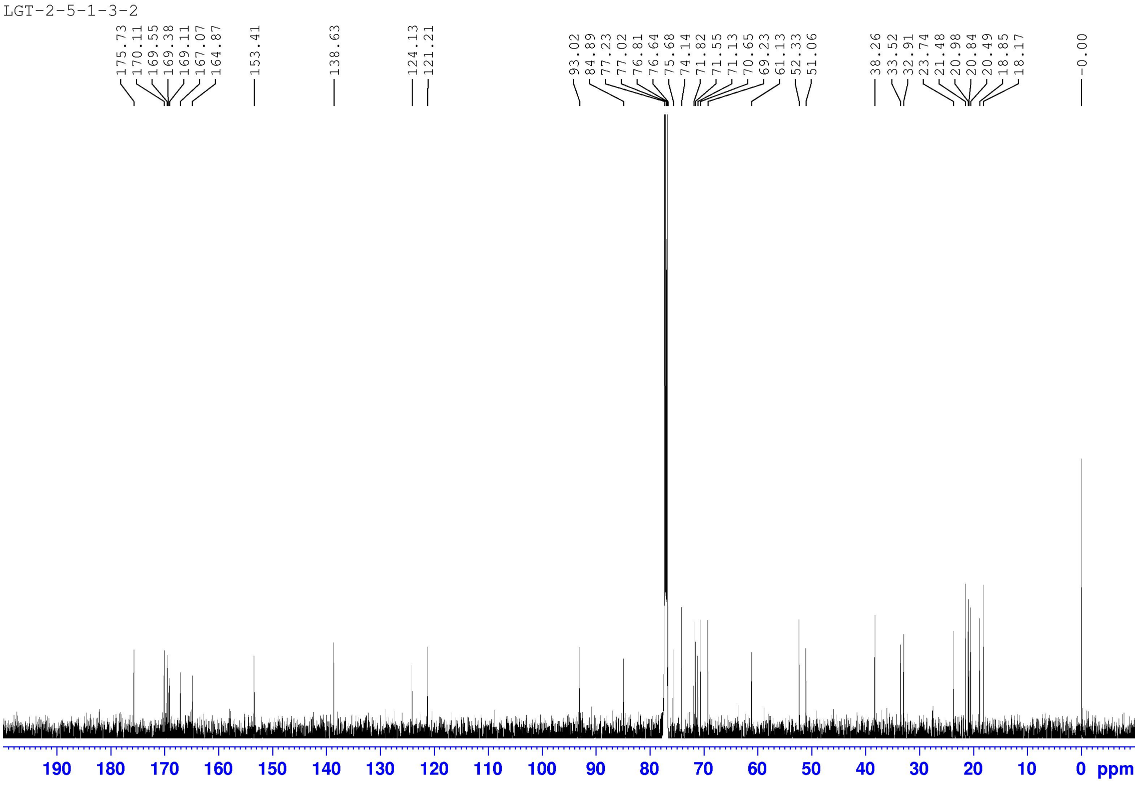

Figure S21. ^{13}C-NMR spectrum of compound **2** ($CDCl_3$, 150 MHz)

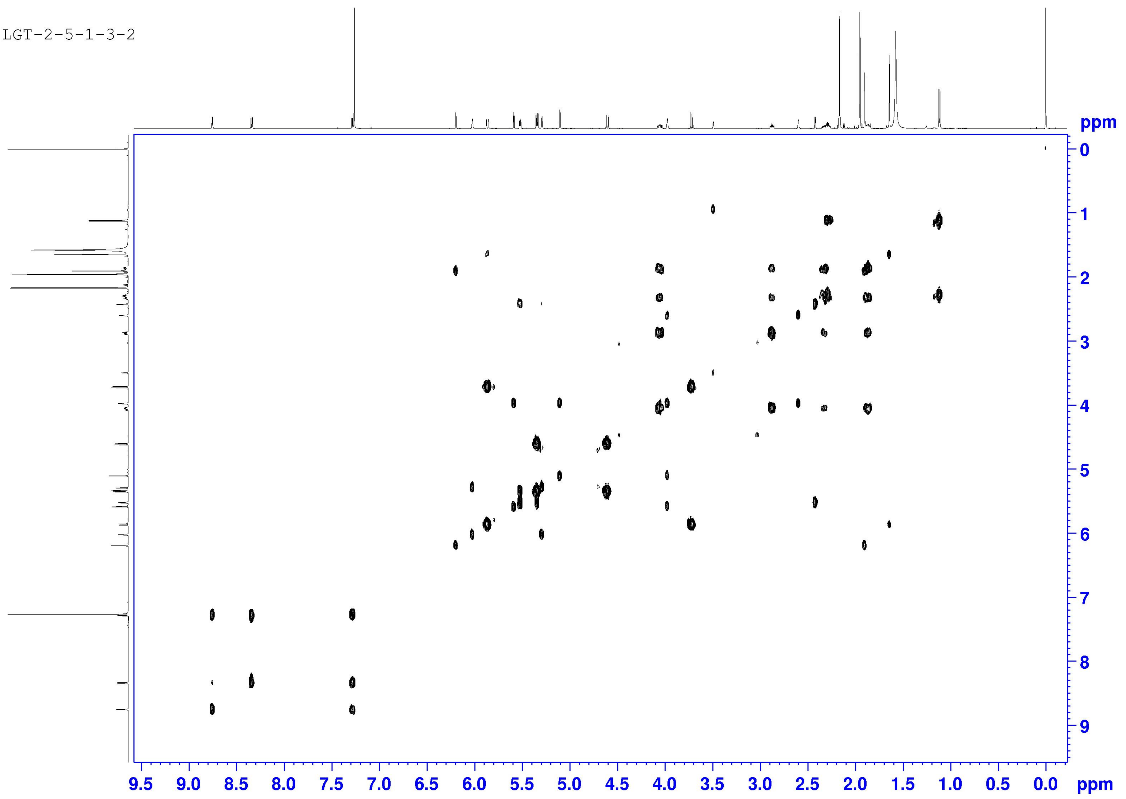

Figure S22. ^{1}H-^{1}H COSY spectrum of compound **2**

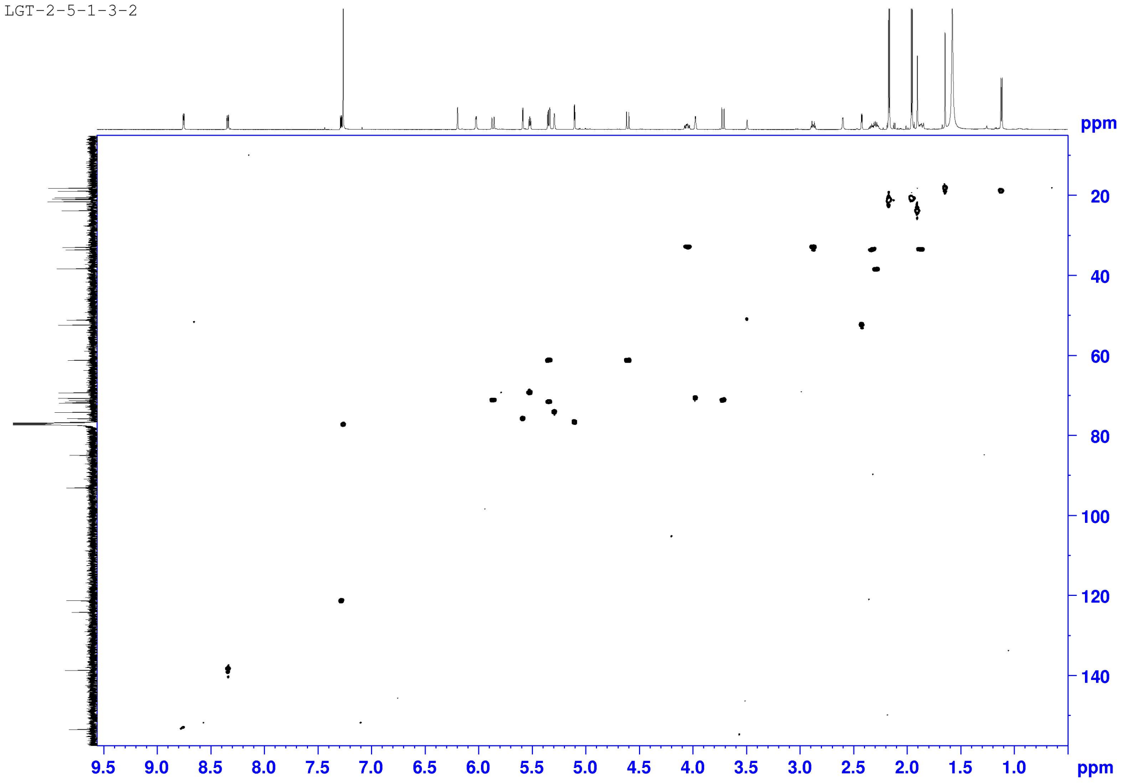

Figure S23. HSQC spectrum of compound **2**

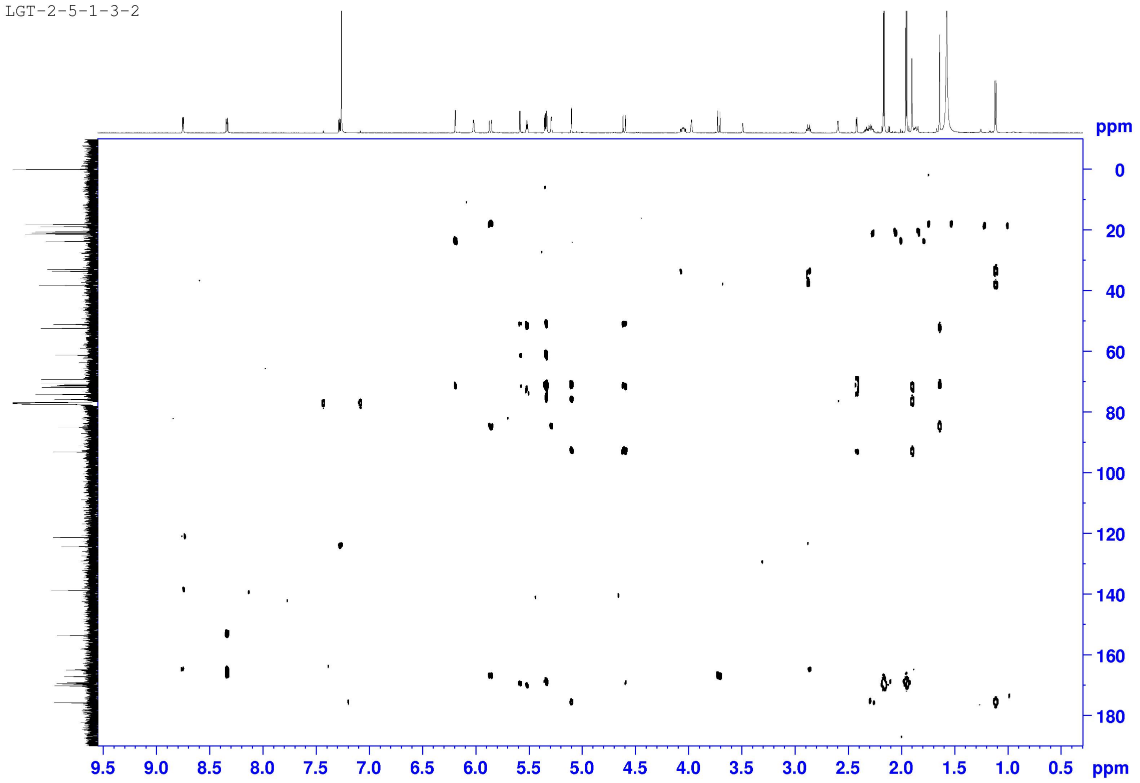

Figure S24. HMBC spectrum of compound **2**

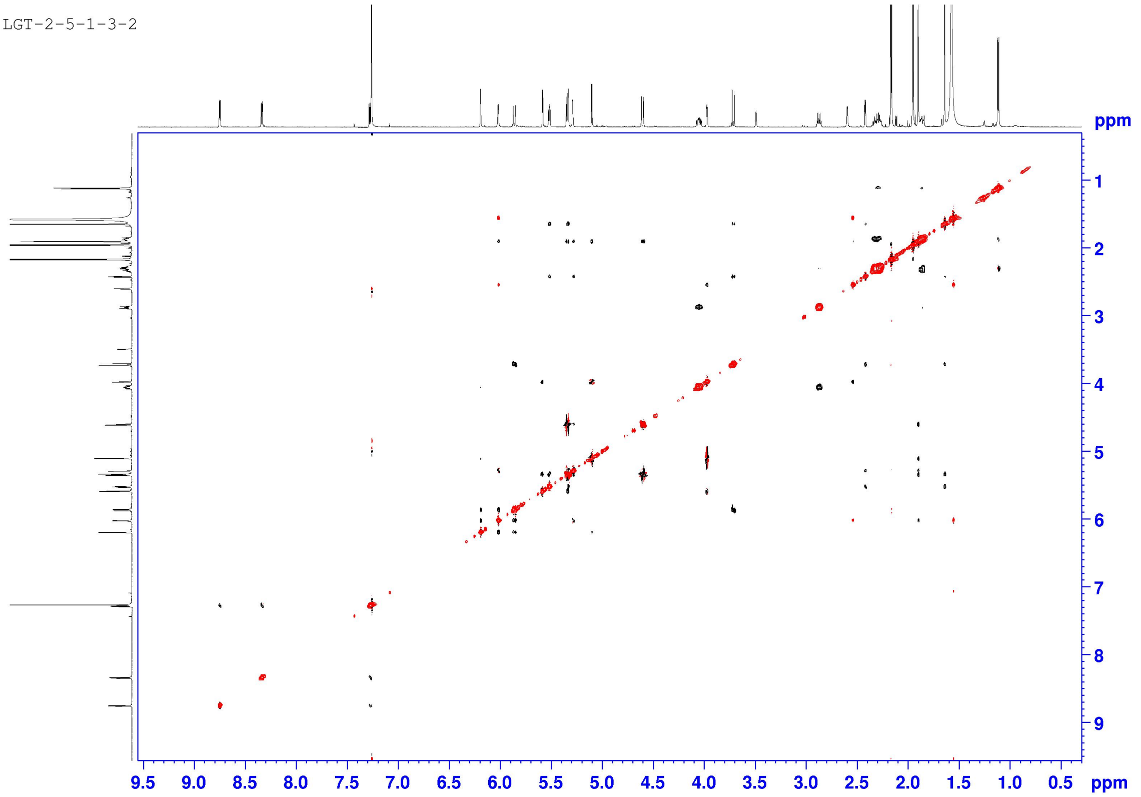

Figure S25. ROESY spectrum of compound **2**

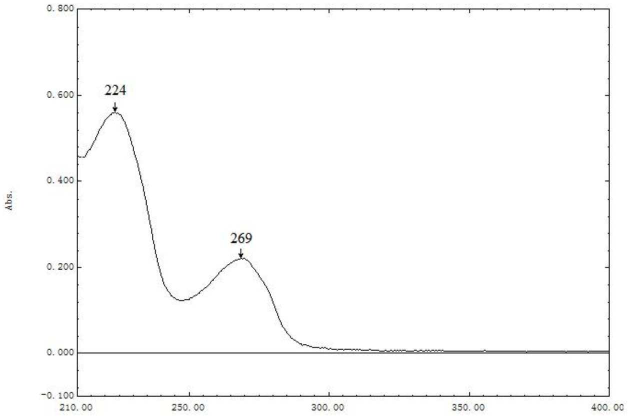

Figure S26. UV spectrum of compound **3**

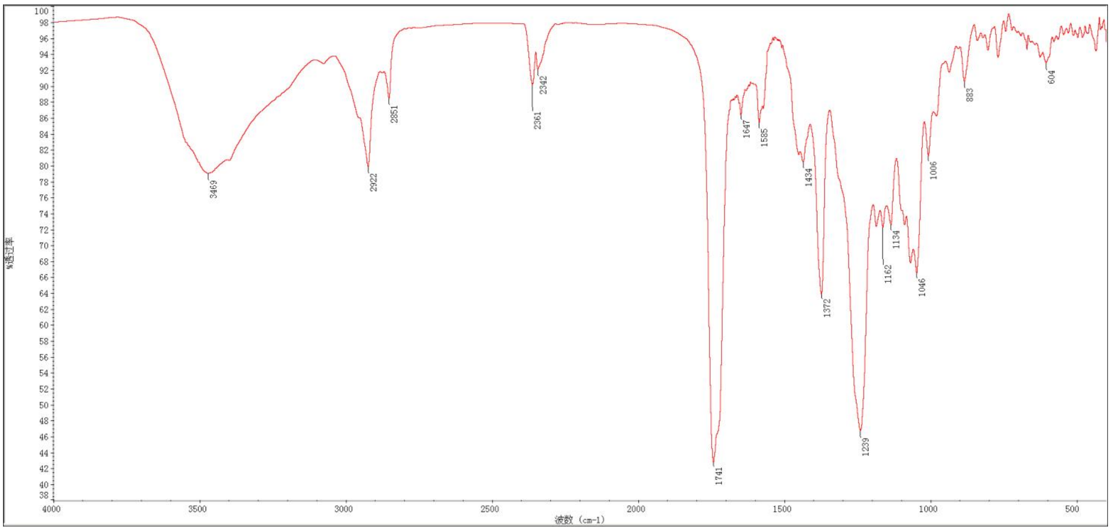

Figure S27. IR spectrum of compound **3**

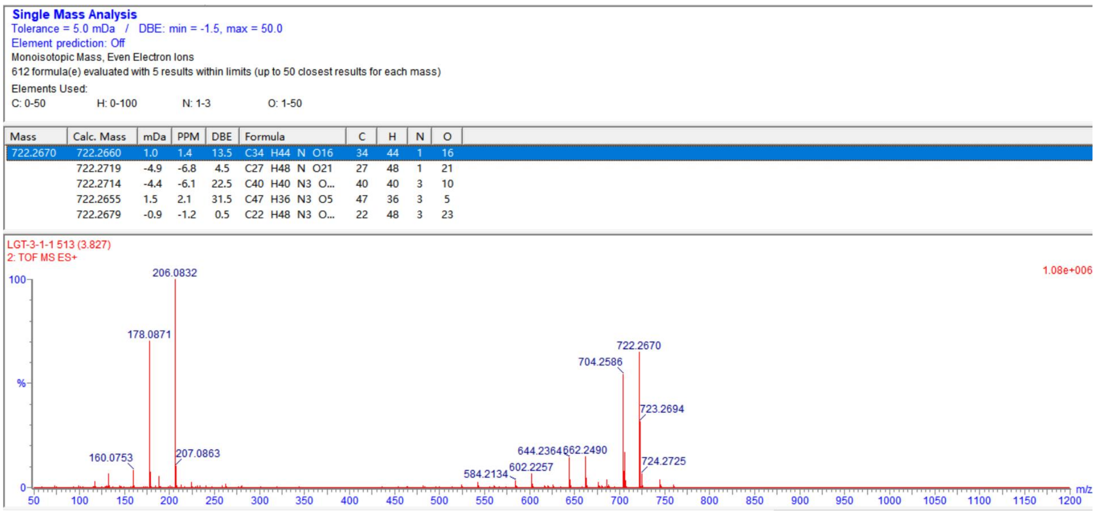

Figure S28. HRESIMS spectrum of compound **3**

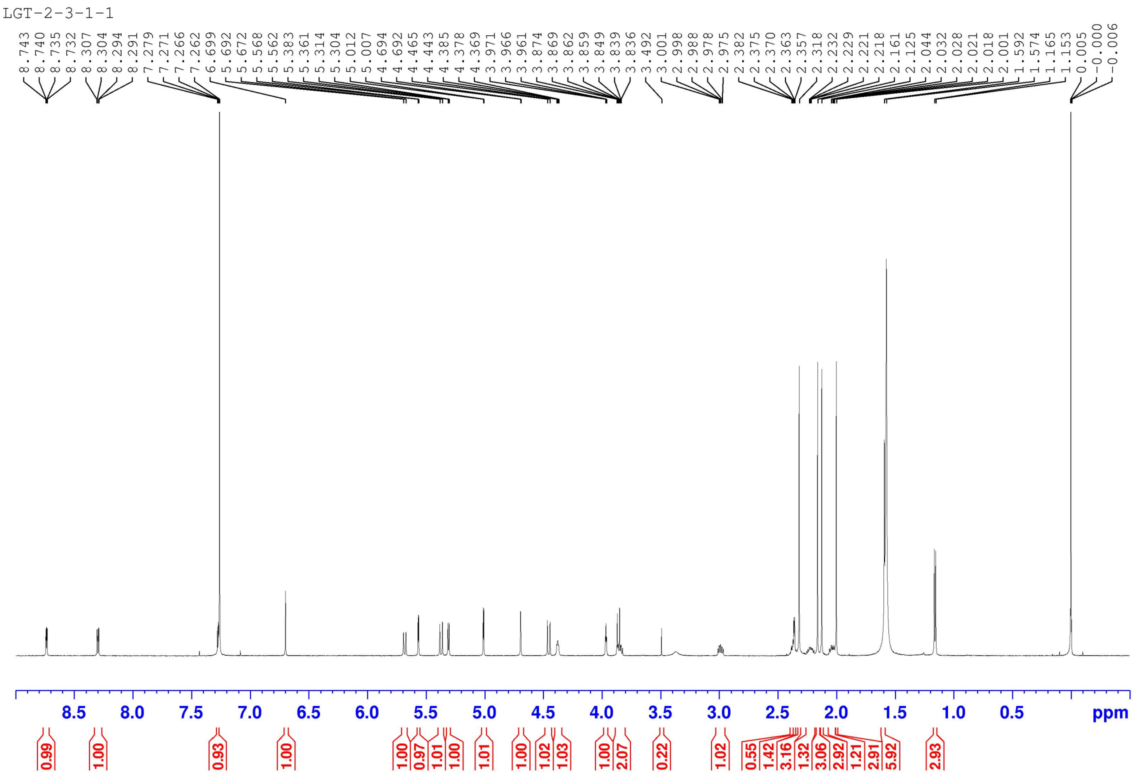

Figure S29. ^{1}H-NMR spectrum of compound **3** ($CDCl_3$, 600 MHz)

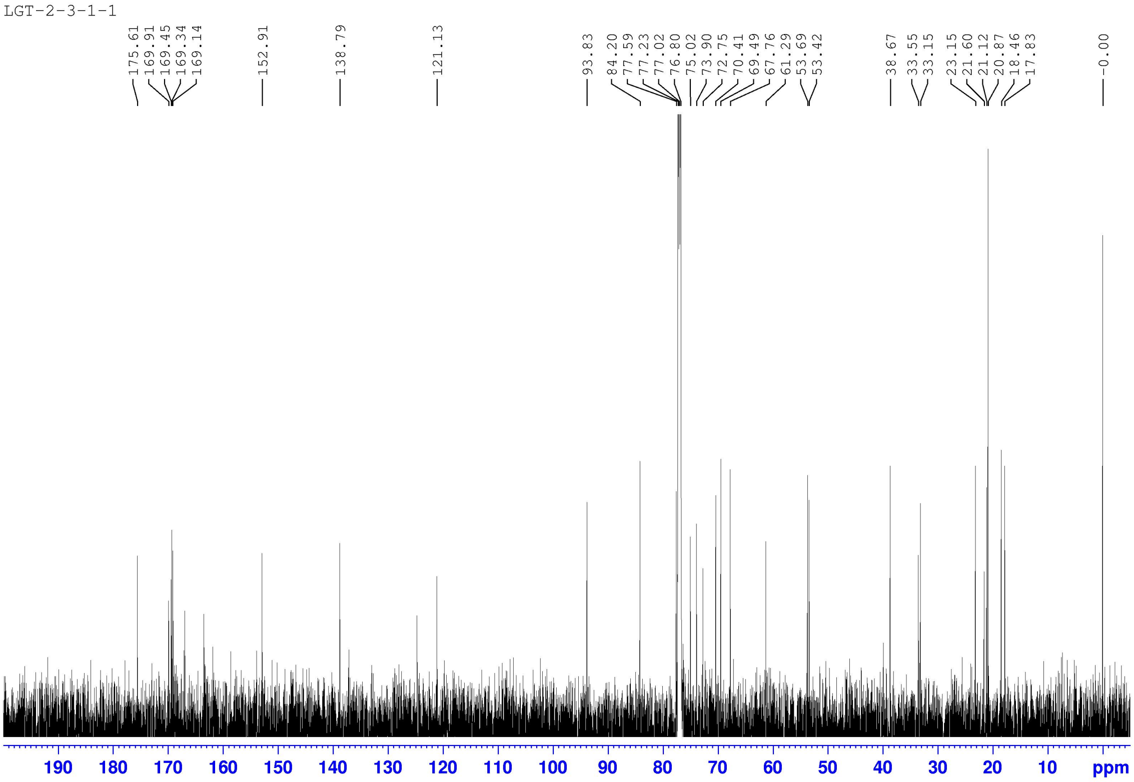

Figure S30. ^{13}C-NMR spectrum of compound **3** ($CDCl_3$, 150 MHz)

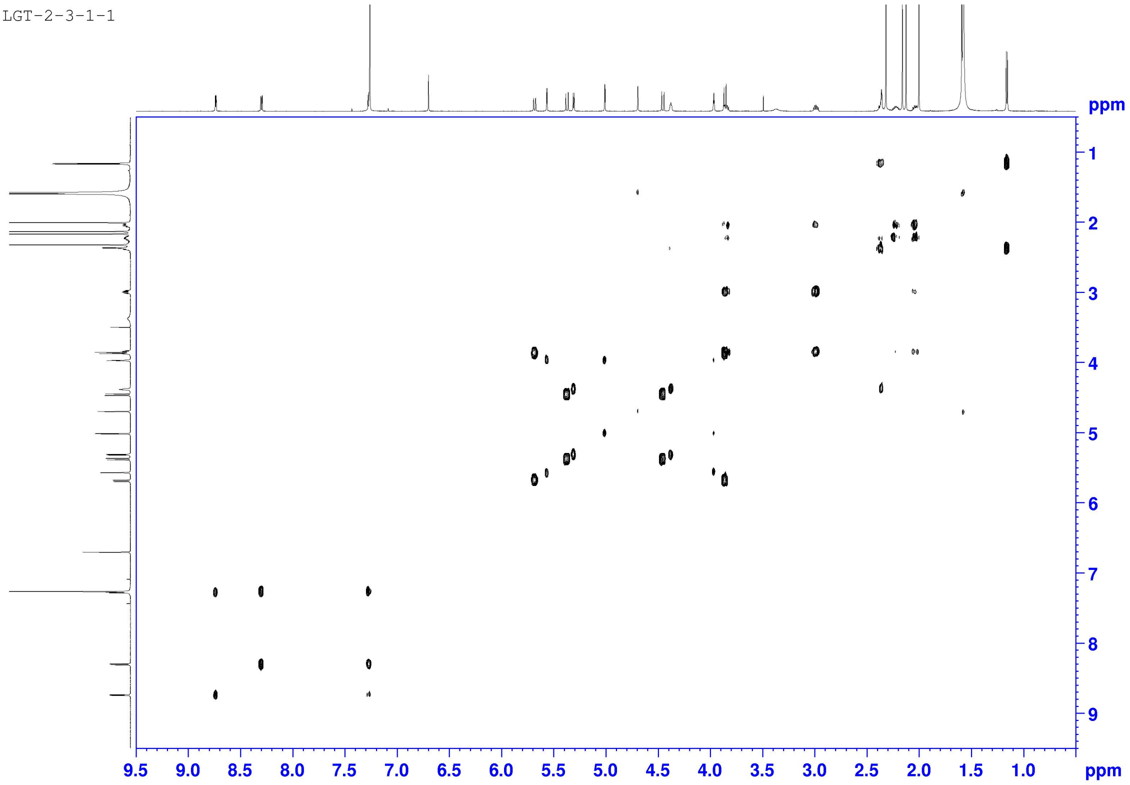

Figure S31. ^1H-^1H COSY spectrum of compound **3**

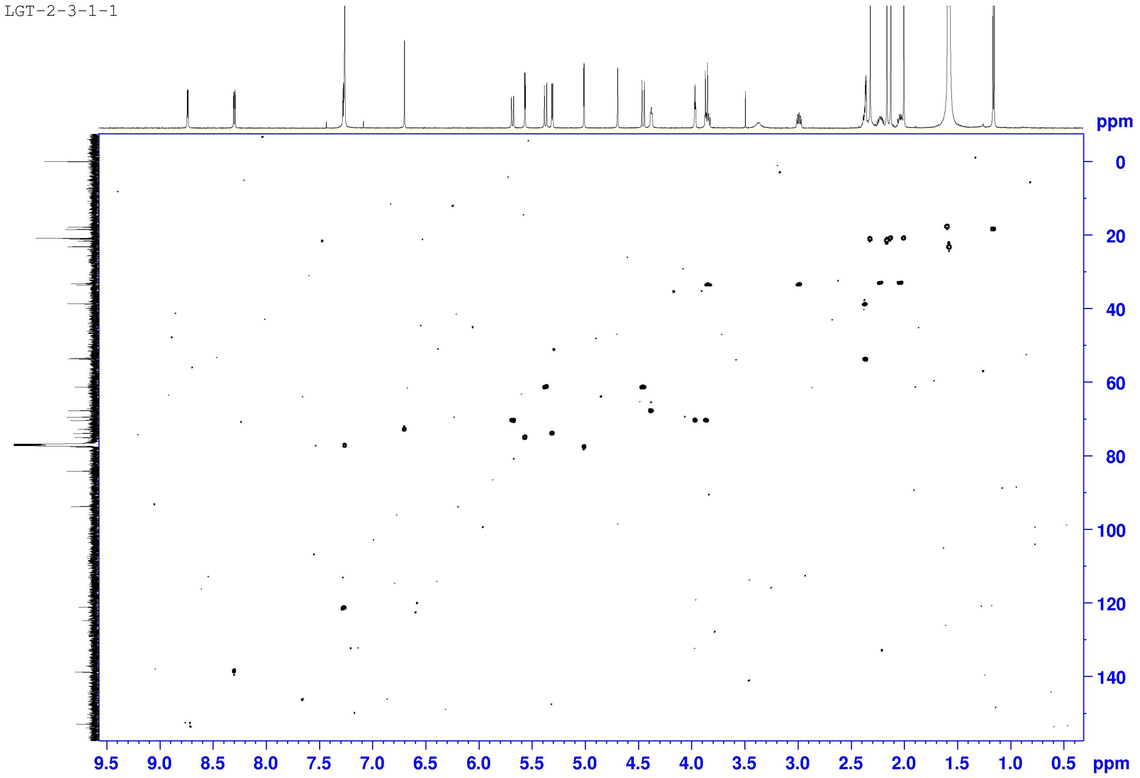

Figure S32. HSQC spectrum of compound **3**

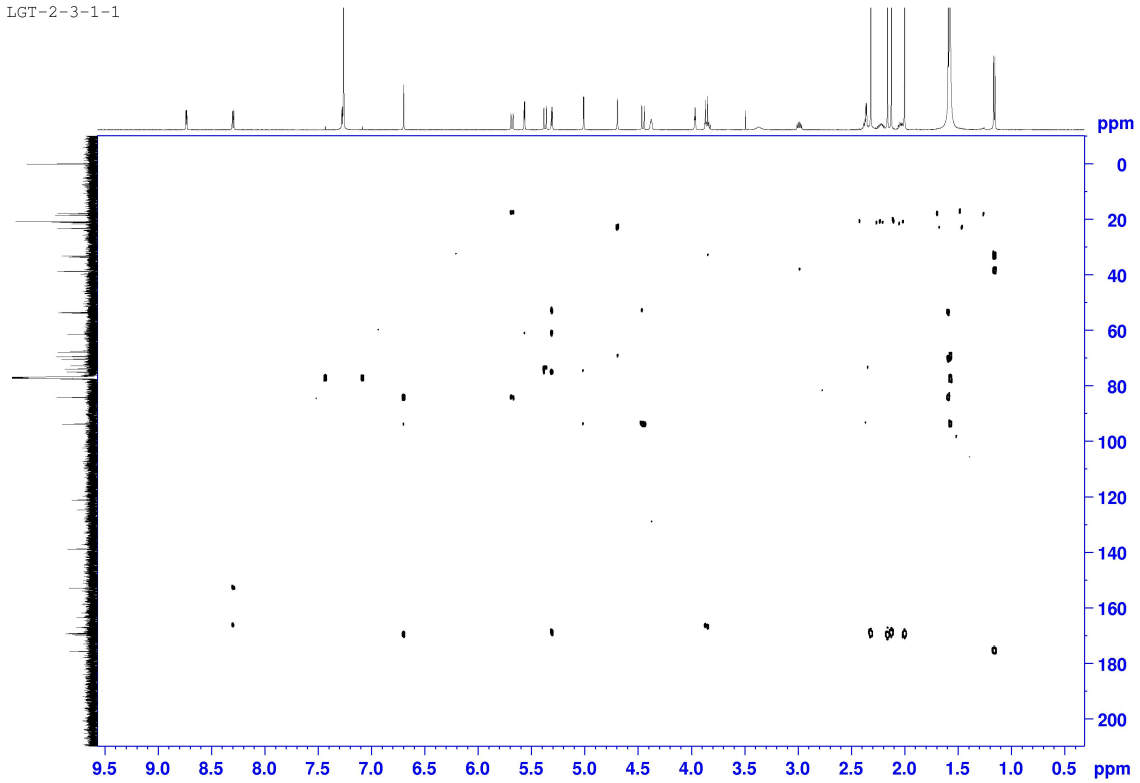

Figure S33. HMBC spectrum of compound **3**

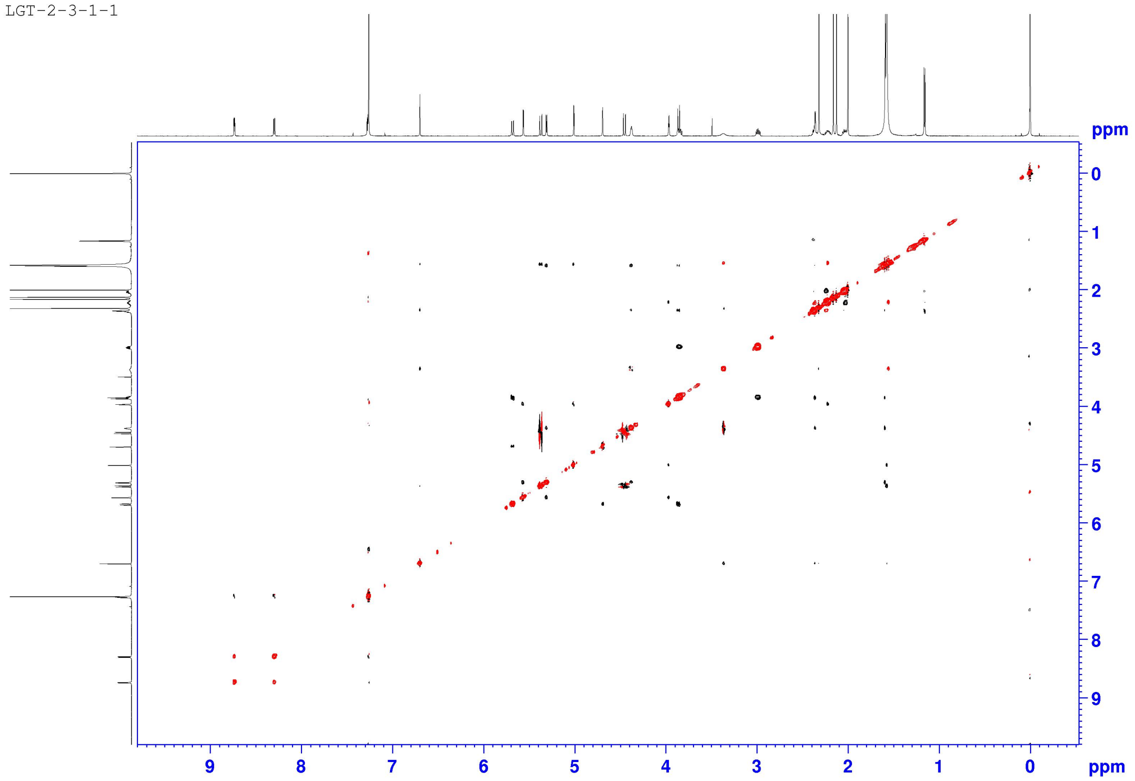

Figure S34. ROESY spectrum of compound **3**

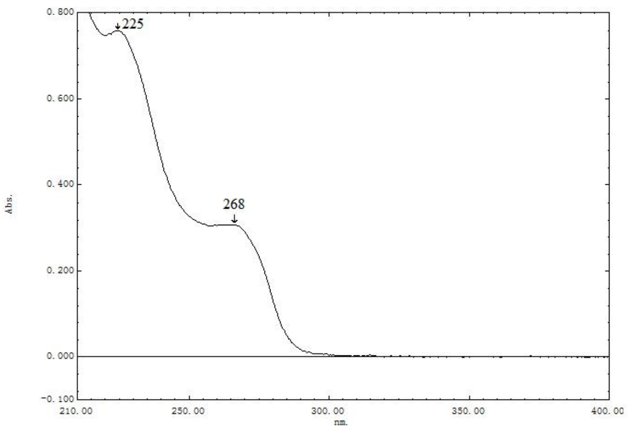

Figure S35. UV spectrum of compound **4**

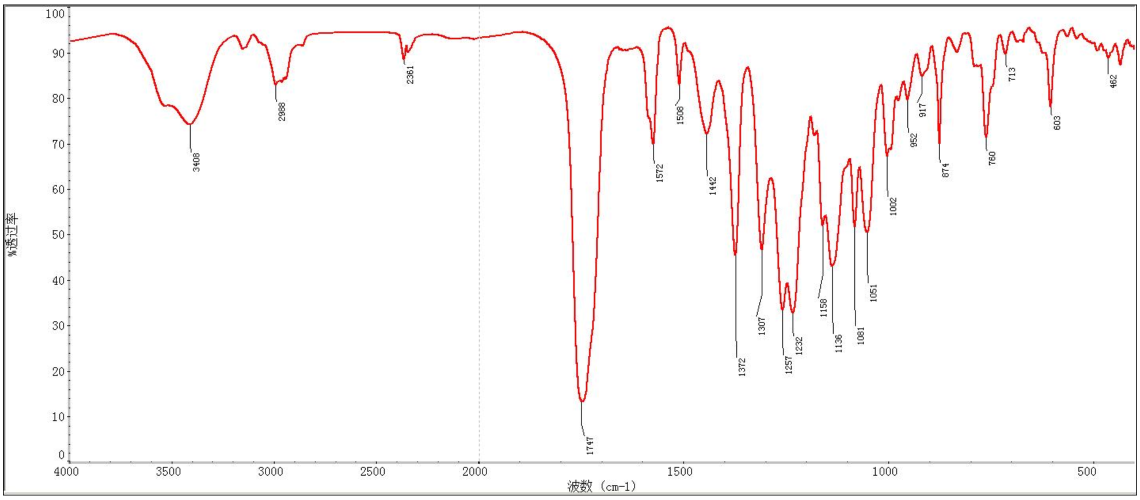

Figure S36. IR spectrum of compound **4**

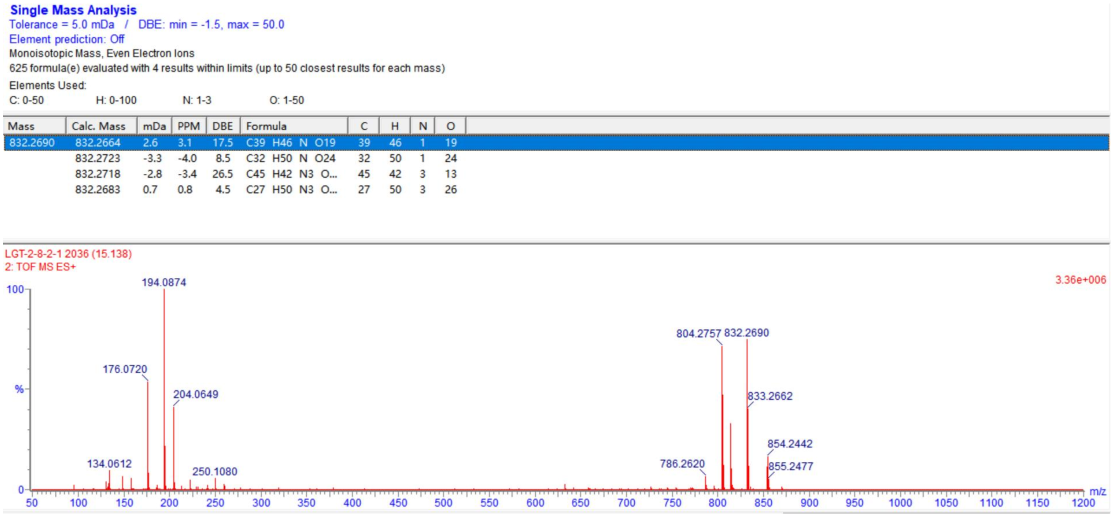

Figure S37. HRESIMS spectrum of compound **4**

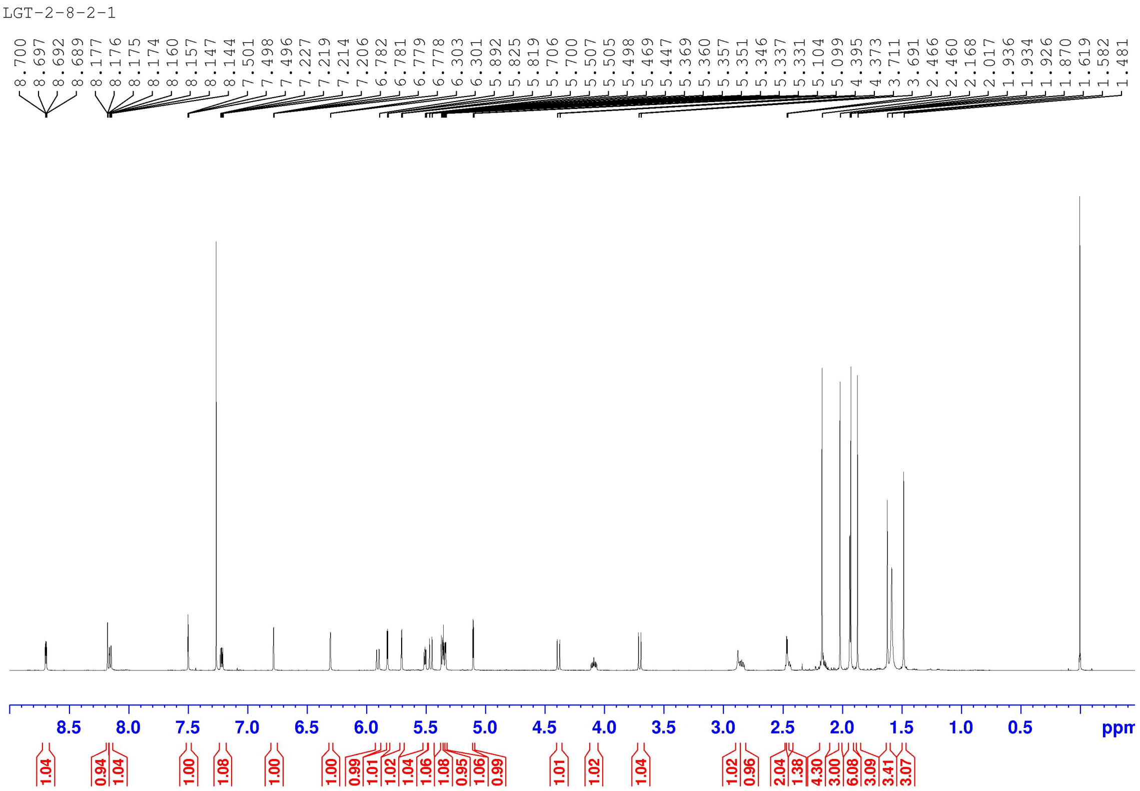

Figure S38. ^{1}H-NMR spectrum of compound **4** ($CDCl_3$, 600 MHz)

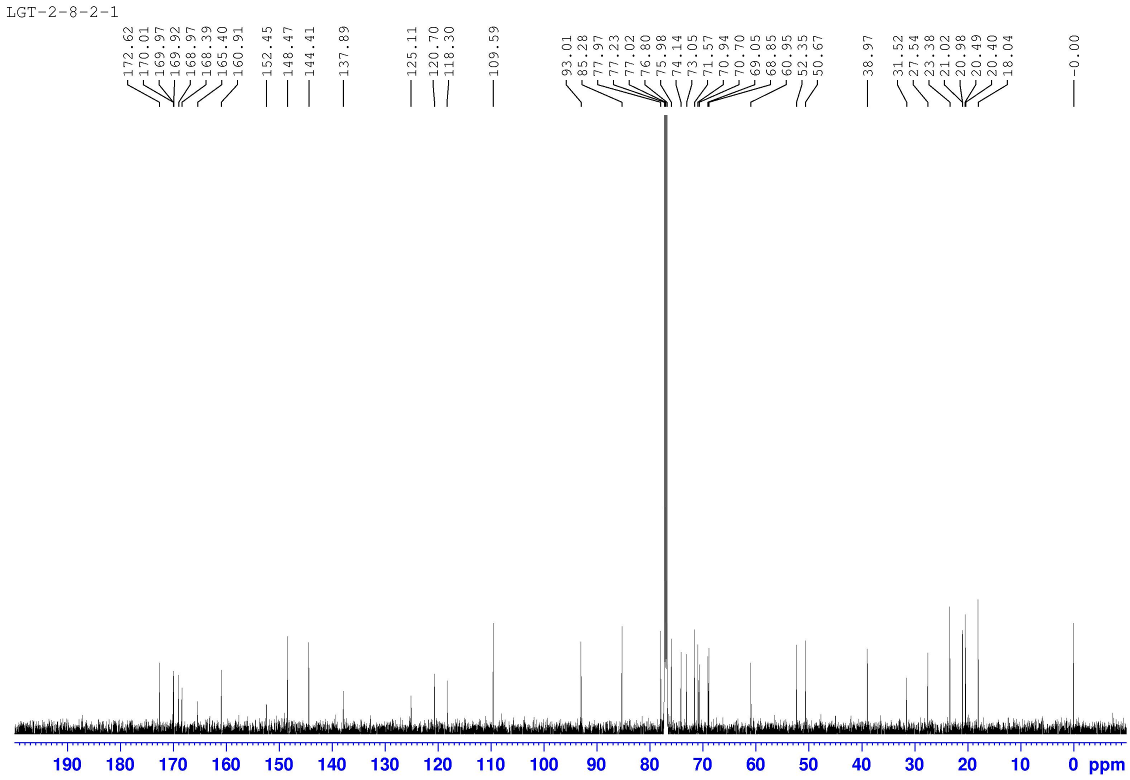

Figure S39. ^{13}C-NMR spectrum of compound **4** ($CDCl_3$, 150 MHz)

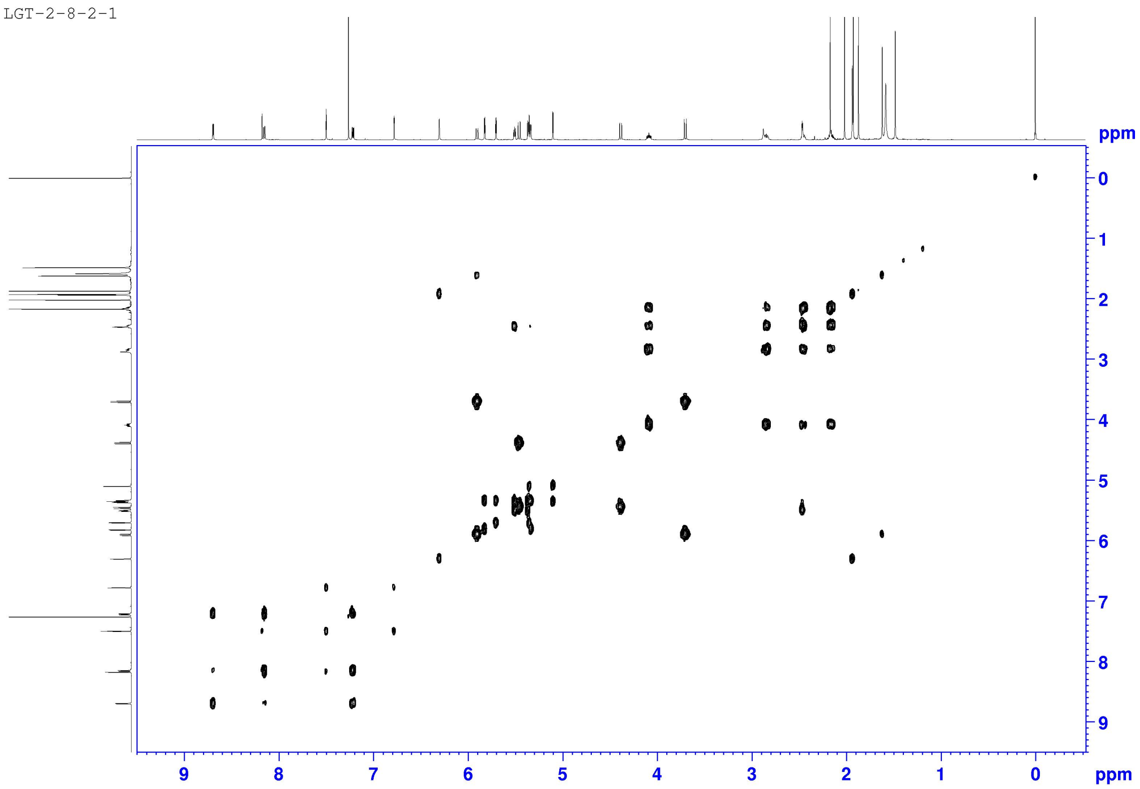

Figure S40. ^{1}H-^{1}H COSY spectrum of compound **4**

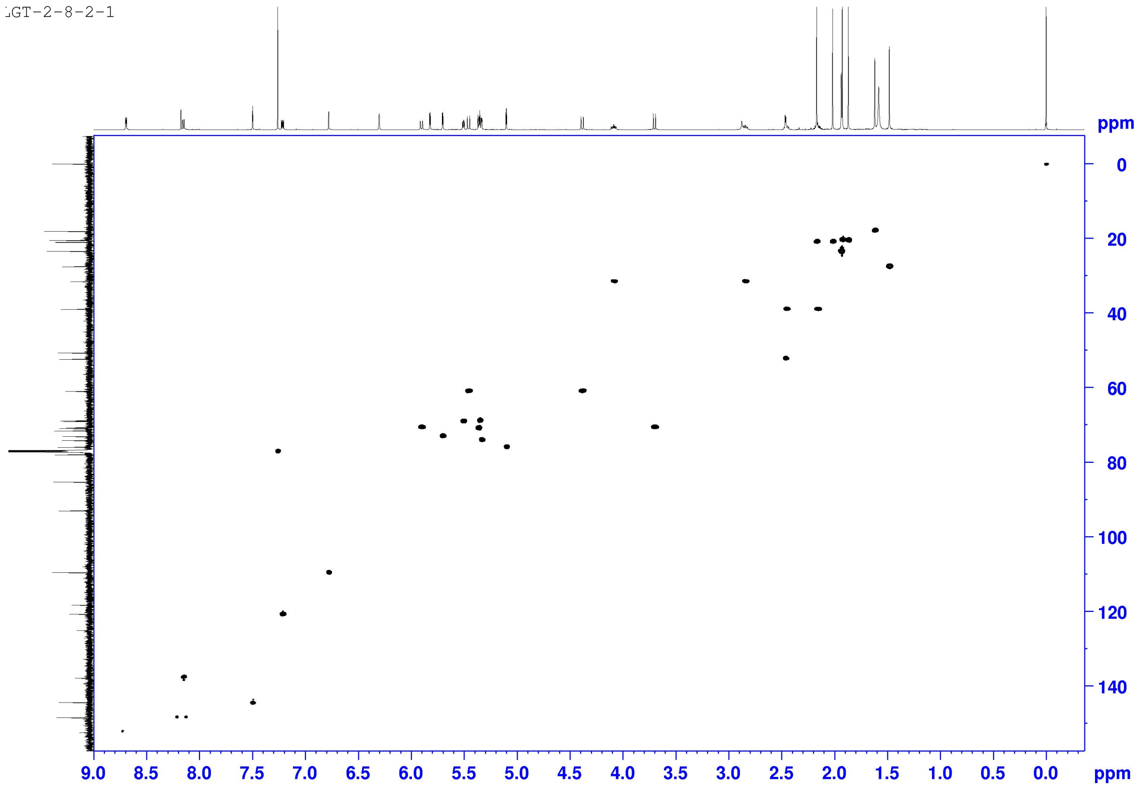

Figure S41. HSQC spectrum of compound **4**

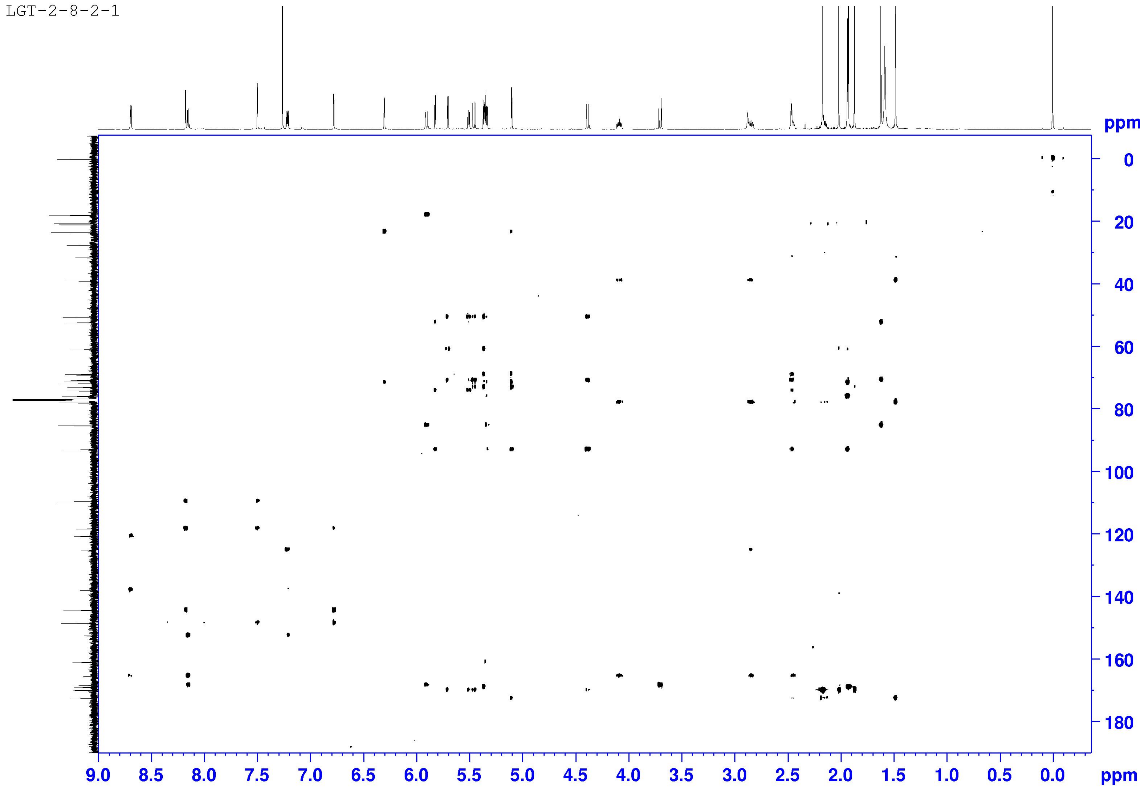

Figure S42. HMBC spectrum of compound **4**

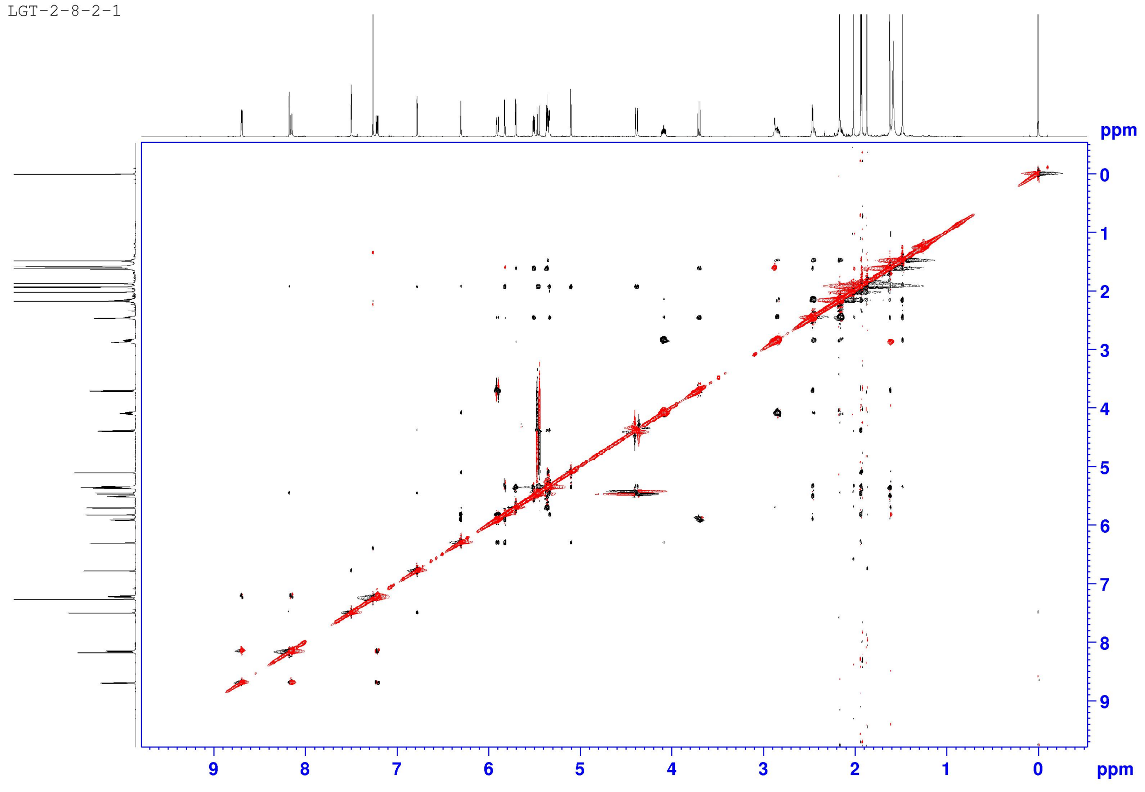

Figure S43. ROESY spectrum of compound **4**

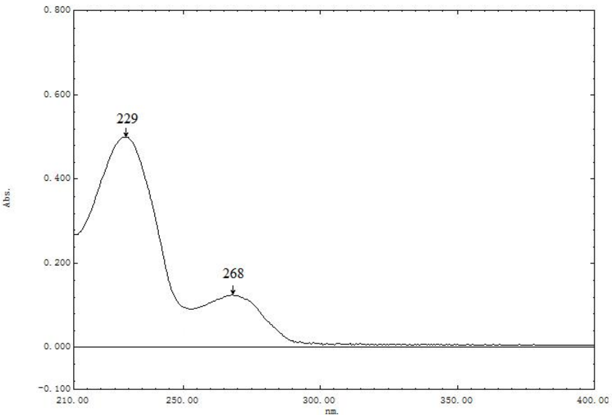

Figure S44. UV spectrum of compound **5**

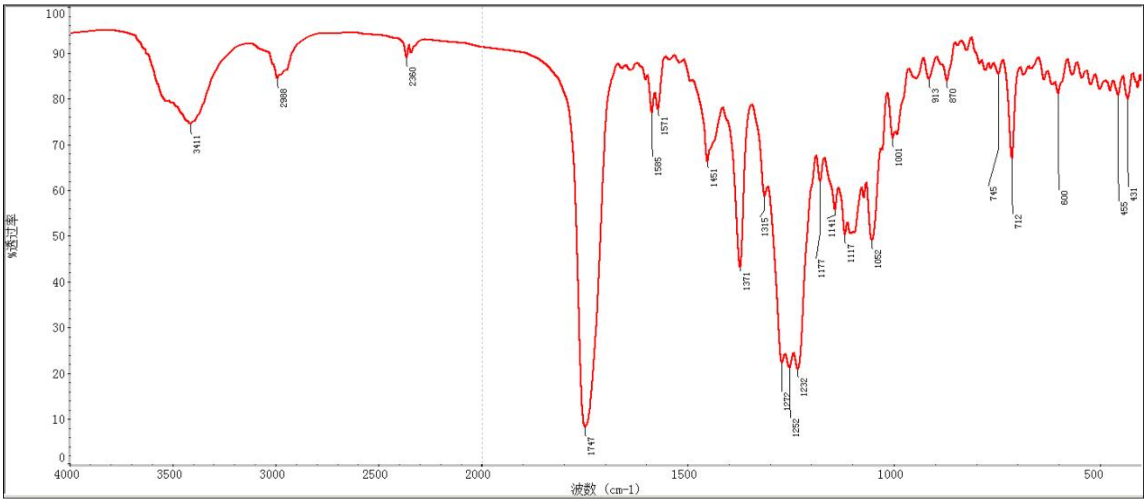

Figure S45. IR spectrum of compound **5**

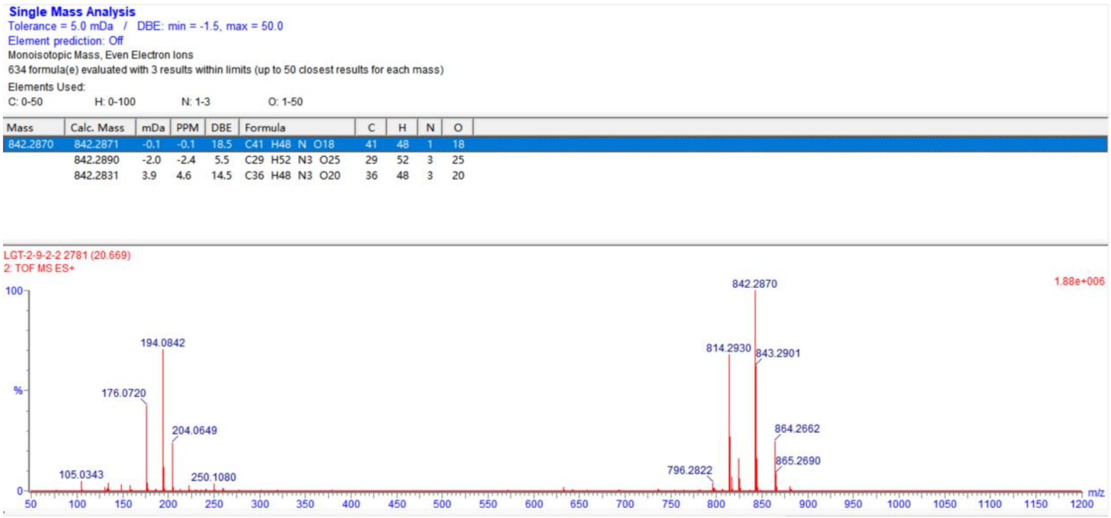

Mass	Calc. Mass	mDa	PPM	DBE	Formula	C	H	N	O
842.2870	842.2871	-0.1	-0.1	18.5	C41 H48 N O18	41	48	1	18
	842.2890	-2.0	-2.4	5.5	C29 H52 N3 O25	29	52	3	25
	842.2831	3.9	4.6	14.5	C36 H48 N3 O20	36	48	3	20

Figure S46. HRESIMS spectrum of compound **5**

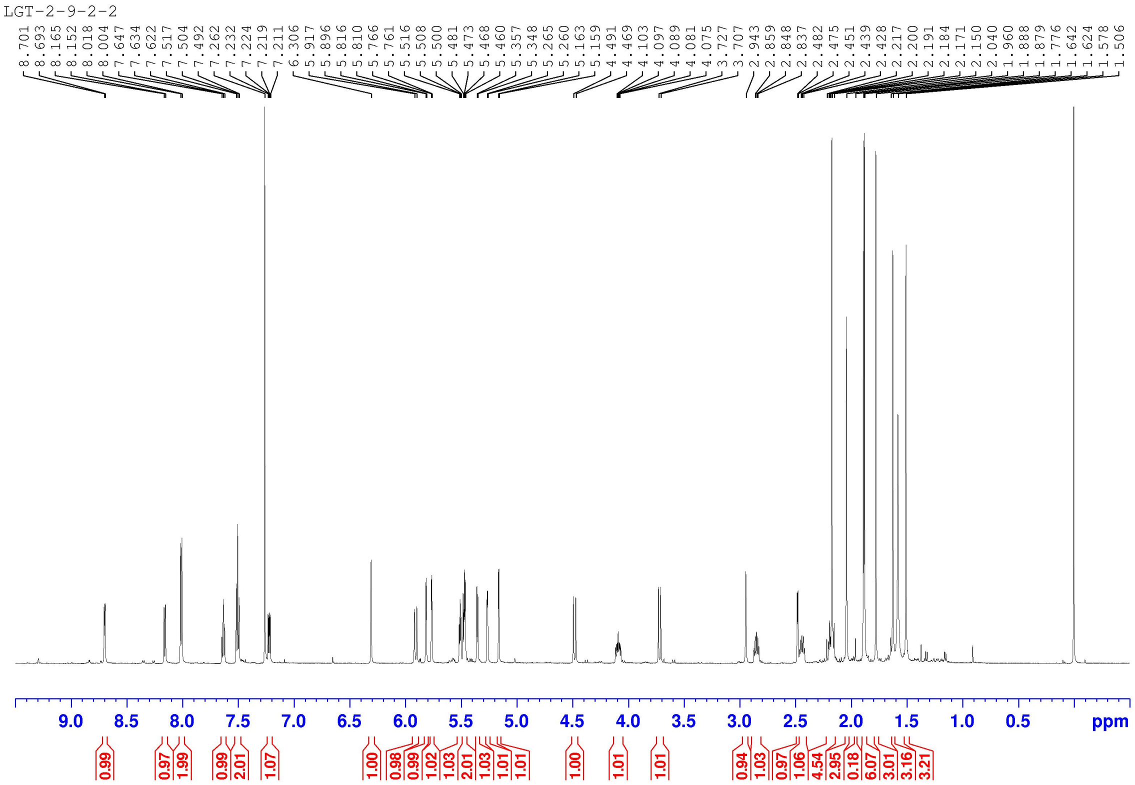

Figure S47. ^{1}H-NMR spectrum of compound **5** ($CDCl_3$, 600 MHz)

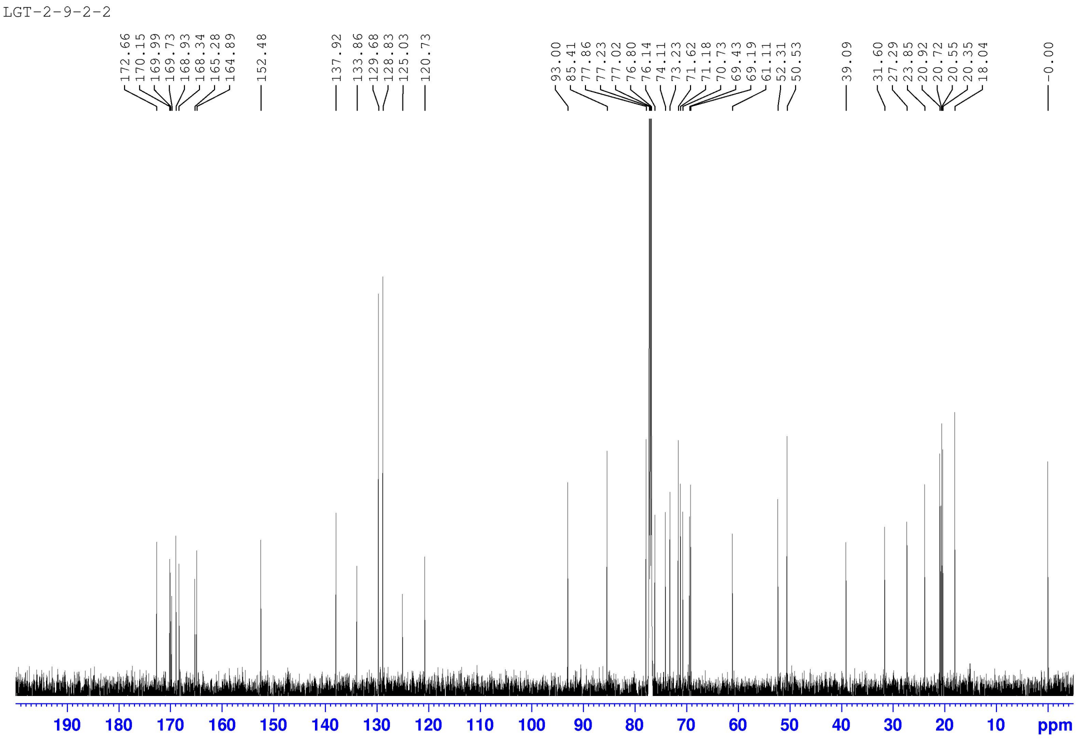

Figure S48. ^{13}C-NMR spectrum of compound **5** ($CDCl_3$, 150 MHz)

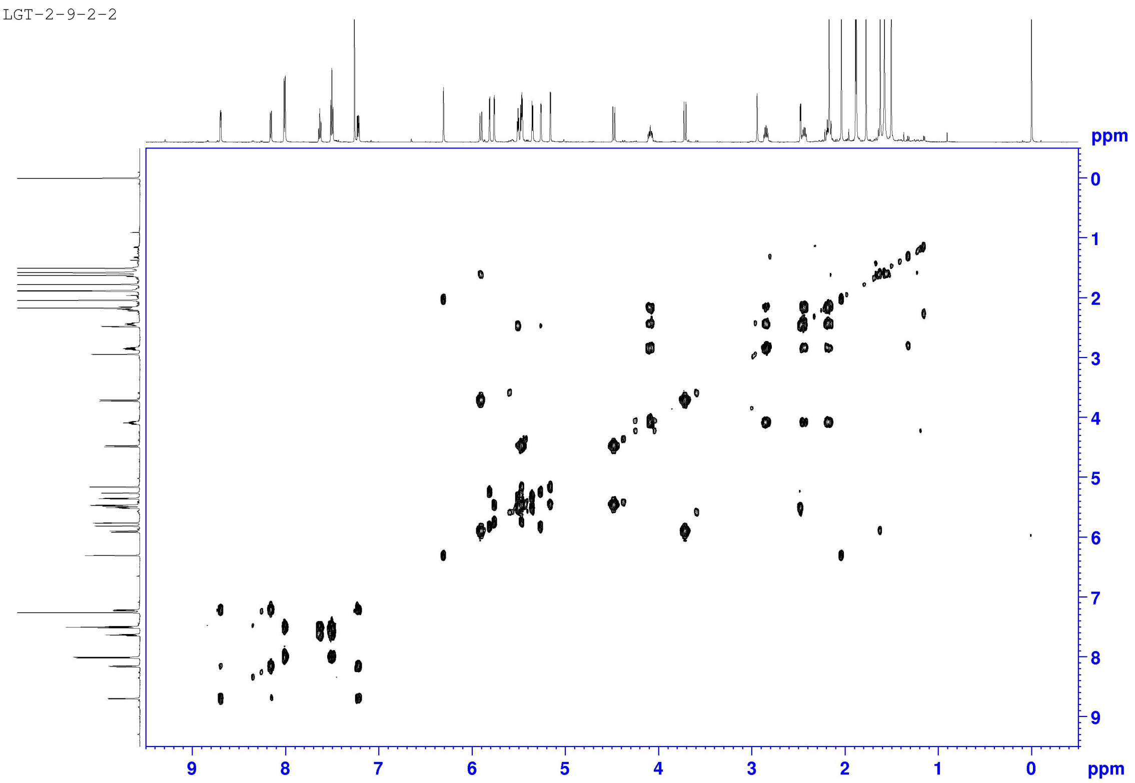

Figure S49. ^1H-^1H COSY spectrum of compound **5**

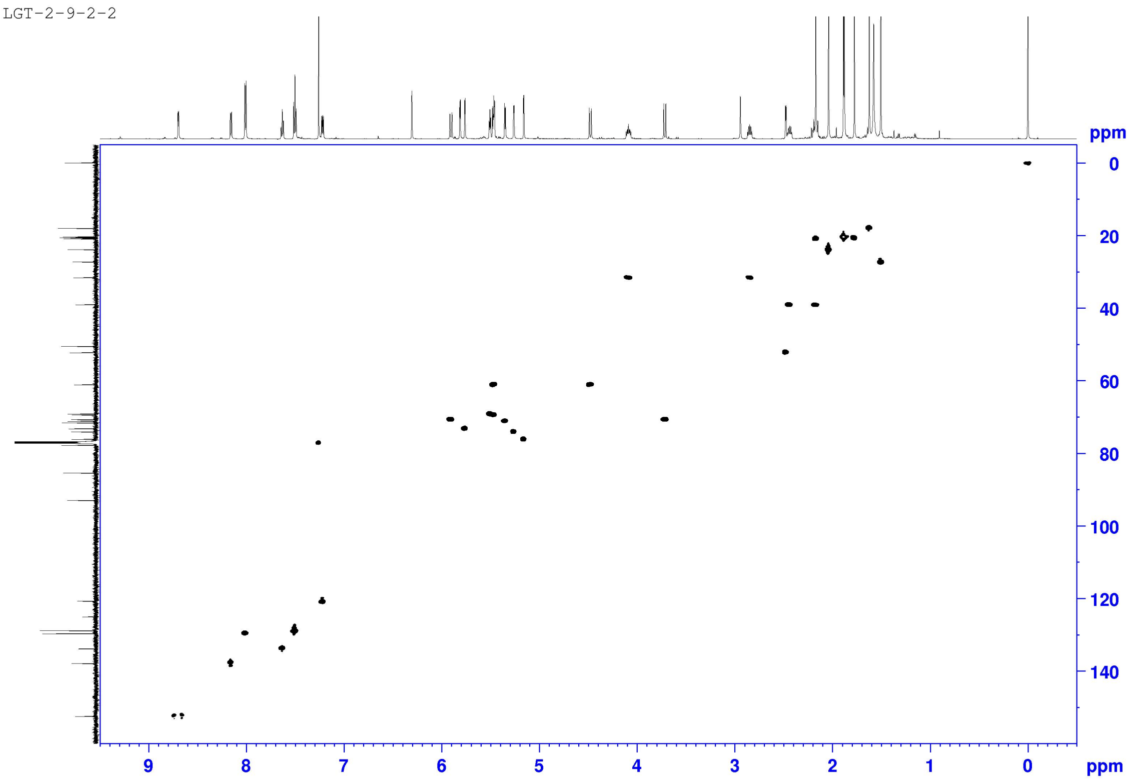

Figure S50. HSQC spectrum of compound **5**

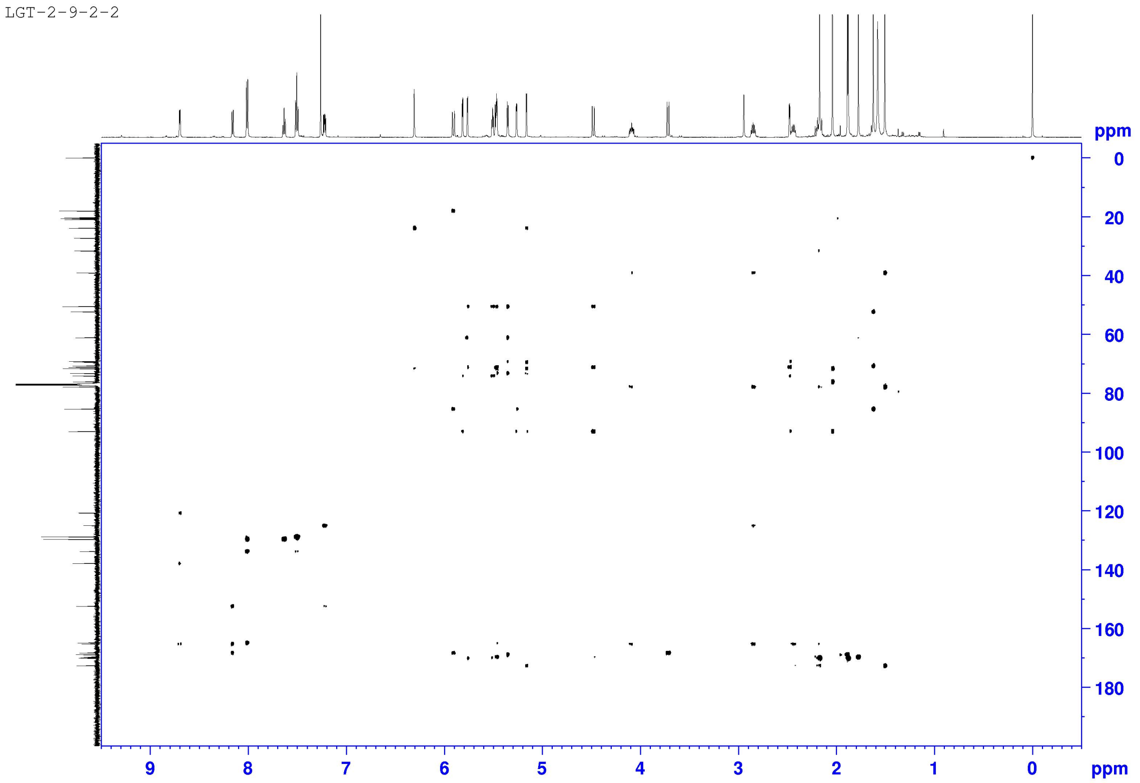

Figure S51. HMBC spectrum of compound **5**

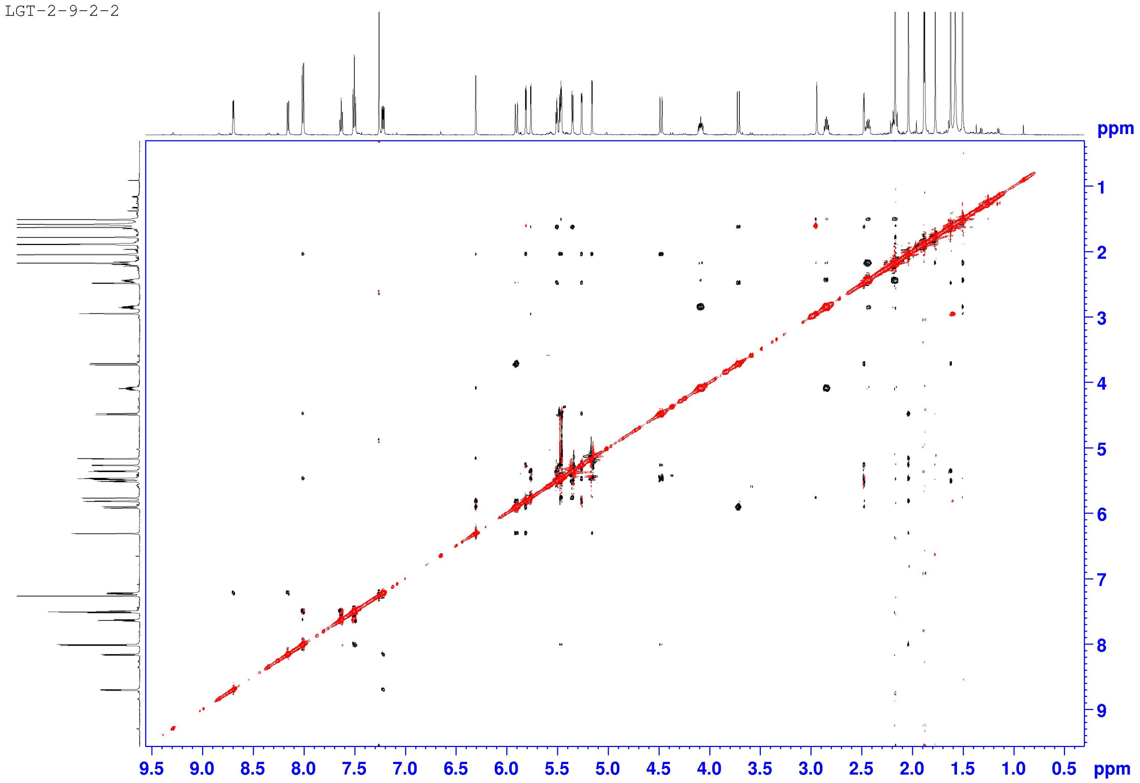

Figure S52. ROESY spectrum of compound **5**

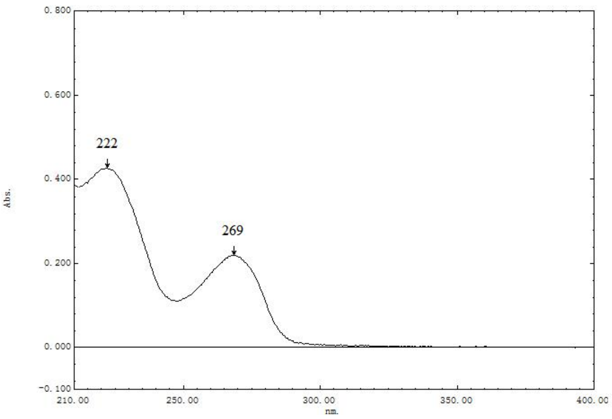

Figure S53. UV spectrum of compound **6**

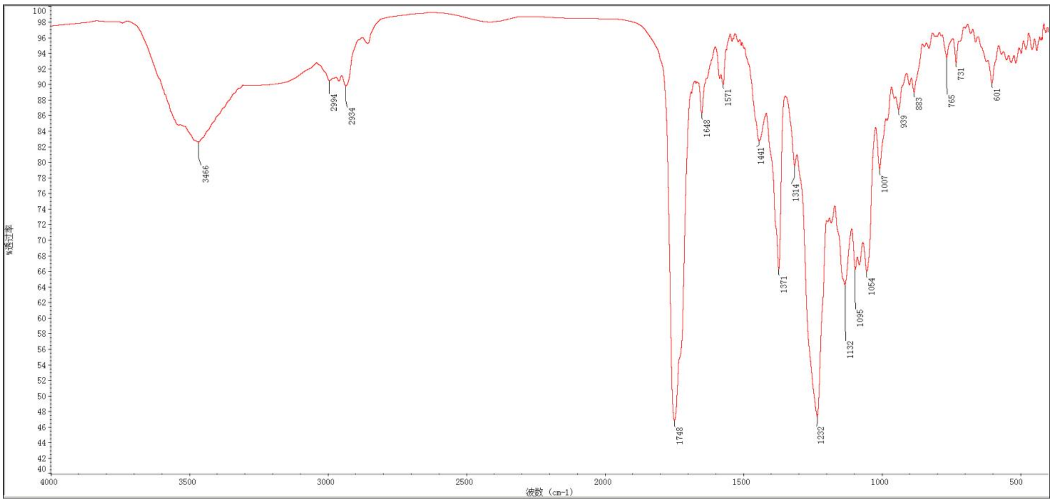

Figure S54. IR spectrum of compound **6**

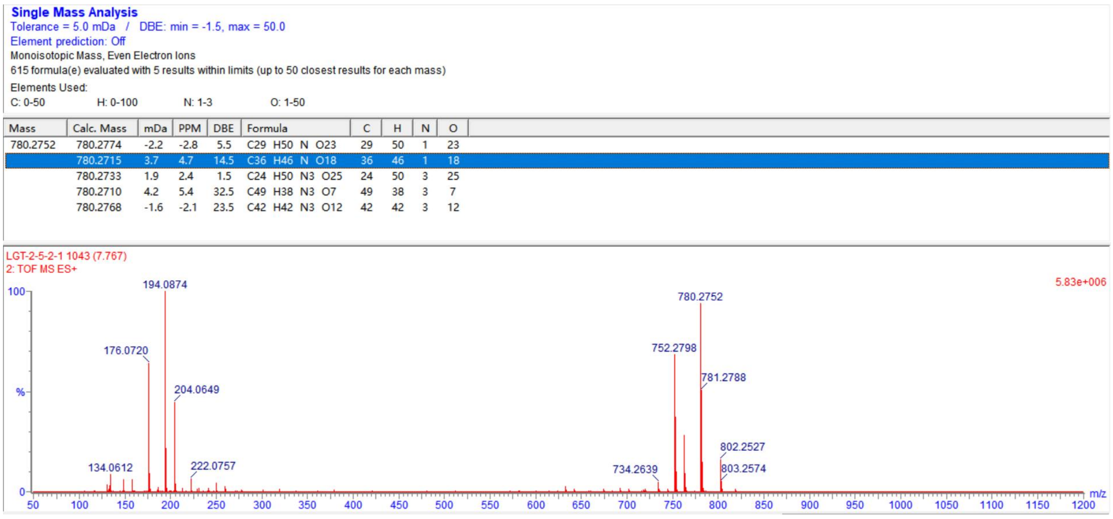

Single Mass Analysis
Tolerance = 5.0 mDa / DBE: min = -1.5, max = 50.0
Element prediction: Off
Monoisotopic Mass, Even Electron Ions
615 formula(e) evaluated with 5 results within limits (up to 50 closest results for each mass)
Elements Used:
C: 0-50 H: 0-100 N: 1-3 O: 1-50

Mass	Calc. Mass	mDa	PPM	DBE	Formula	C	H	N	O
780.2752	780.2774	-2.2	-2.8	5.5	C29 H50 N O23	29	50	1	23
	780.2715	3.7	4.7	14.5	C36 H46 N O18	36	46	1	18
	780.2733	1.9	2.4	1.5	C24 H50 N3 O25	24	50	3	25
	780.2710	4.2	5.4	32.5	C49 H38 N3 O7	49	38	3	7
	780.2768	-1.6	-2.1	23.5	C42 H42 N3 O12	42	42	3	12

Figure S55. HRESIMS spectrum of compound **6**

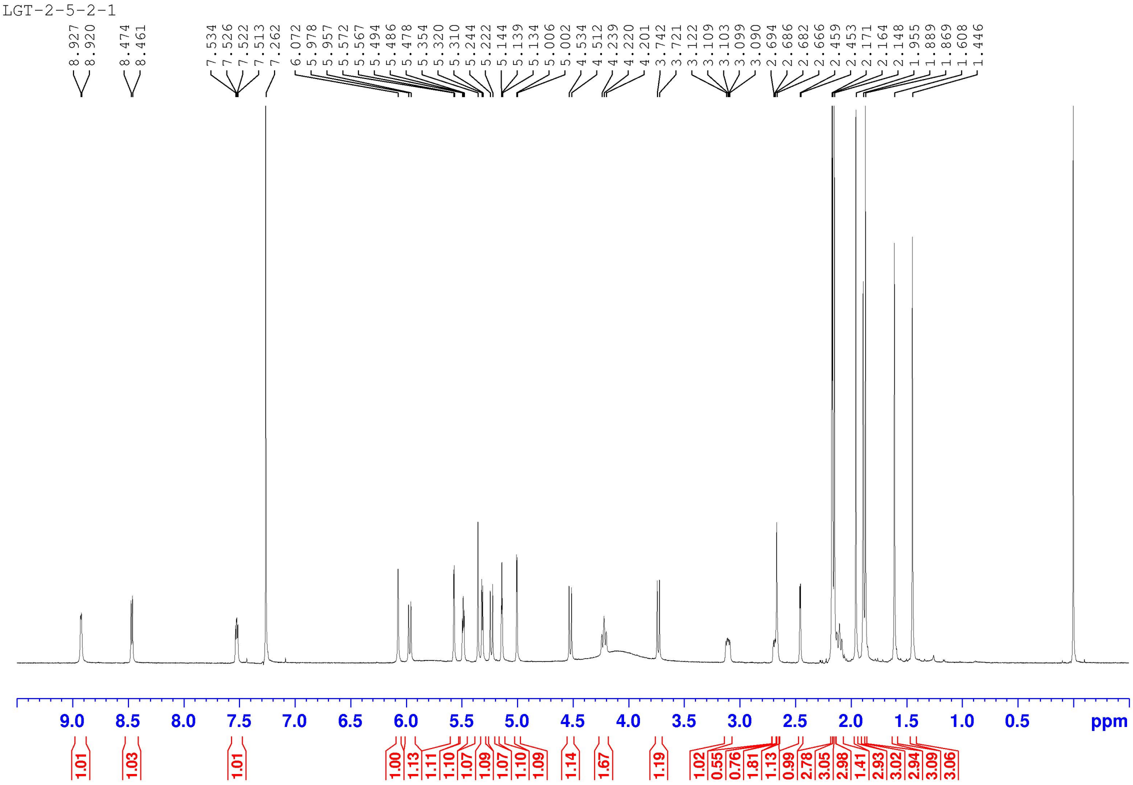

Figure S56. ^{1}H-NMR spectrum of compound **6** ($CDCl_3$, 600 MHz)

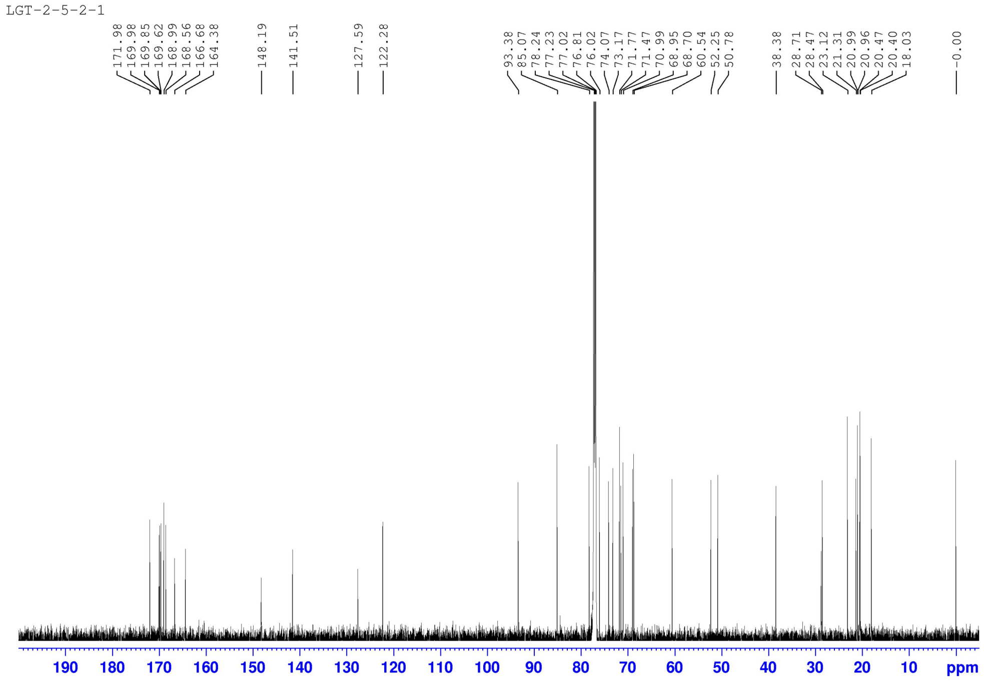

Figure S57. ^{13}C-NMR spectrum of compound **6** ($CDCl_3$, 150 MHz)

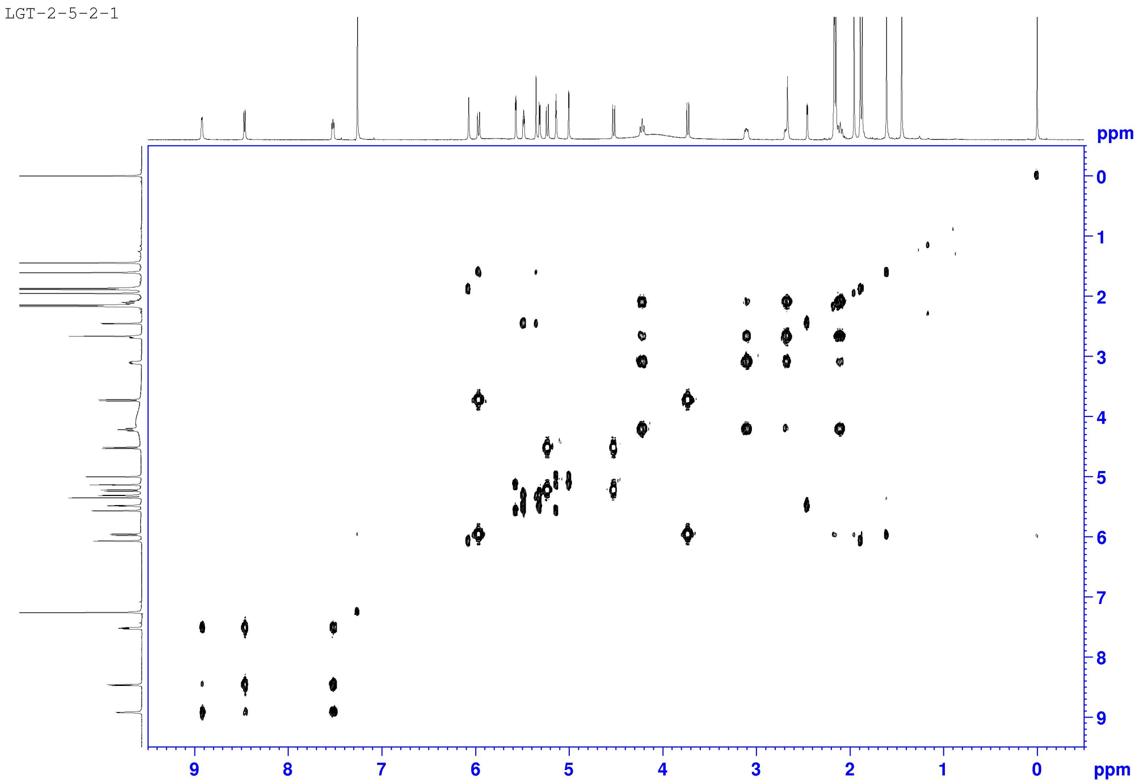

Figure S58. ^{1}H-^{1}H COSY spectrum of compound **6**

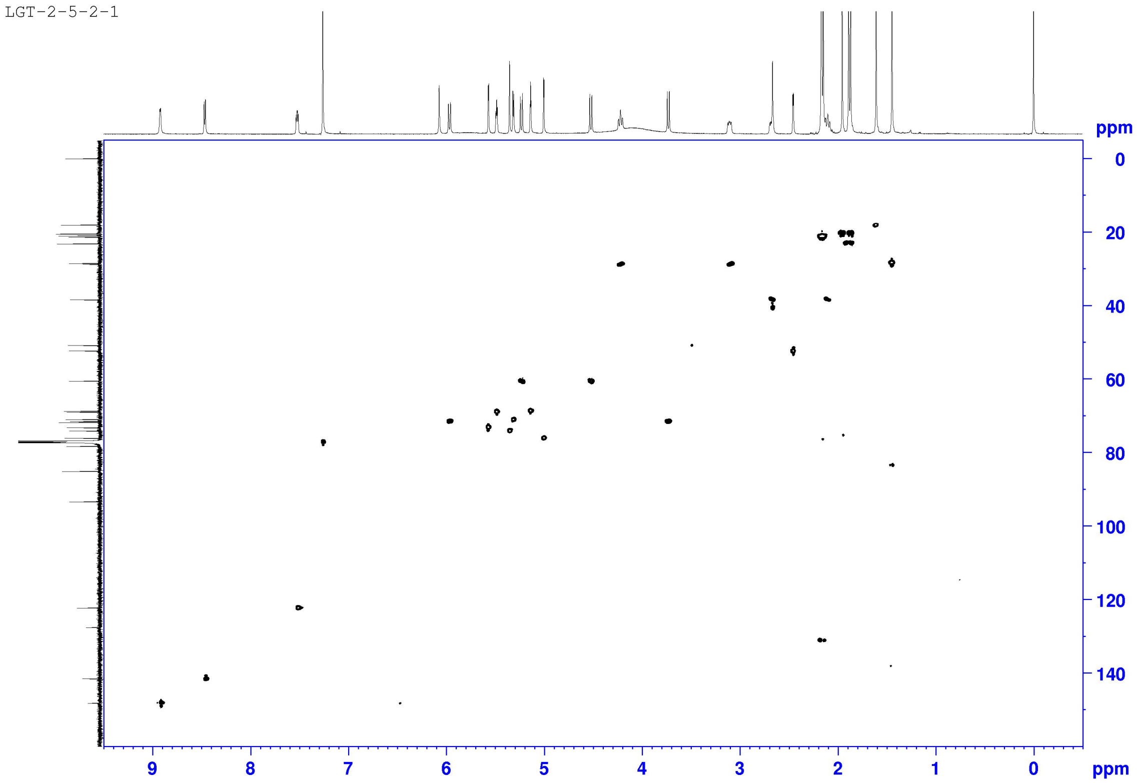

Figure S59. HSQC spectrum of compound **6**

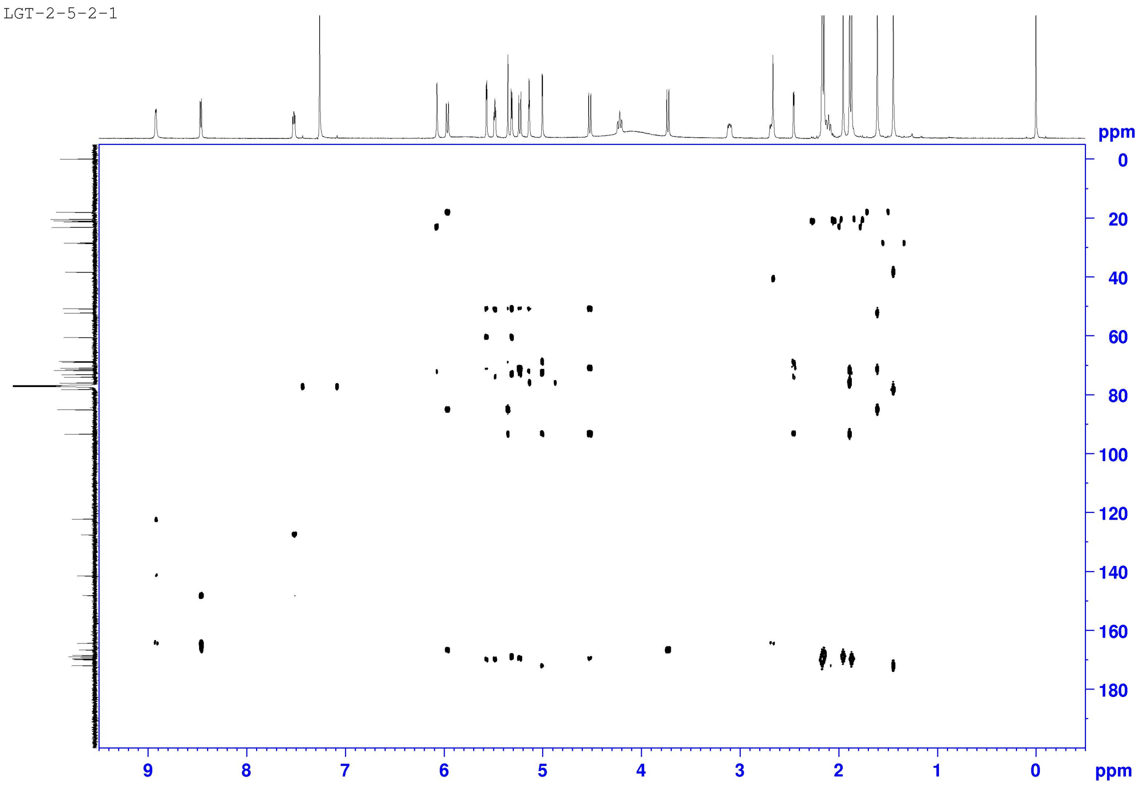

Figure S60. HMBC spectrum of compound **6**

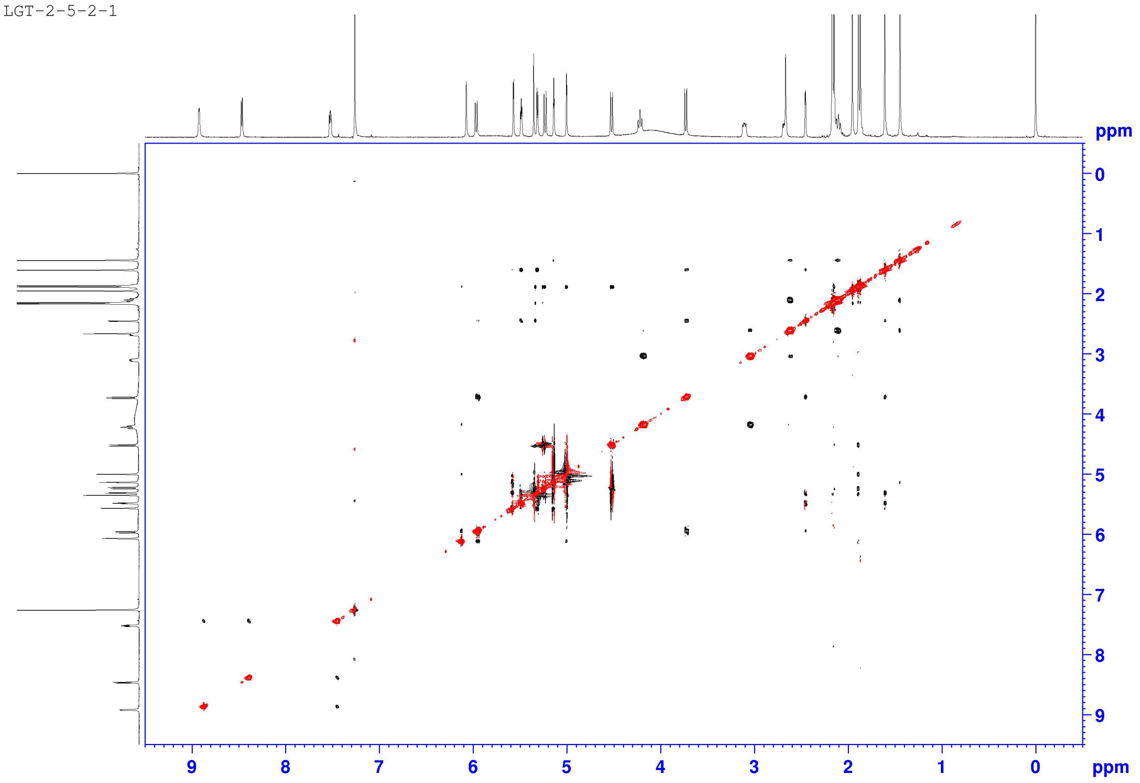

Figure S61. ROESY spectrum of compound **6**

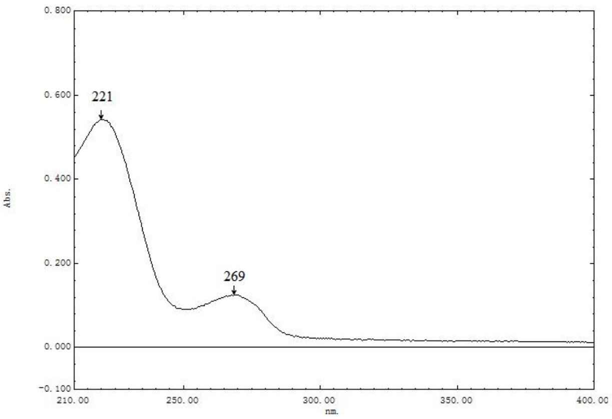

Figure S62. UV spectrum of compound **7**

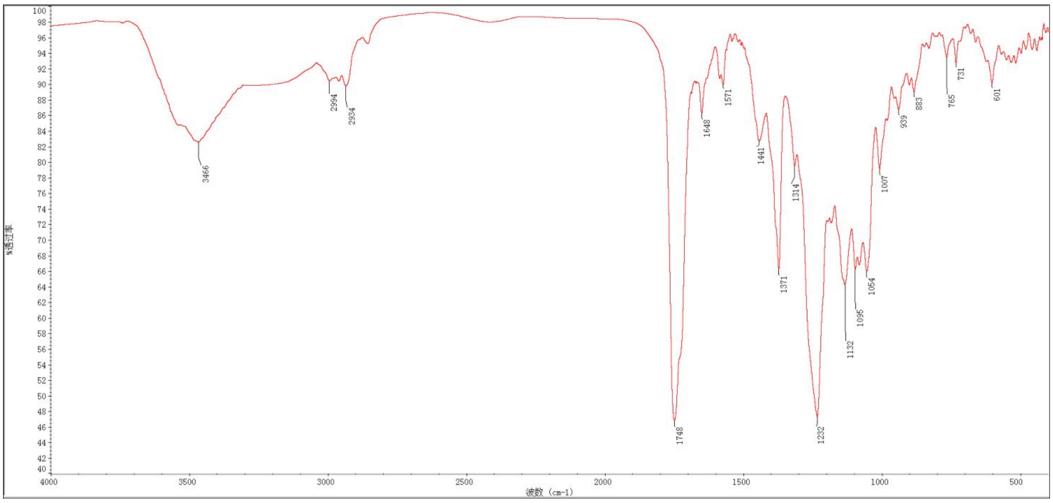

Figure S63. IR spectrum of compound **7**

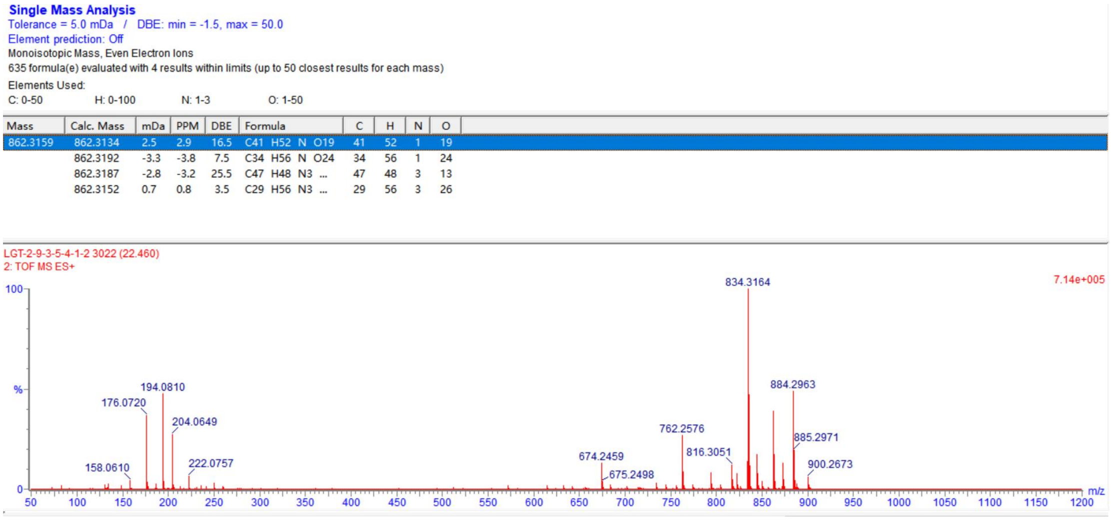

Mass	Calc. Mass	mDa	PPM	DBE	Formula	C	H	N	O
862.3159	862.3134	2.5	2.9	16.5	C41 H52 N O19	41	52	1	19
	862.3192	-3.3	-3.8	7.5	C34 H56 N O24	34	56	1	24
	862.3187	-2.8	-3.2	25.5	C47 H48 N3 ...	47	48	3	13
	862.3152	0.7	0.8	3.5	C29 H56 N3 ...	29	56	3	26

Figure S64. HRESIMS spectrum of compound **7**

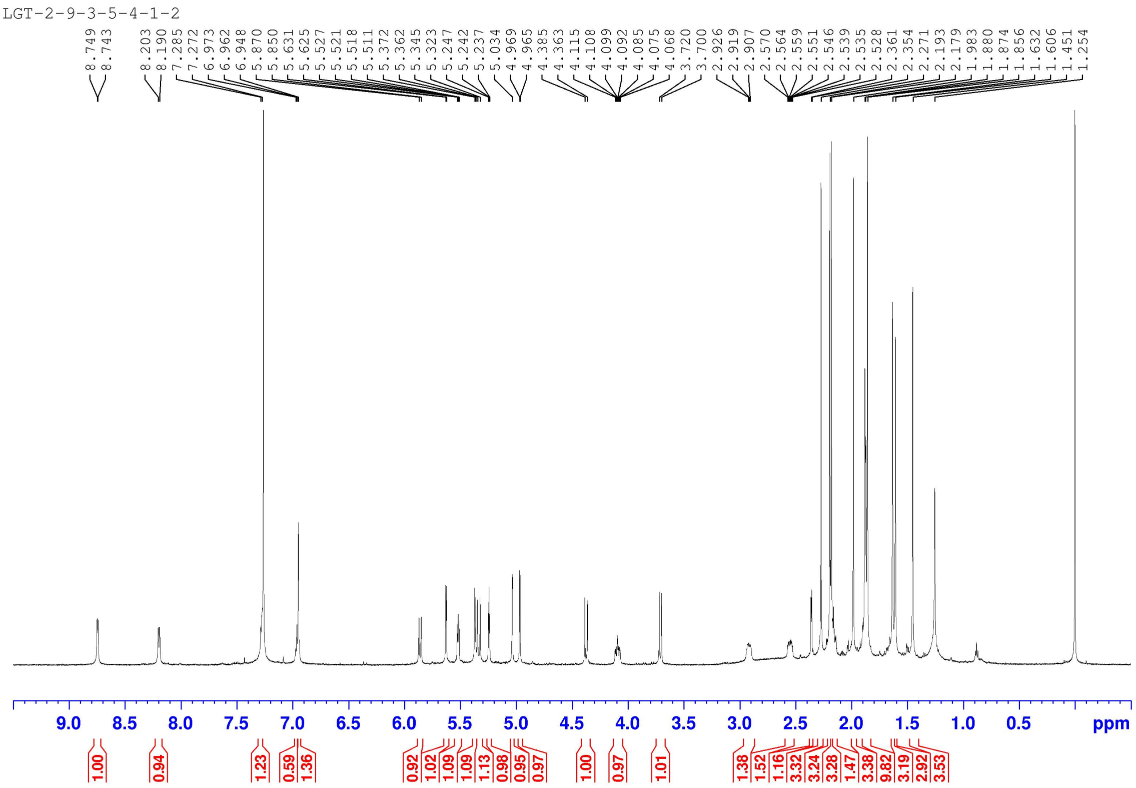

Figure S65. ^{1}H-NMR spectrum of compound **7** ($CDCl_3$, 600 MHz)

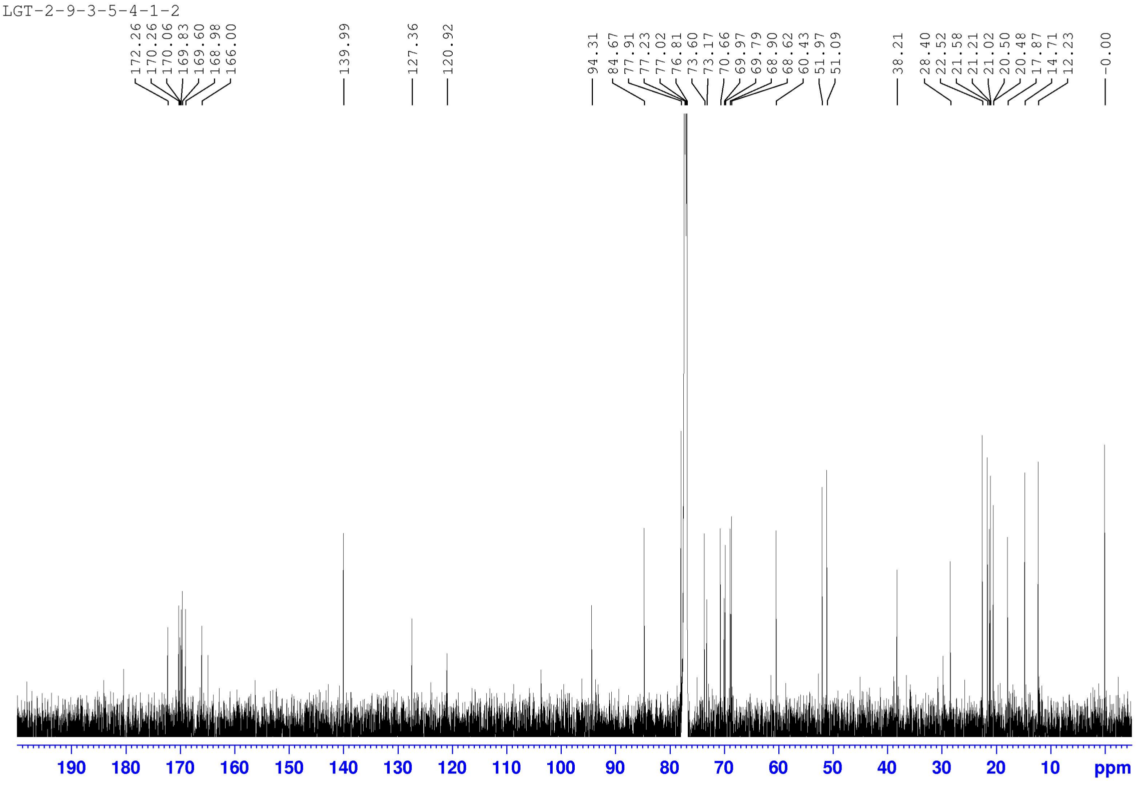

Figure S66. ^{13}C-NMR spectrum of compound **7** ($CDCl_3$, 150 MHz)

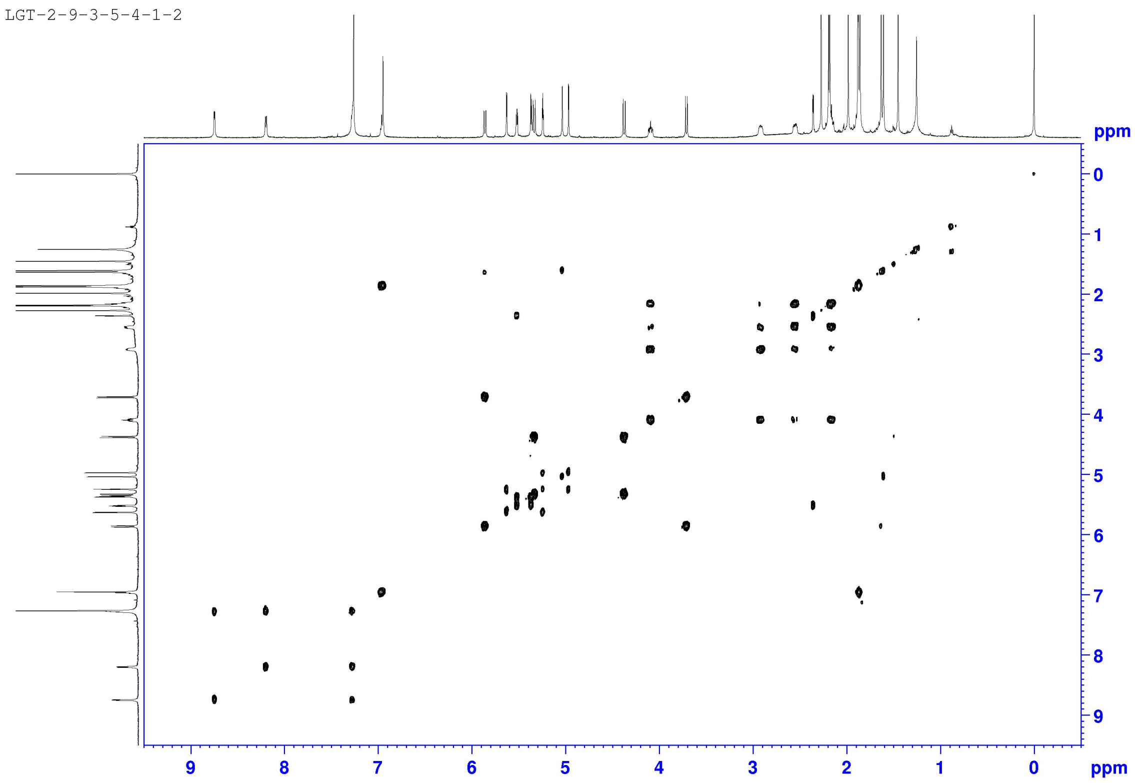

Figure S67. ^{1}H-^{1}H COSY spectrum of compound **7**

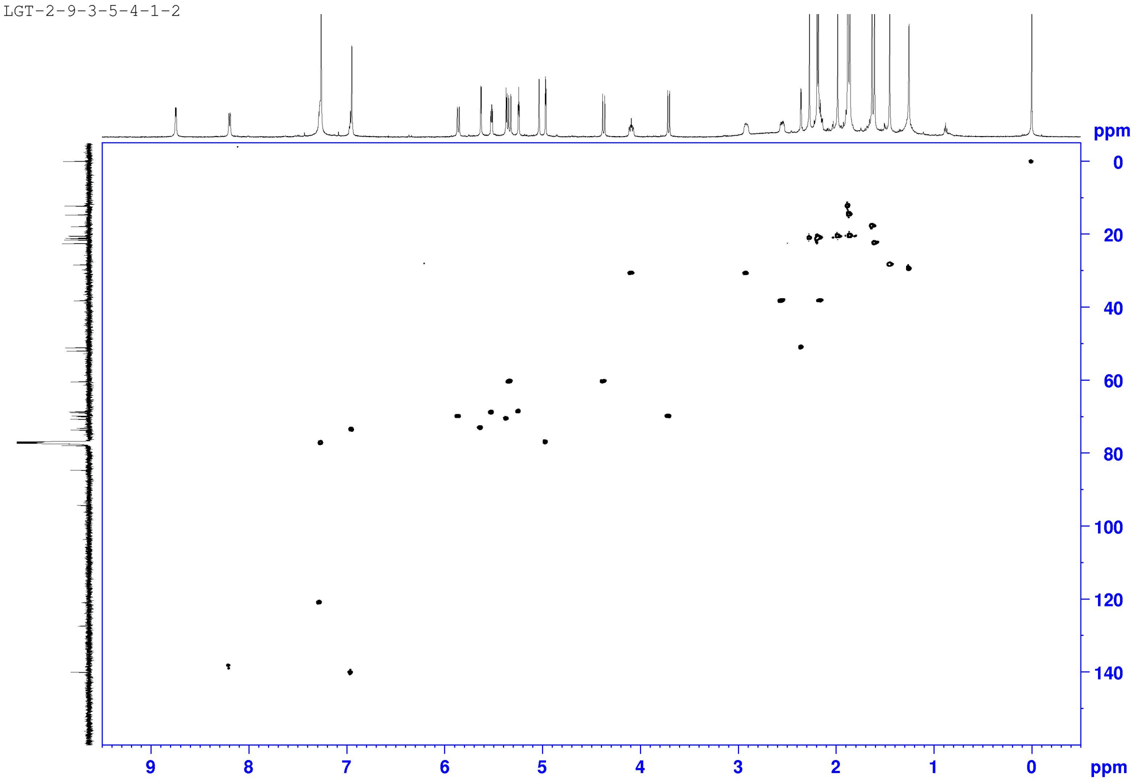

Figure S68. HSQC spectrum of compound **7**

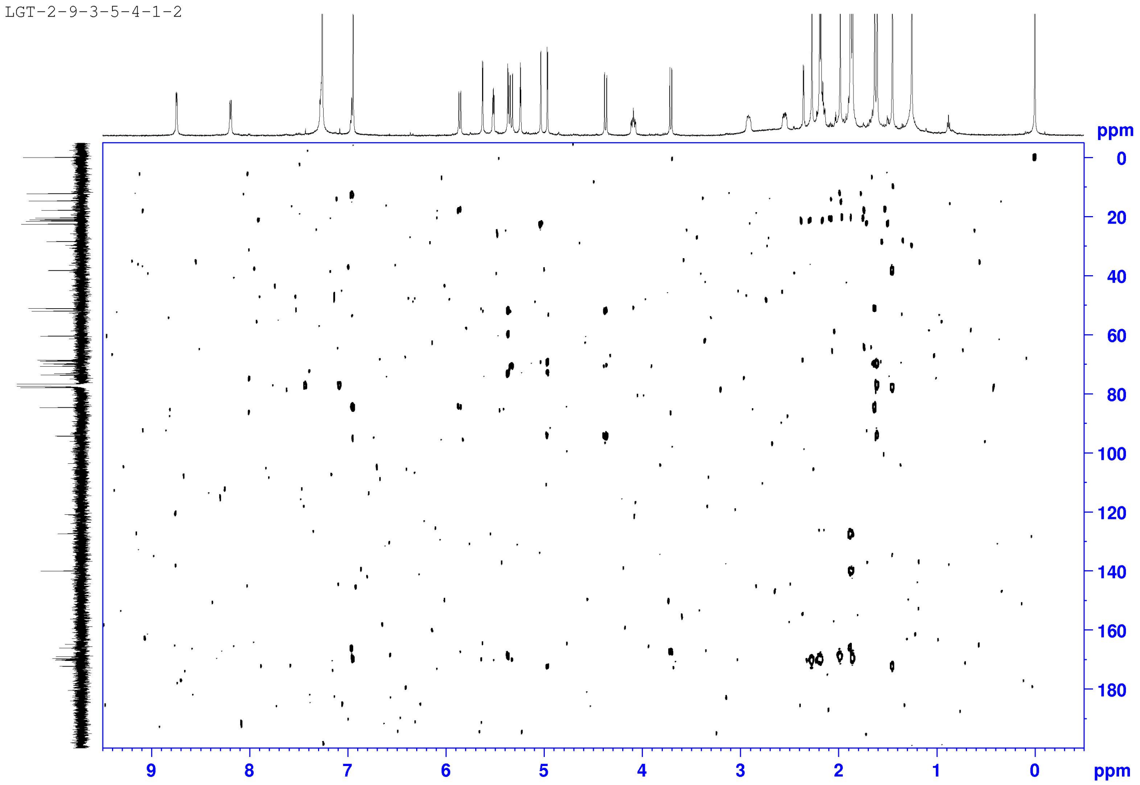

Figure S69. HMBC spectrum of compound **7**

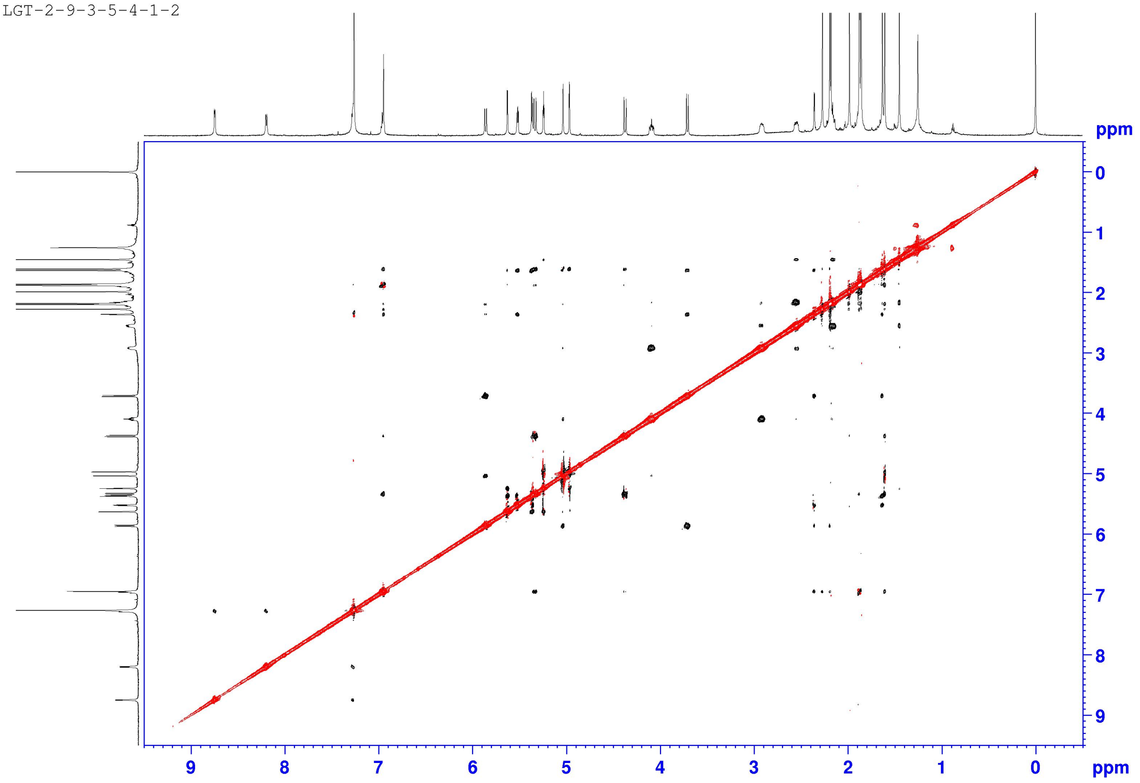

Figure S70. ROESY spectrum of compound **7**

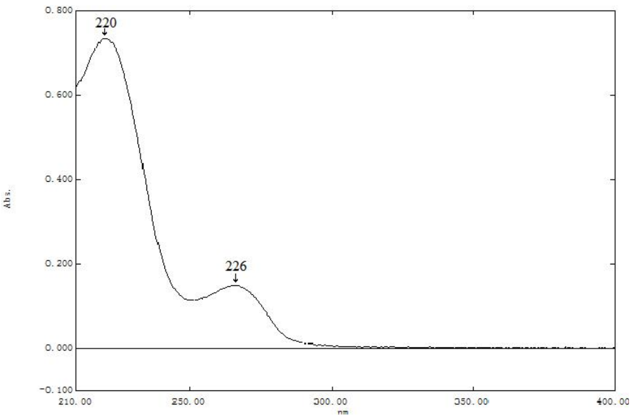

Figure S71. UV spectrum of compound **8**

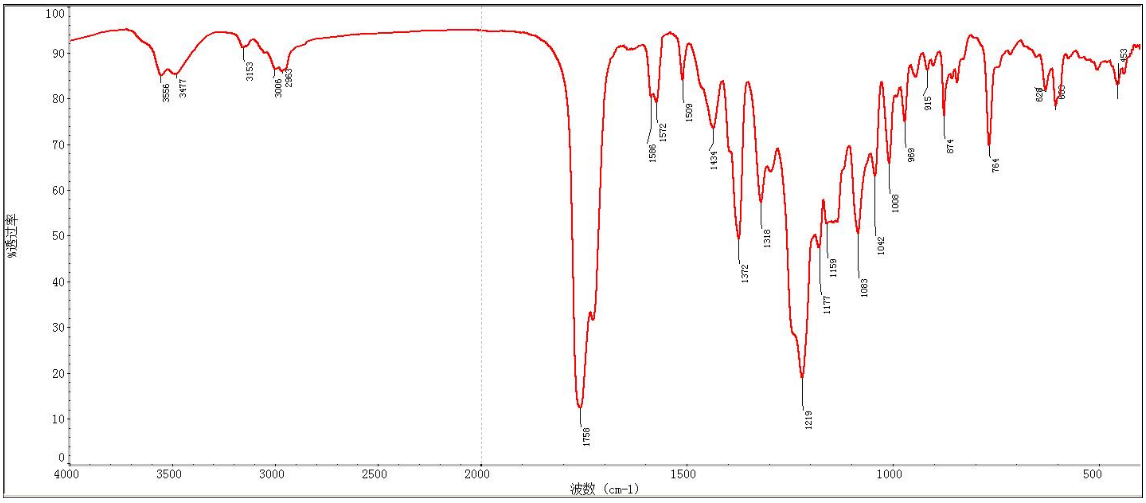

Figure S72. IR spectrum of compound **8**

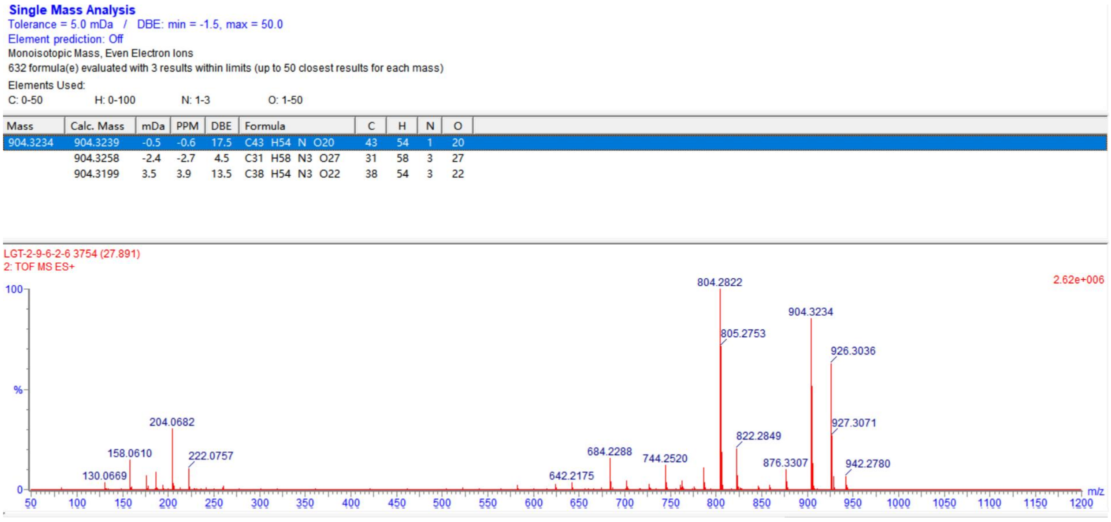
Single Mass Analysis
Tolerance = 5.0 mDa / DBE: min = -1.5, max = 50.0
Element prediction: Off
Monoisotopic Mass, Even Electron Ions
632 formula(e) evaluated with 3 results within limits (up to 50 closest results for each mass)
Elements Used:
C: 0-50 H: 0-100 N: 1-3 O: 1-50

Mass	Calc. Mass	mDa	PPM	DBE	Formula	C	H	N	O
904.3234	904.3239	-0.5	-0.6	17.5	C43 H54 N O20	43	54	1	20
	904.3258	-2.4	-2.7	4.5	C31 H58 N3 O27	31	58	3	27
	904.3199	3.5	3.9	13.5	C38 H54 N3 O22	38	54	3	22

Figure S73. HRESIMS spectrum of compound **8**

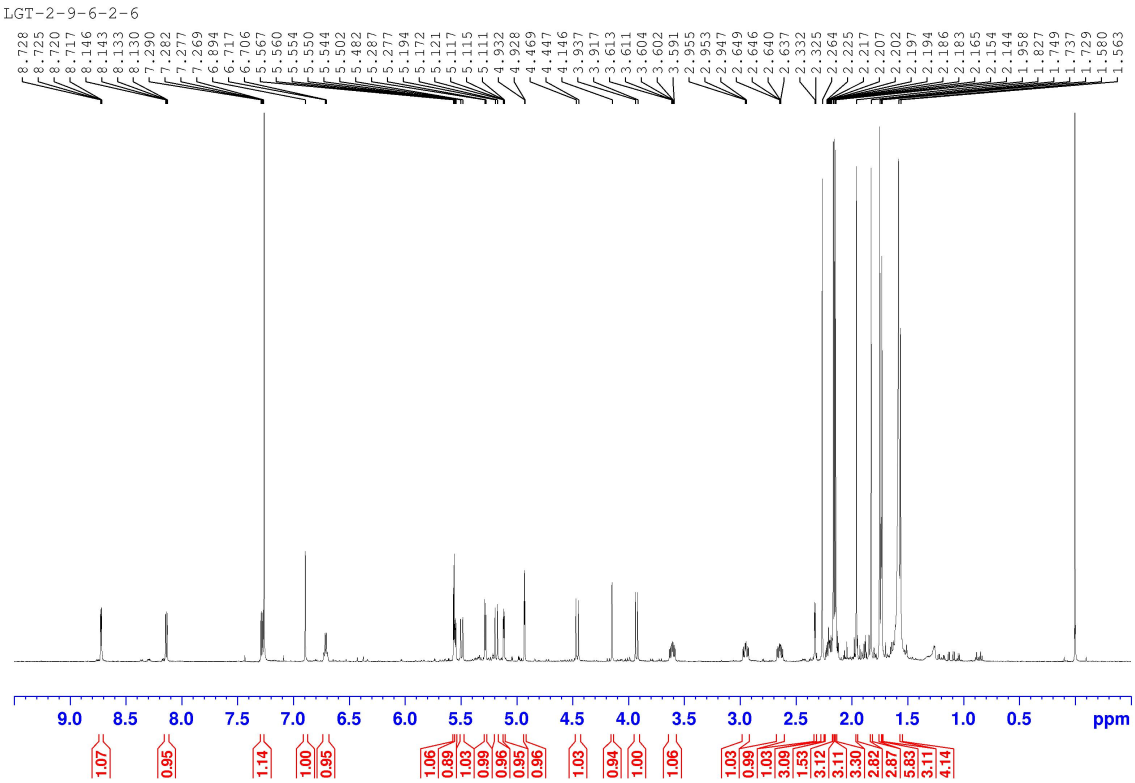

Figure S74. ^{1}H-NMR spectrum of compound **8** ($CDCl_3$, 600 MHz)

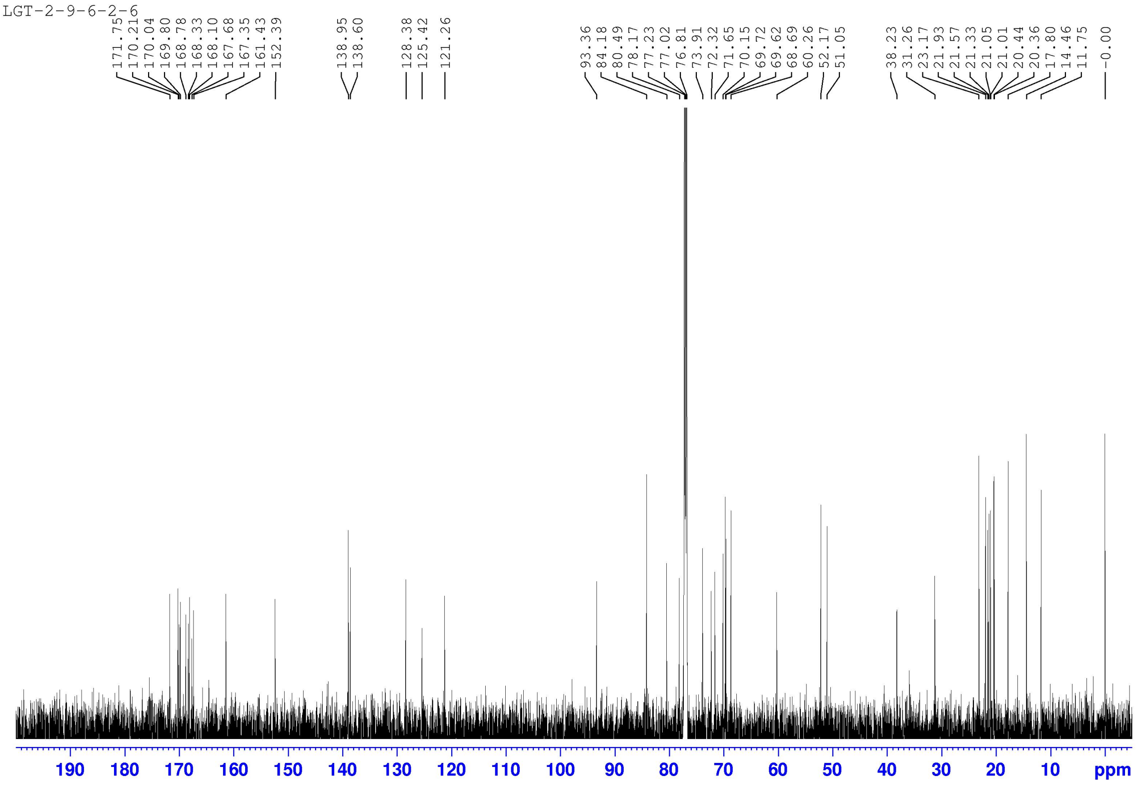

Figure S75. ^{13}C-NMR spectrum of compound **8** ($CDCl_3$, 150 MHz)

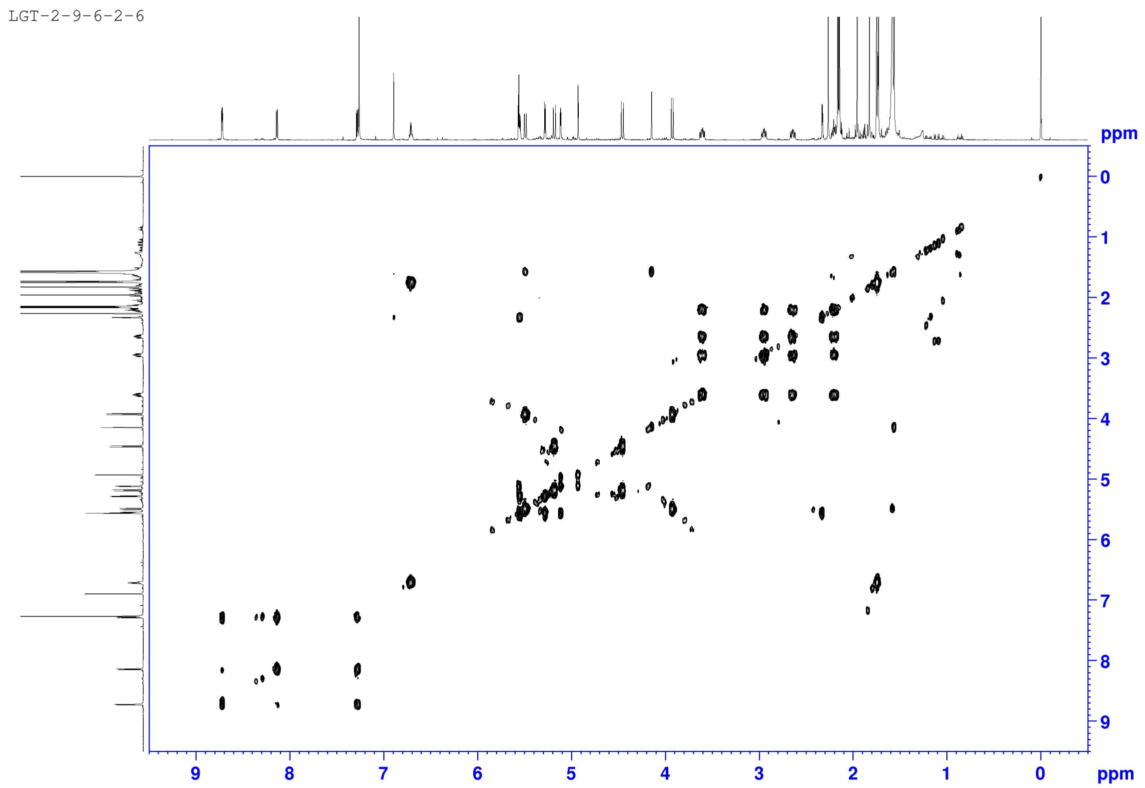

Figure S76. ^{1}H-^{1}H COSY spectrum of compound **8**

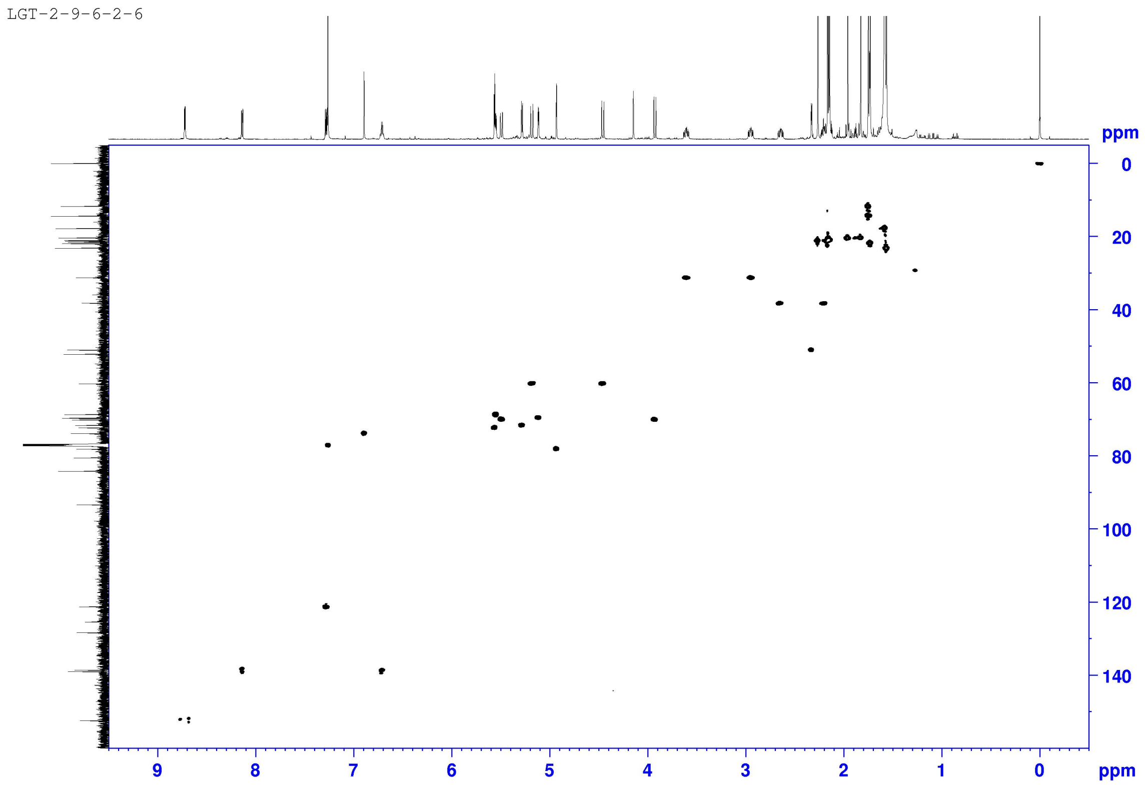

Figure S77. HSQC spectrum of compound **8**

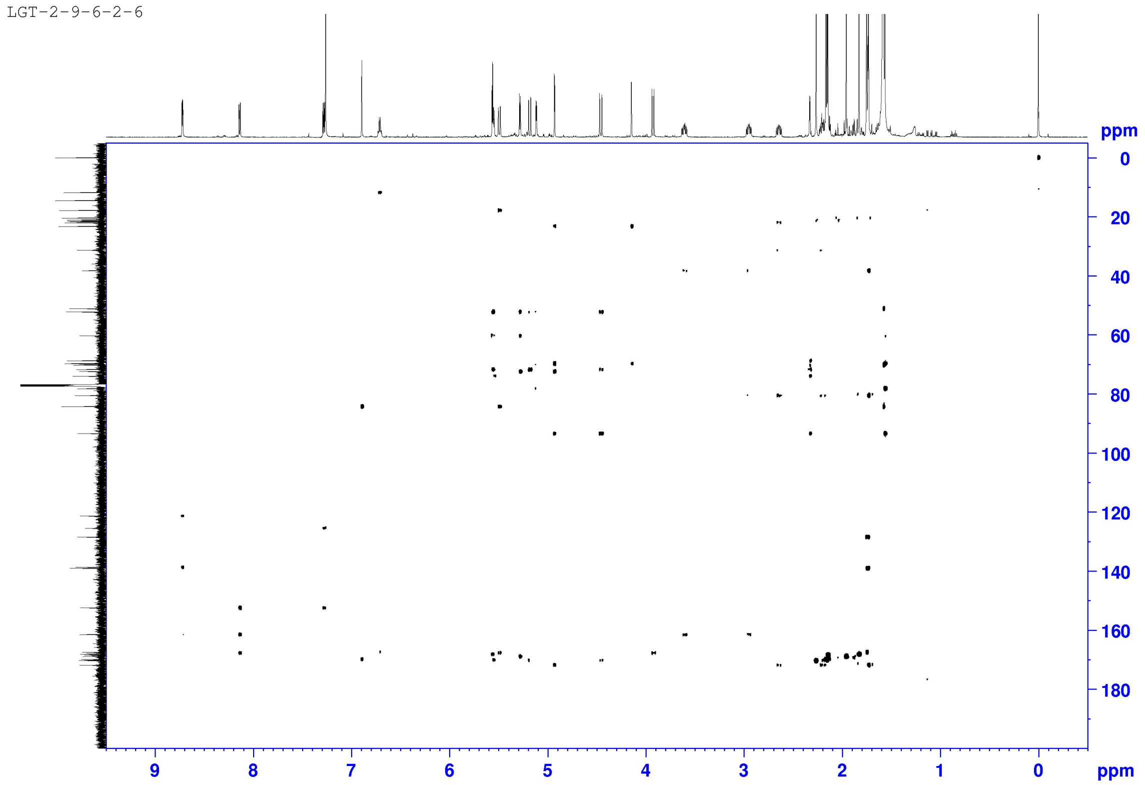

Figure S78. HMBC spectrum of compound **8**

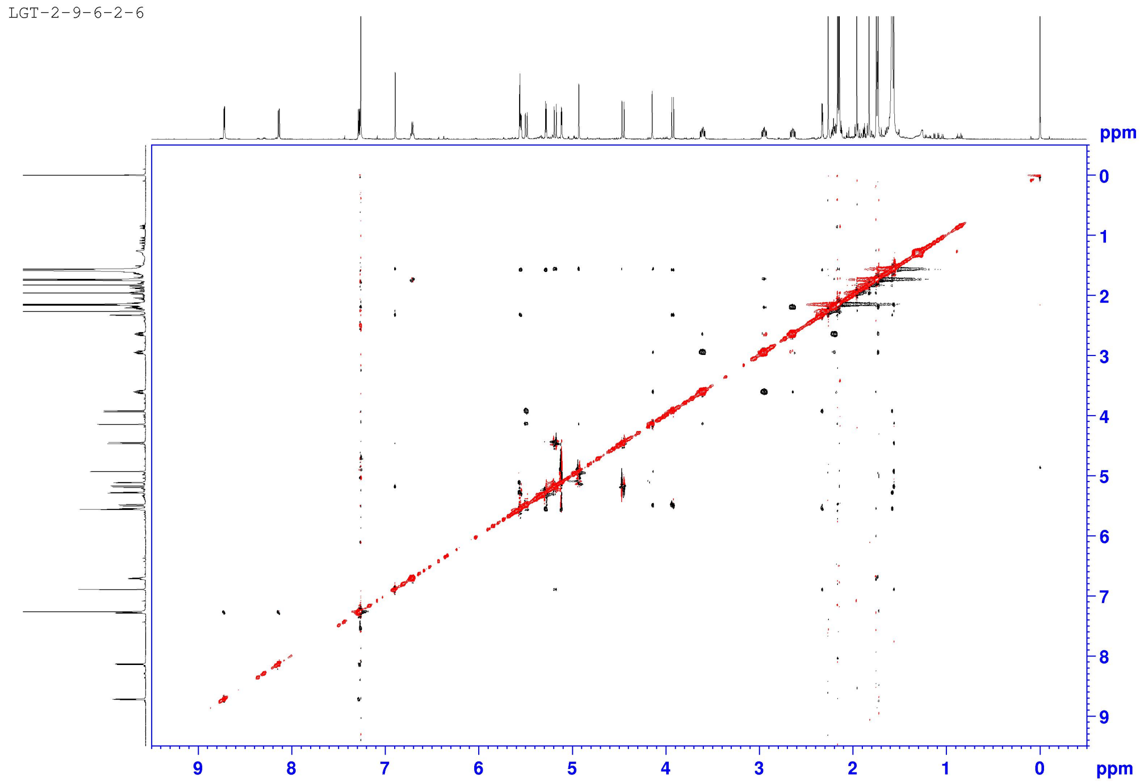

Figure S79. ROESY spectrum of compound **8**

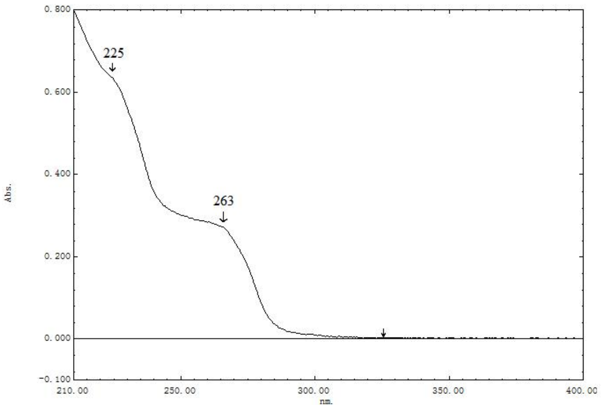

Figure S80. UV spectrum of compound **9**

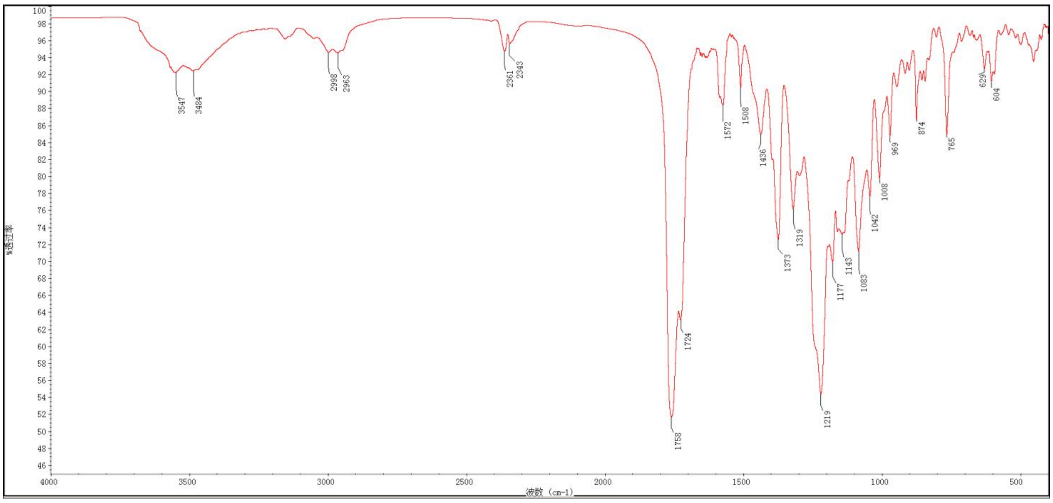

Figure S81. IR spectrum of compound **9**

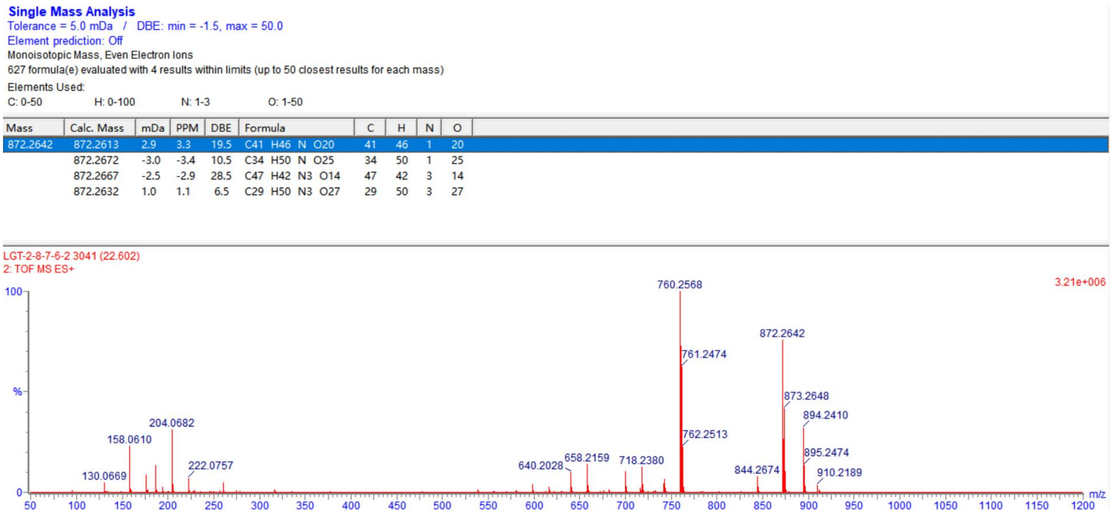

Figure S82. HRESIMS spectrum of compound **9**

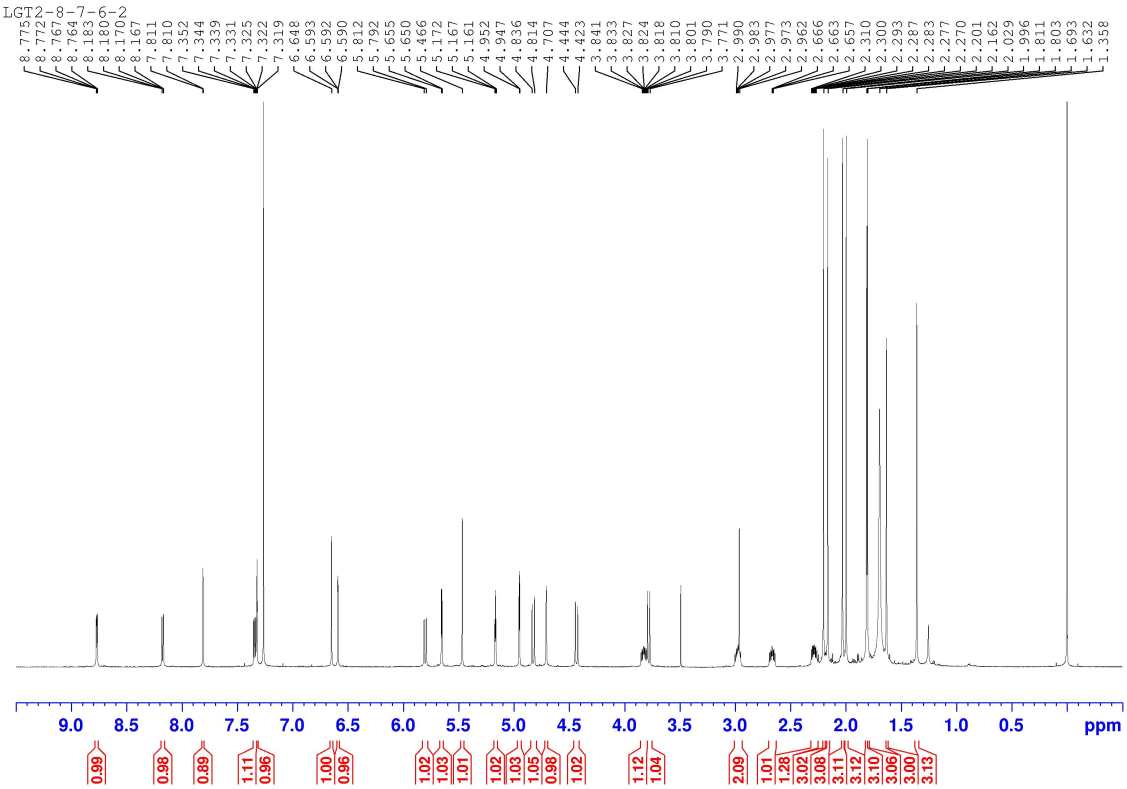

Figure S83. ^{1}H-NMR spectrum of compound **9** ($CDCl_3$, 600 MHz)

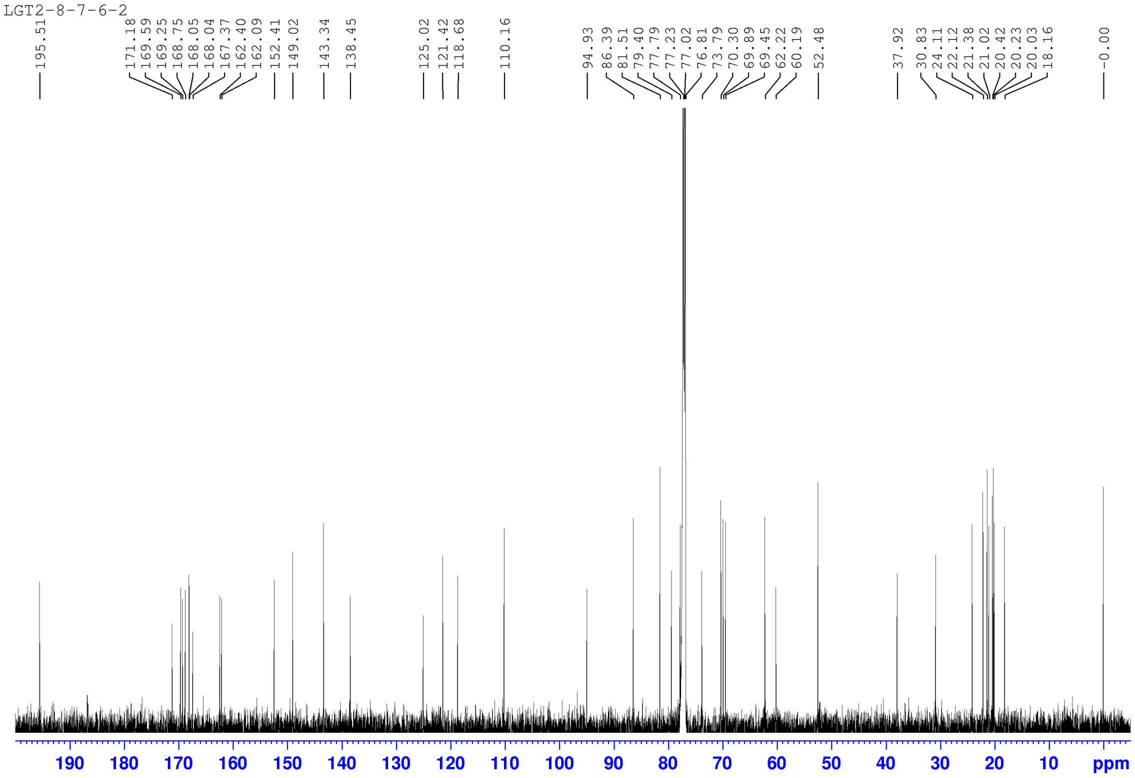

Figure S84. ^{13}C-NMR spectrum of compound **9** ($CDCl_3$, 150 MHz)

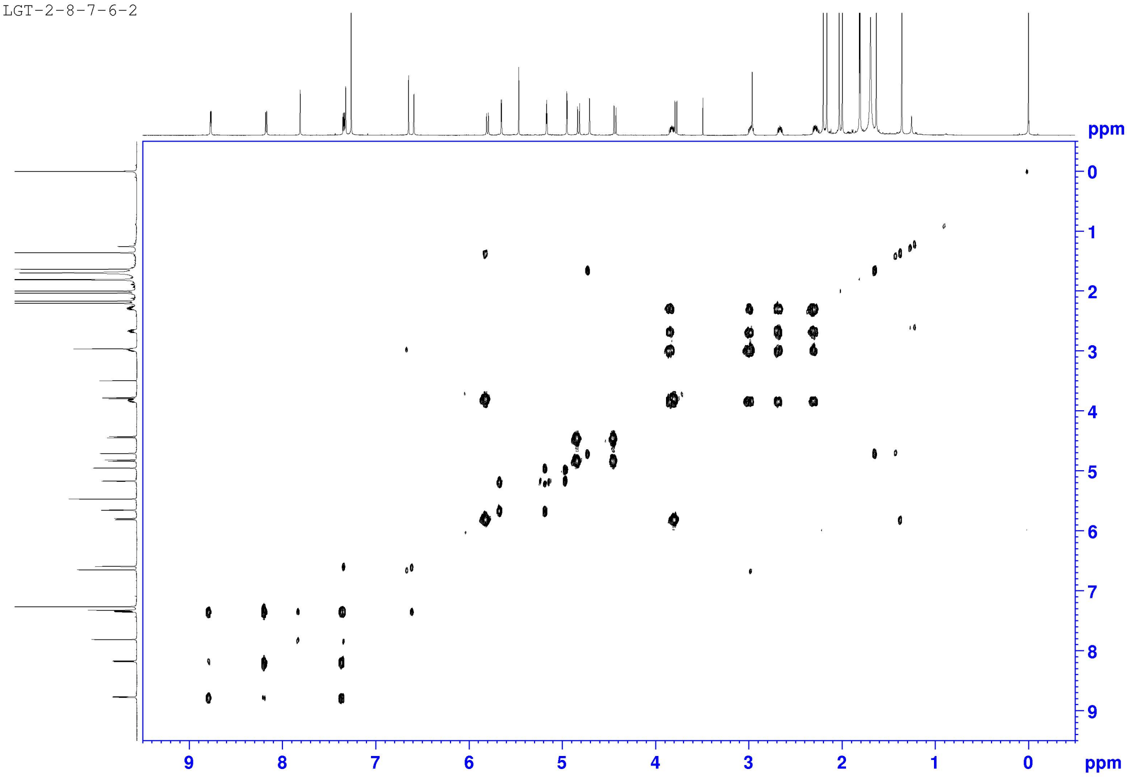

Figure S85. ^{1}H-^{1}H COSY spectrum of compound **9**

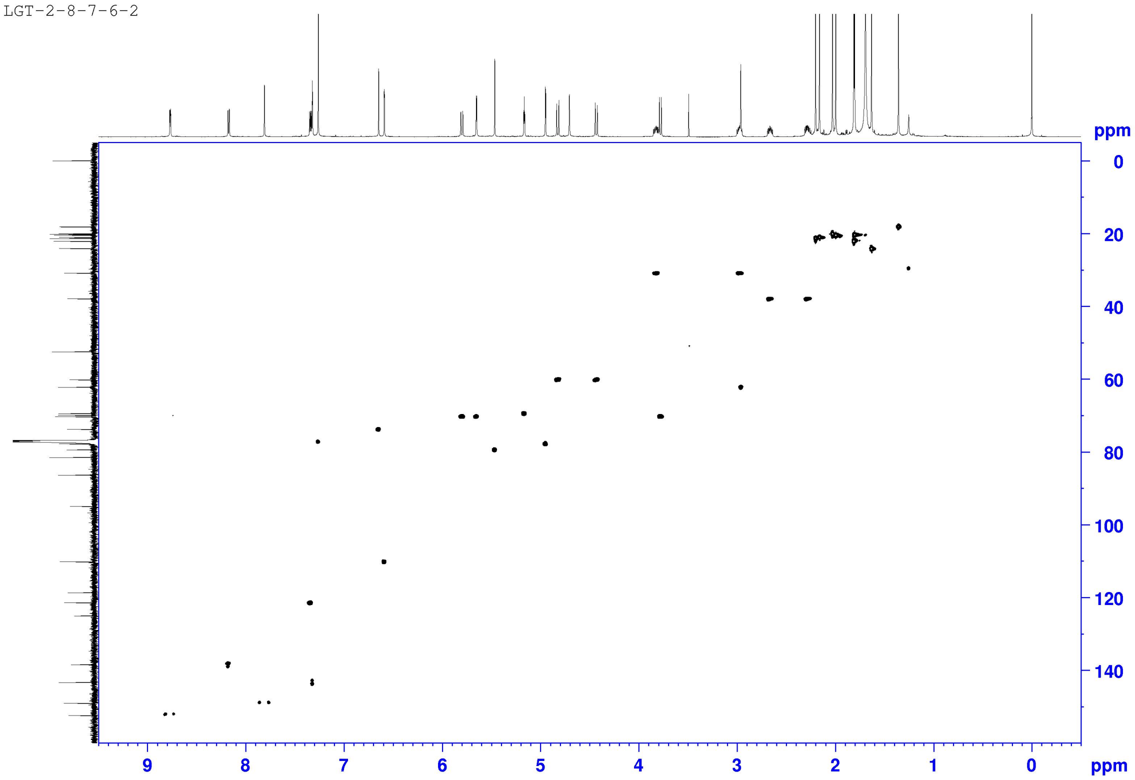

Figure S86. HSQC spectrum of compound **9**

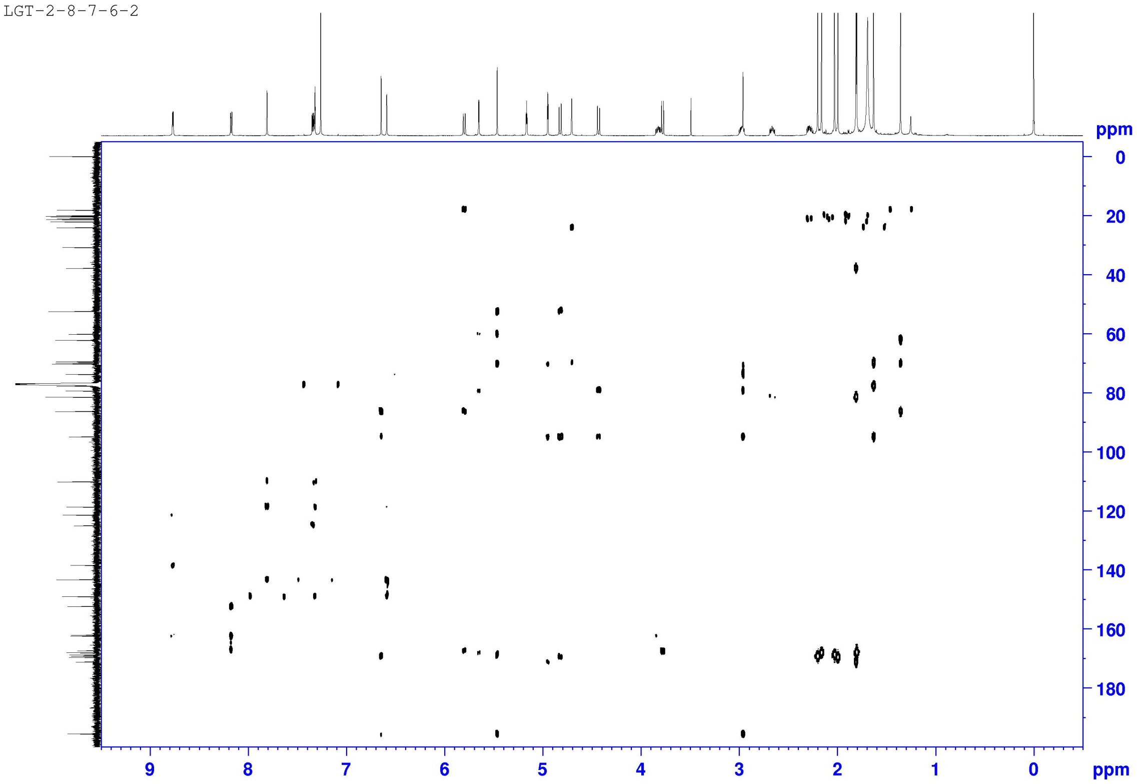

Figure S87. HMBC spectrum of compound **9**

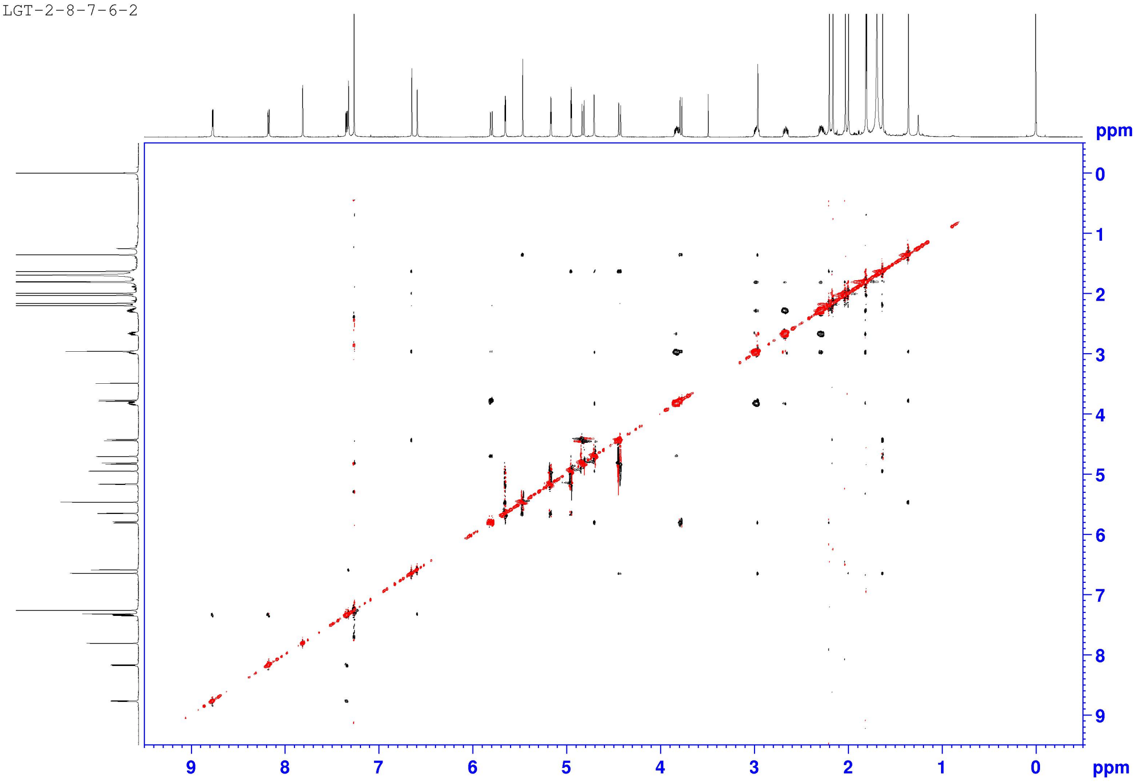

Figure S88. ROESY spectrum of compound **9**

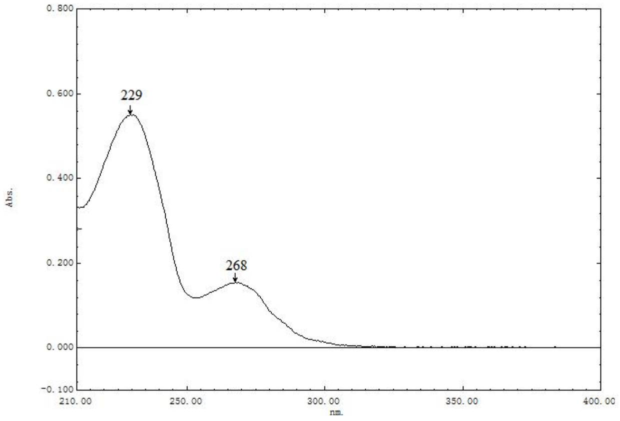

Figure S89. UV spectrum of compound **10**

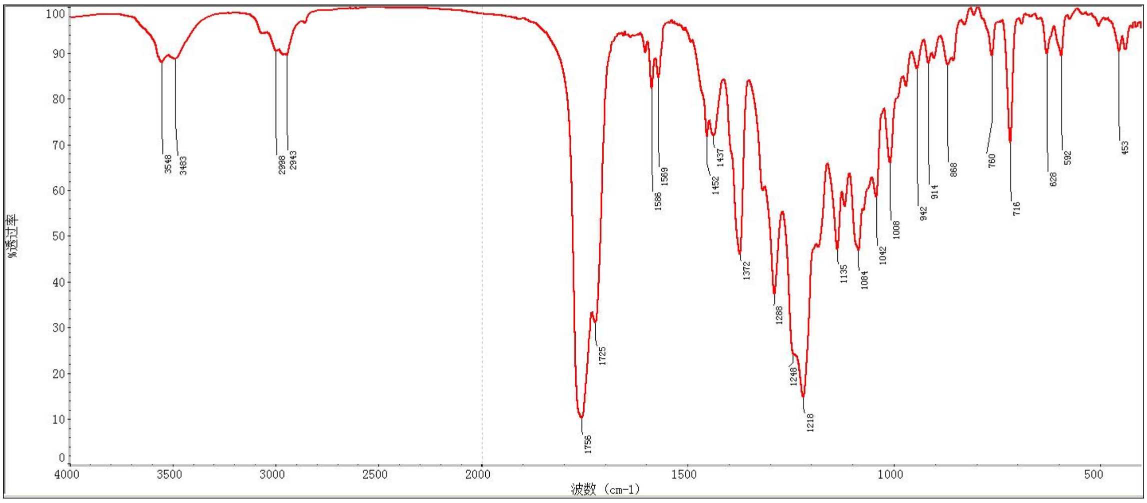

Figure S90. IR spectrum of compound **10**

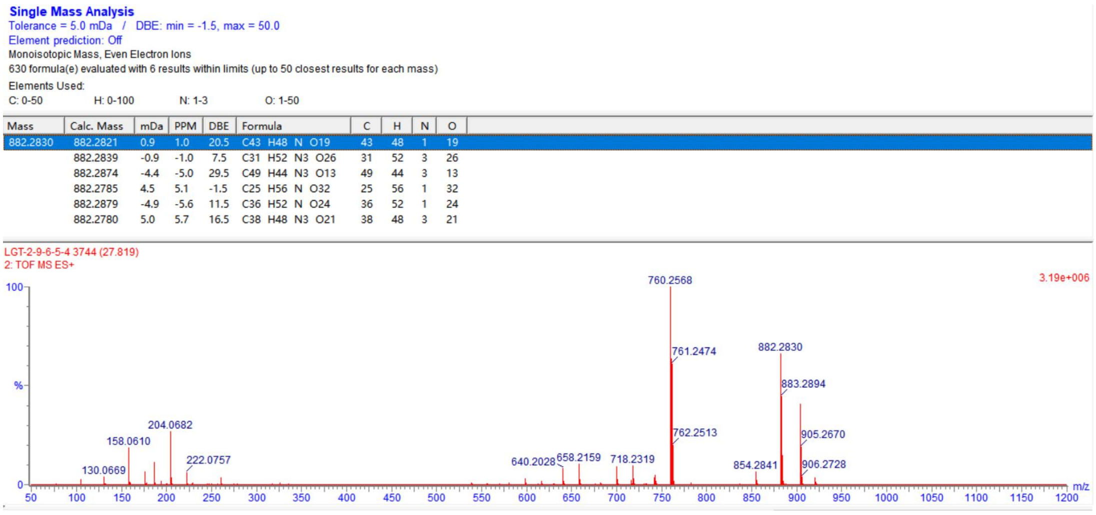

Figure S91. HRESIMS spectrum of compound **10**

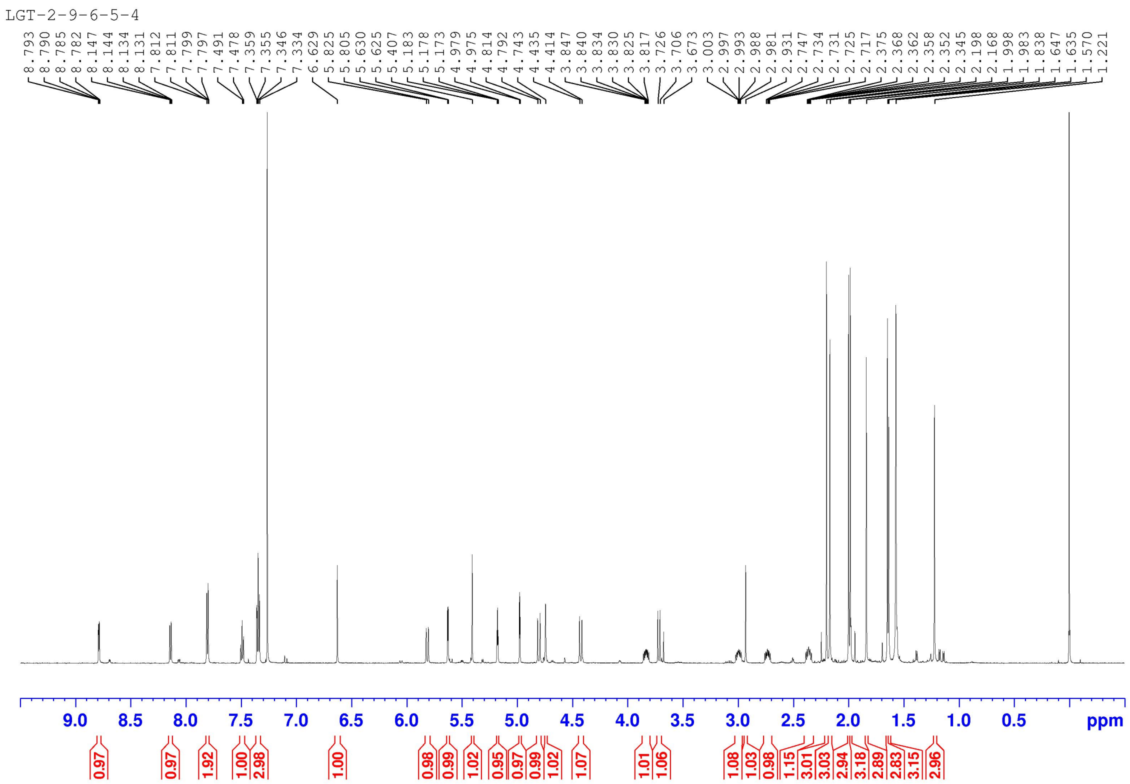

Figure S92. ^{1}H-NMR spectrum of compound **10** ($CDCl_3$, 600 MHz)

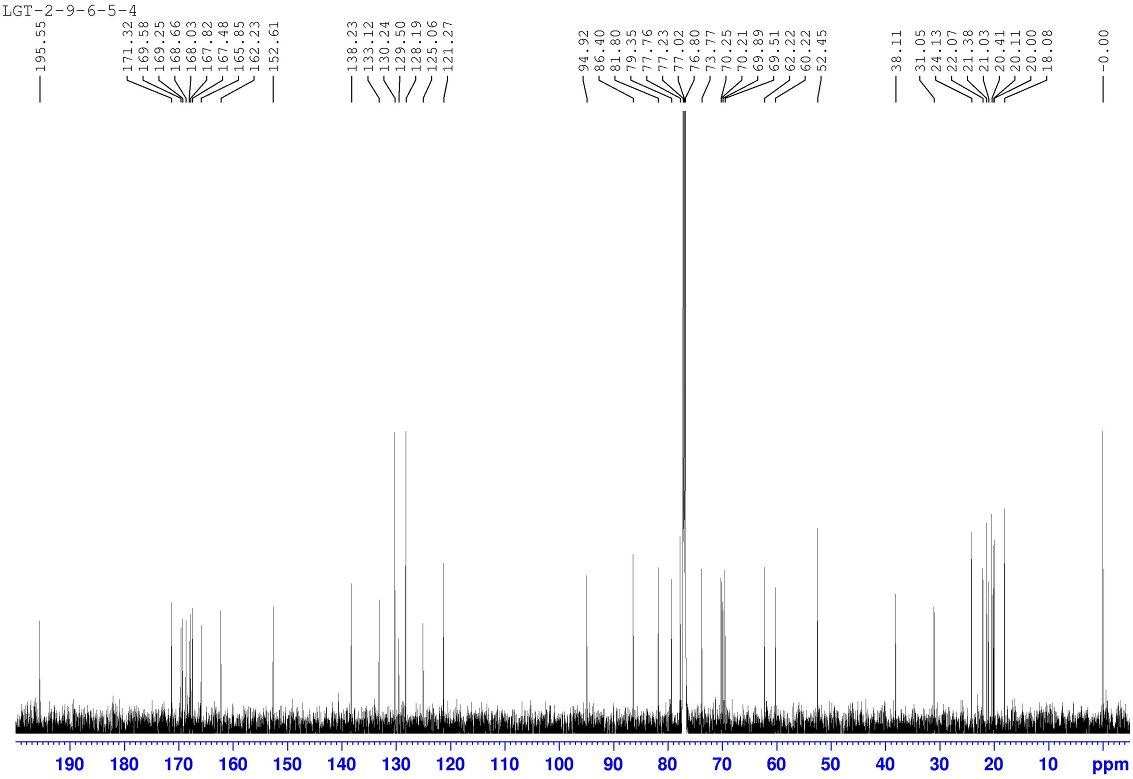

Figure S93. ^{13}C-NMR spectrum of compound **10** ($CDCl_3$, 150 MHz)

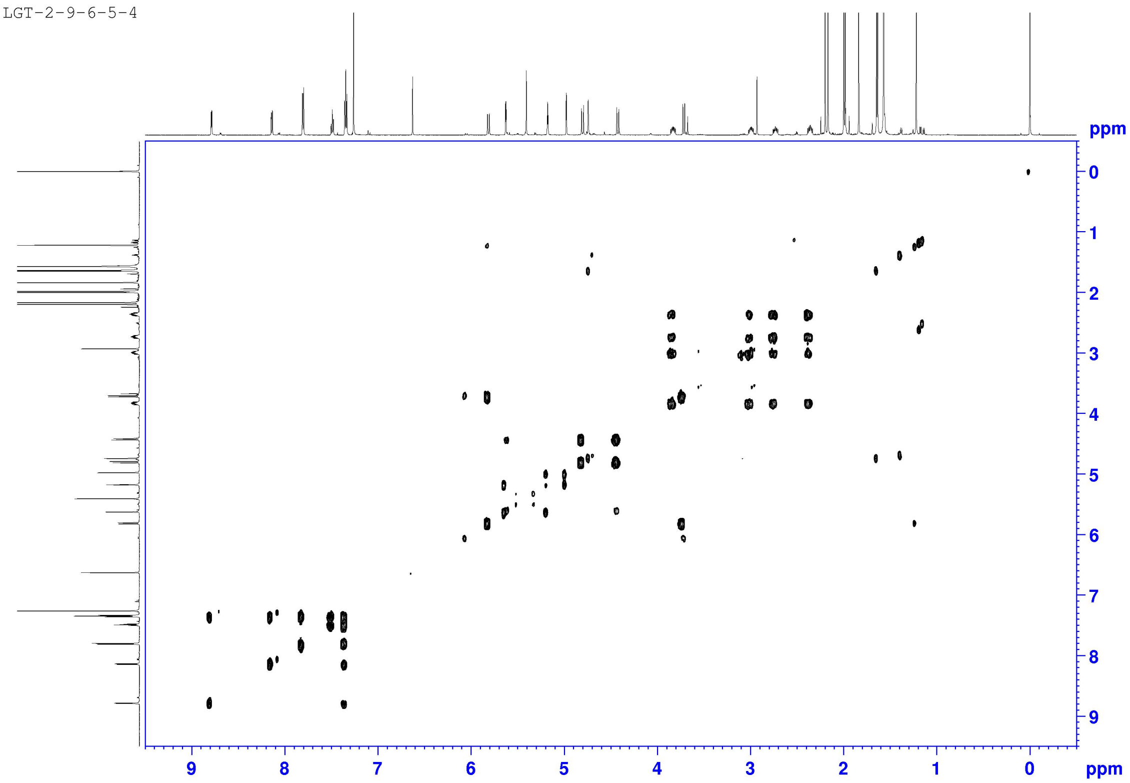

Figure S94. ^{1}H-^{1}H COSY spectrum of compound **10**

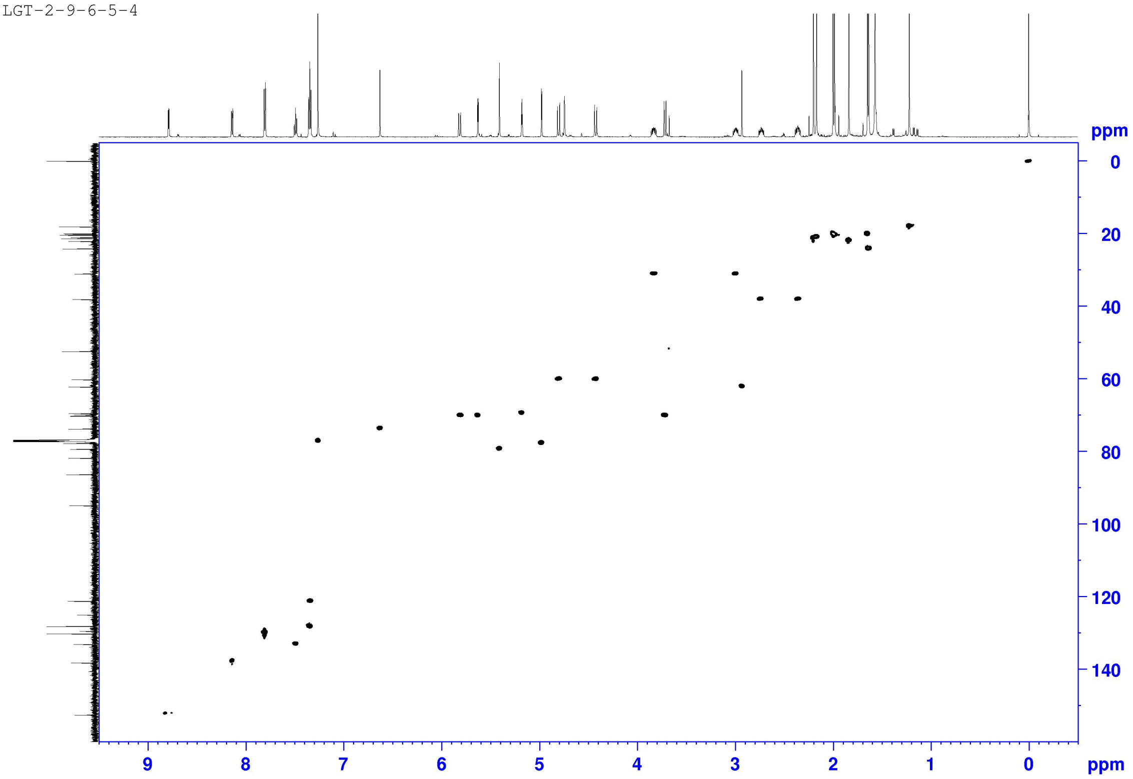

Figure S95. HSQC spectrum of compound **10**

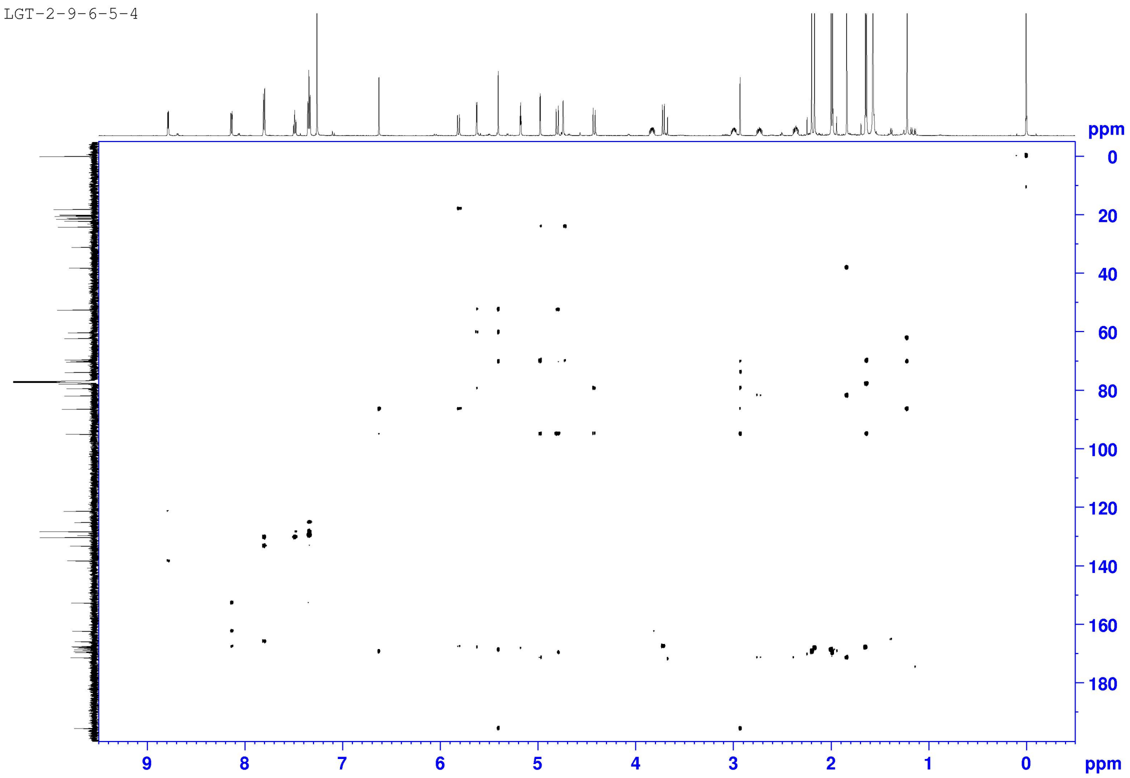

Figure S96. HMBC spectrum of compound **10**

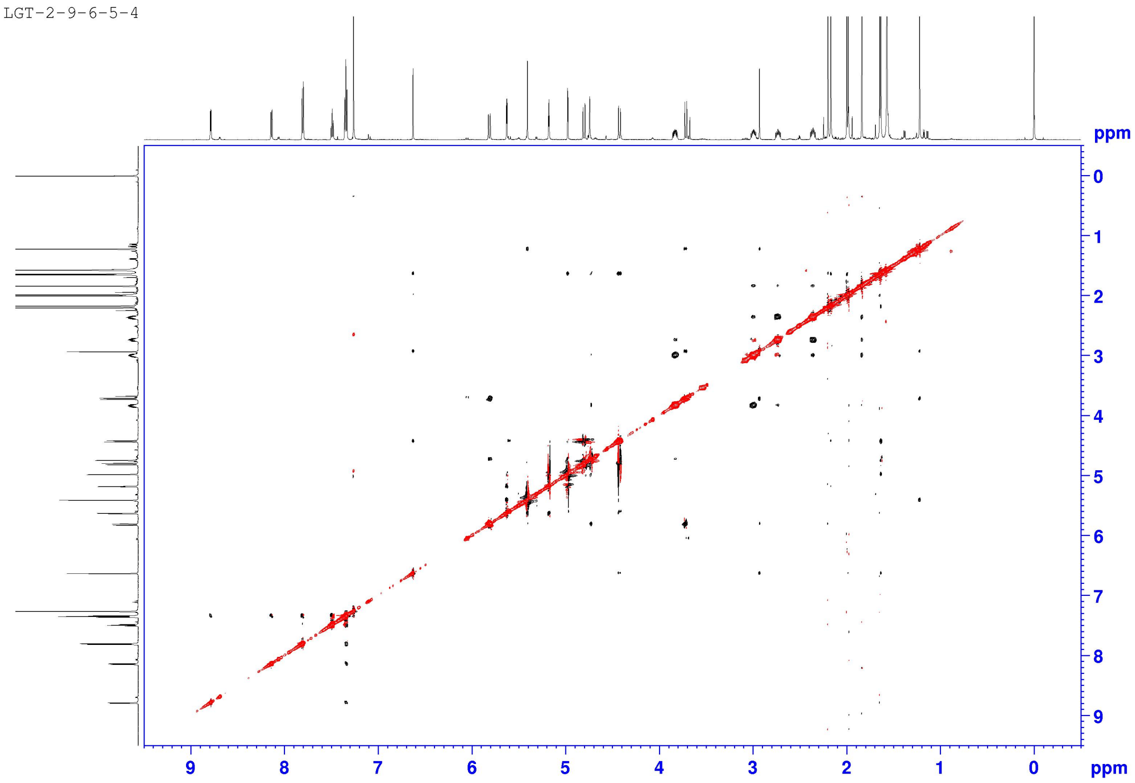

Figure S97. ROESY spectrum of compound **10**

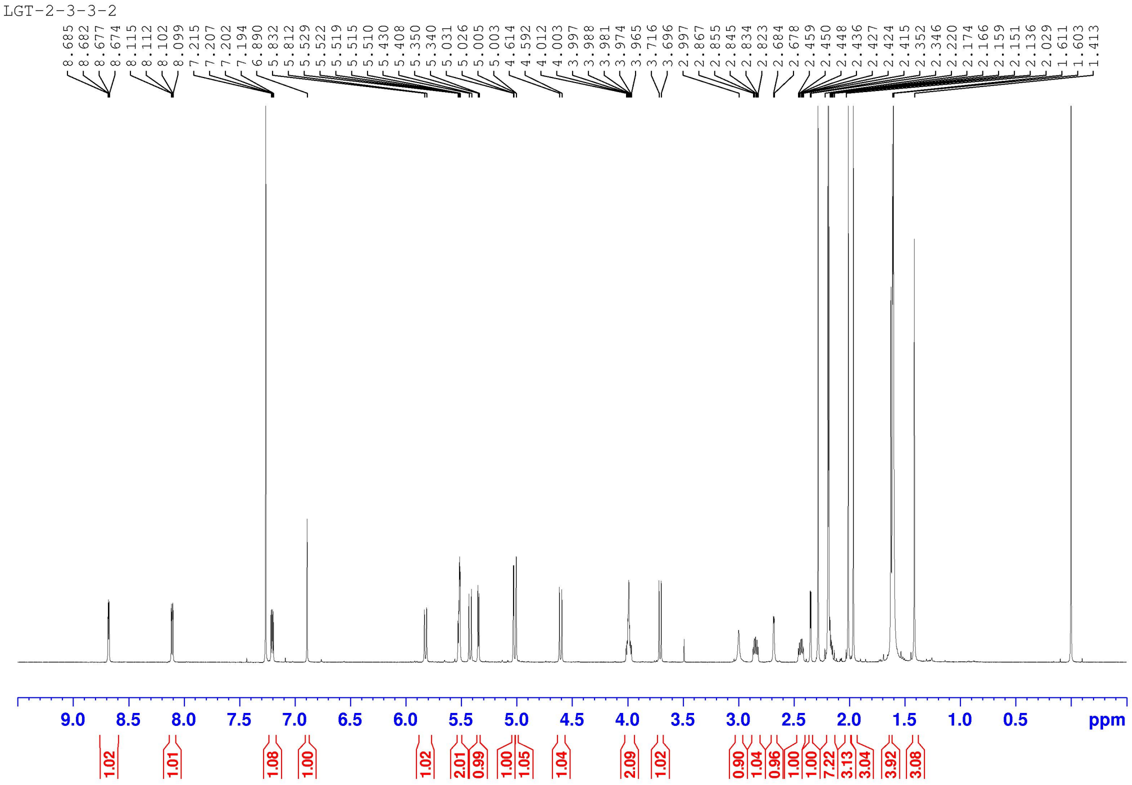

Figure S98. ^{1}H-NMR spectrum of compound **11** ($CDCl_3$, 600 MHz)

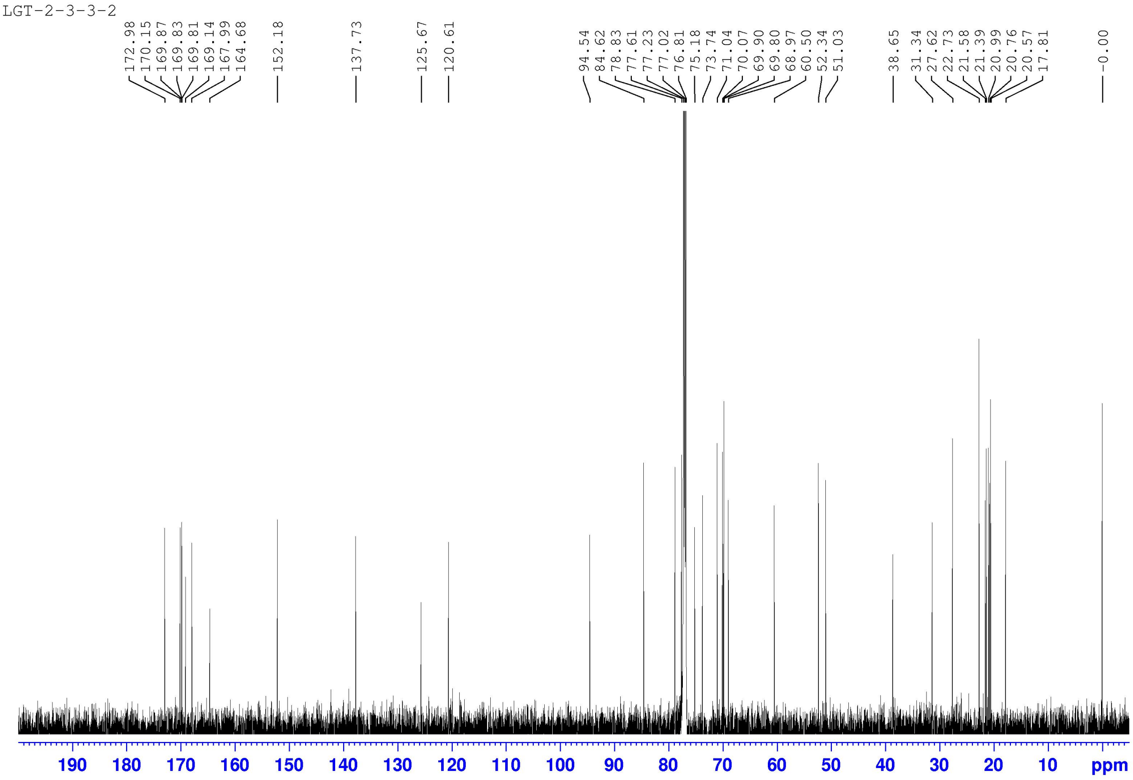

Figure S99. ^{13}C-NMR spectrum of compound **11** ($CDCl_3$, 150 MHz)

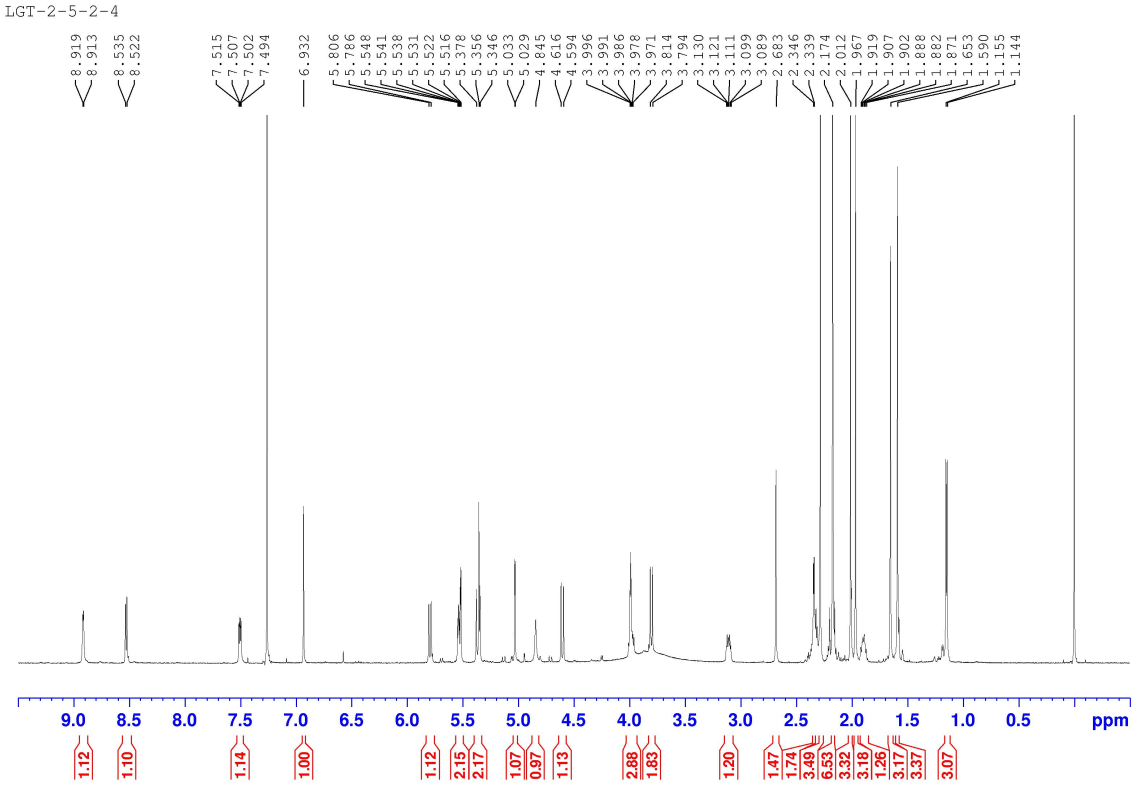

Figure S100. ^{1}H-NMR spectrum of compound **12** ($CDCl_3$, 600 MHz)

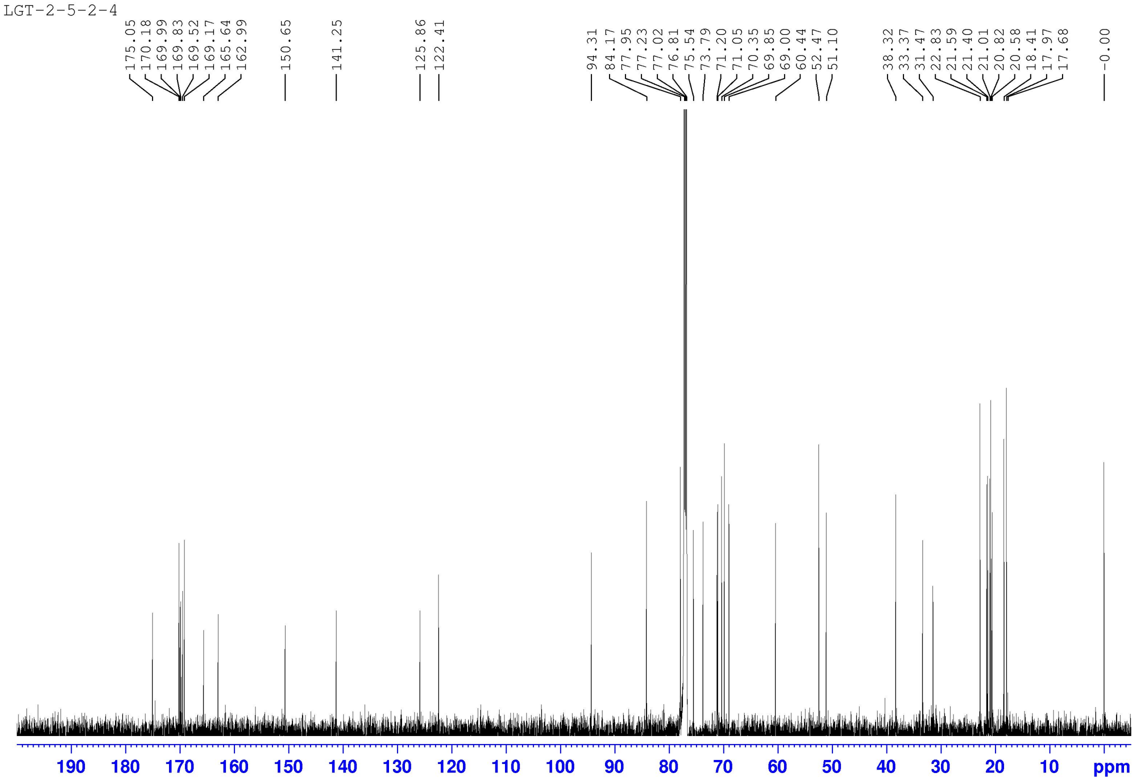

Figure S101. ^{13}C-NMR spectrum of compound **12** ($CDCl_3$, 150 MHz)

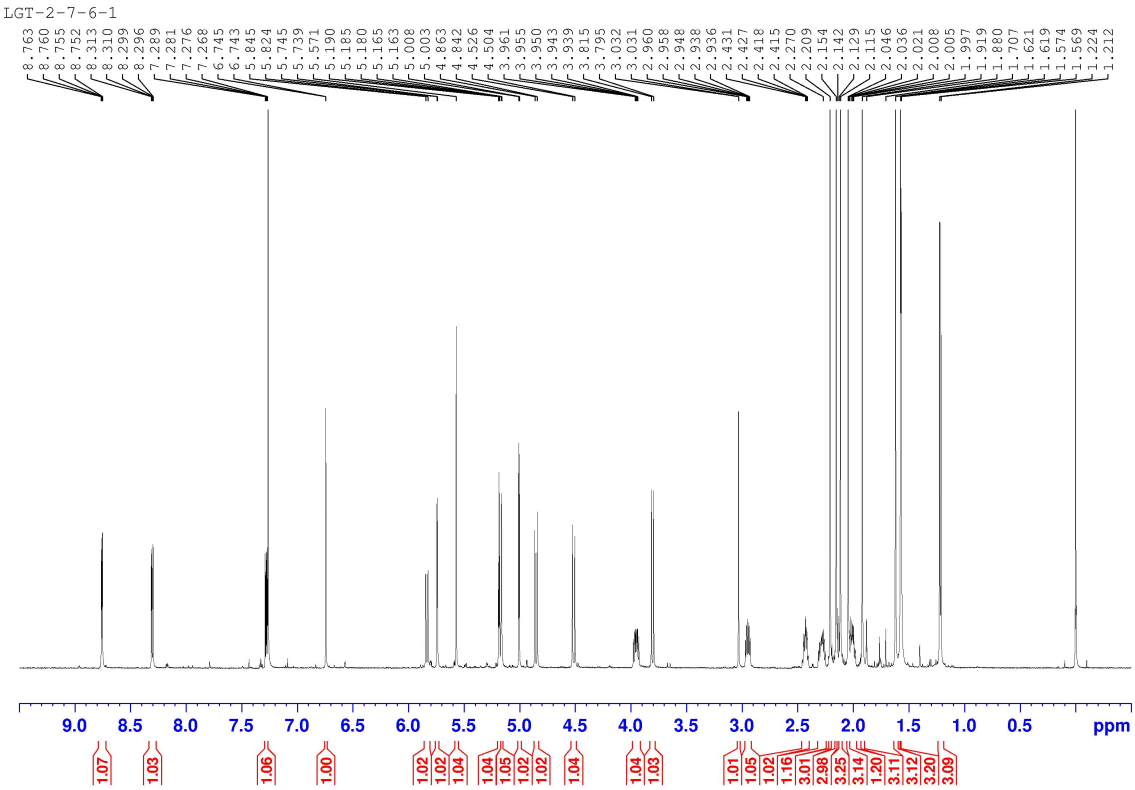

Figure S102. ^{1}H-NMR spectrum of compound **13** ($CDCl_3$, 600 MHz)

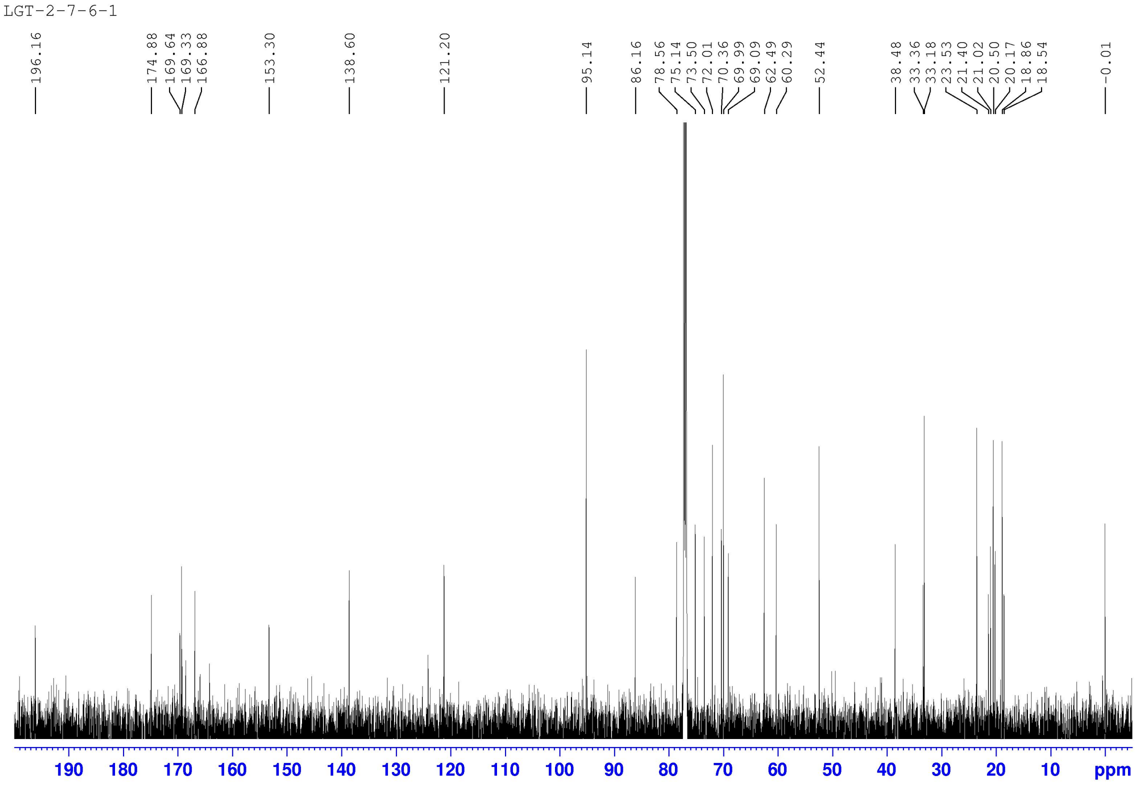

Figure S103. ^{13}C-NMR spectrum of compound **13** ($CDCl_3$, 150 MHz)

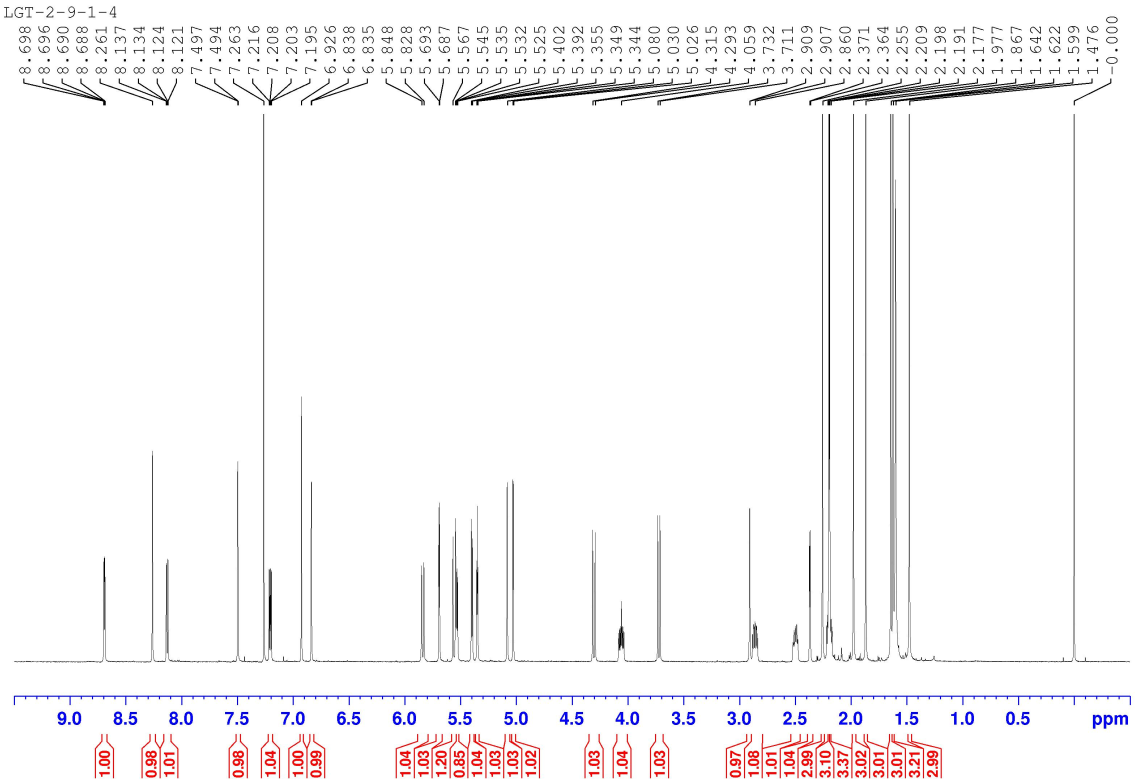

Figure S104. ^{1}H-NMR spectrum of compound **14** ($CDCl_3$, 600 MHz)

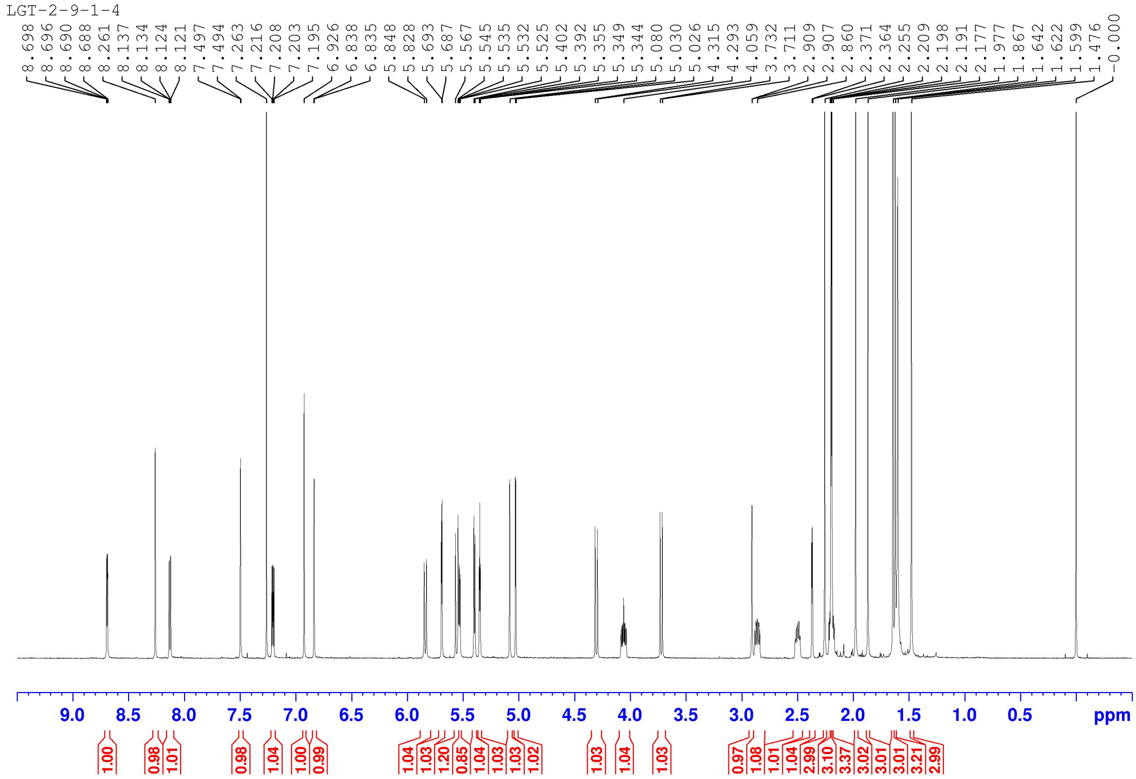

Figure S105. ^{13}C-NMR spectrum of compound **14** ($CDCl_3$, 150 MHz)

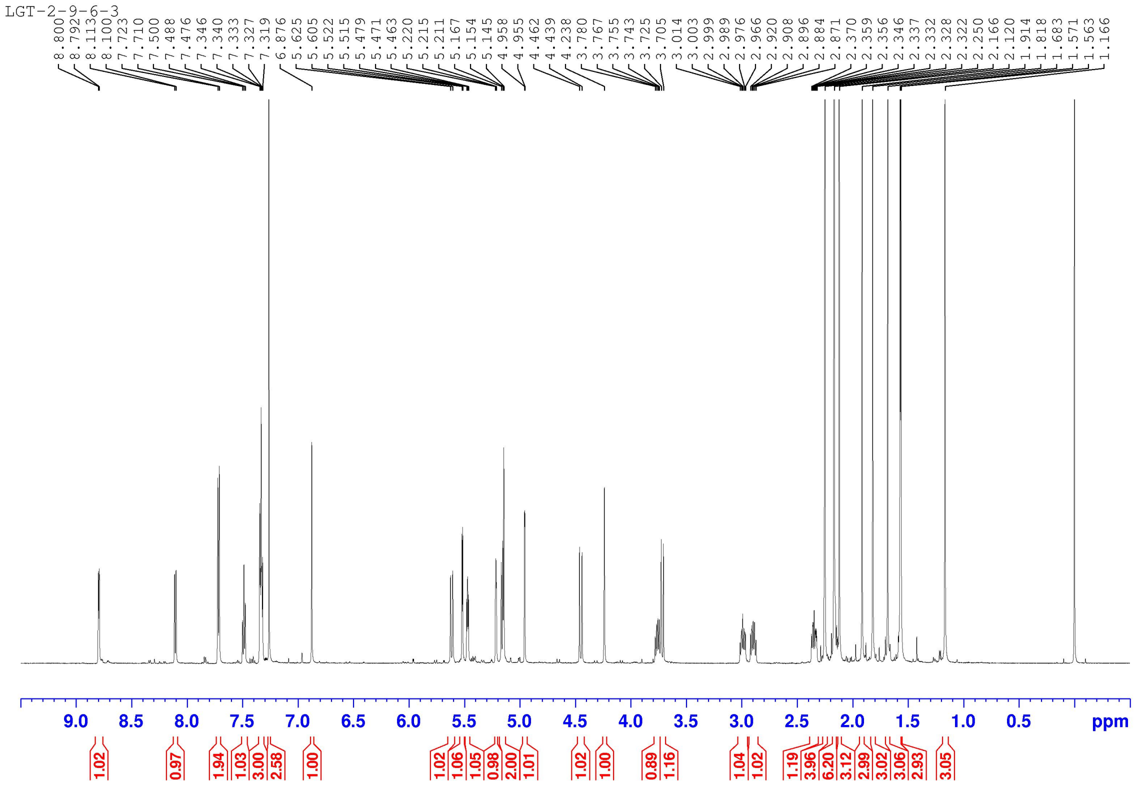

Figure S106. ^{1}H-NMR spectrum of compound **15** ($CDCl_3$, 600 MHz)

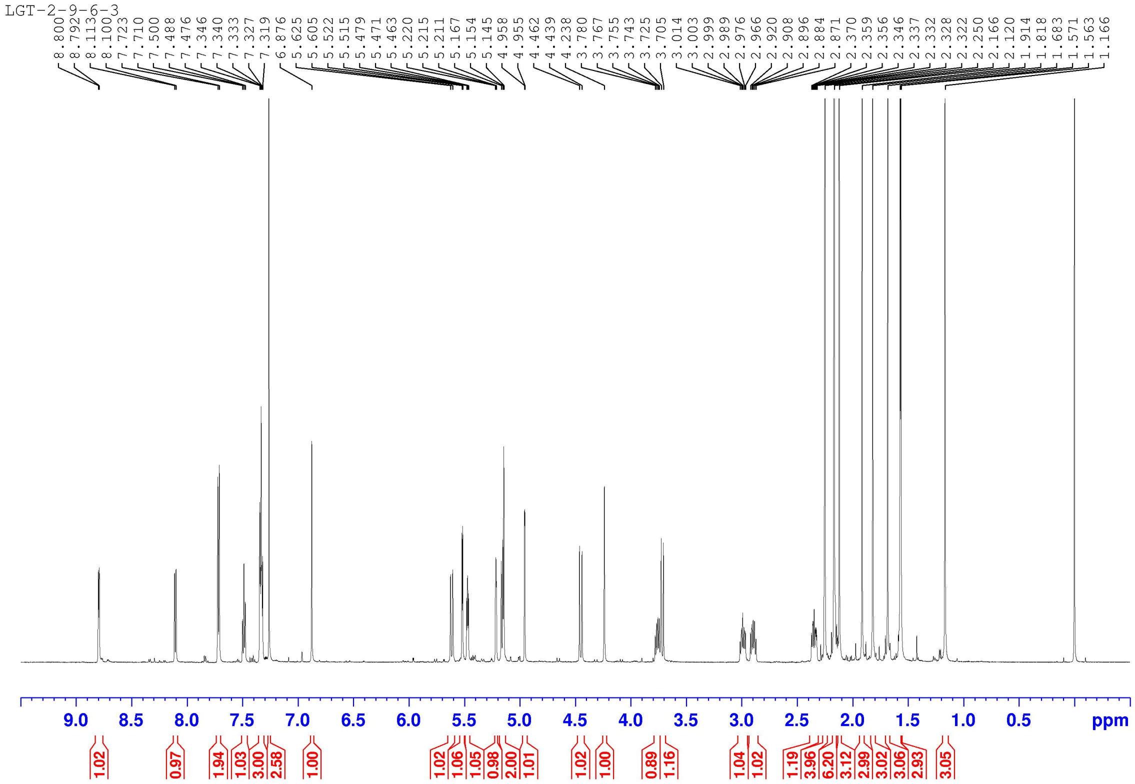

Figure S107. ^{13}C-NMR spectrum of compound **15** ($CDCl_3$, 150 MHz)

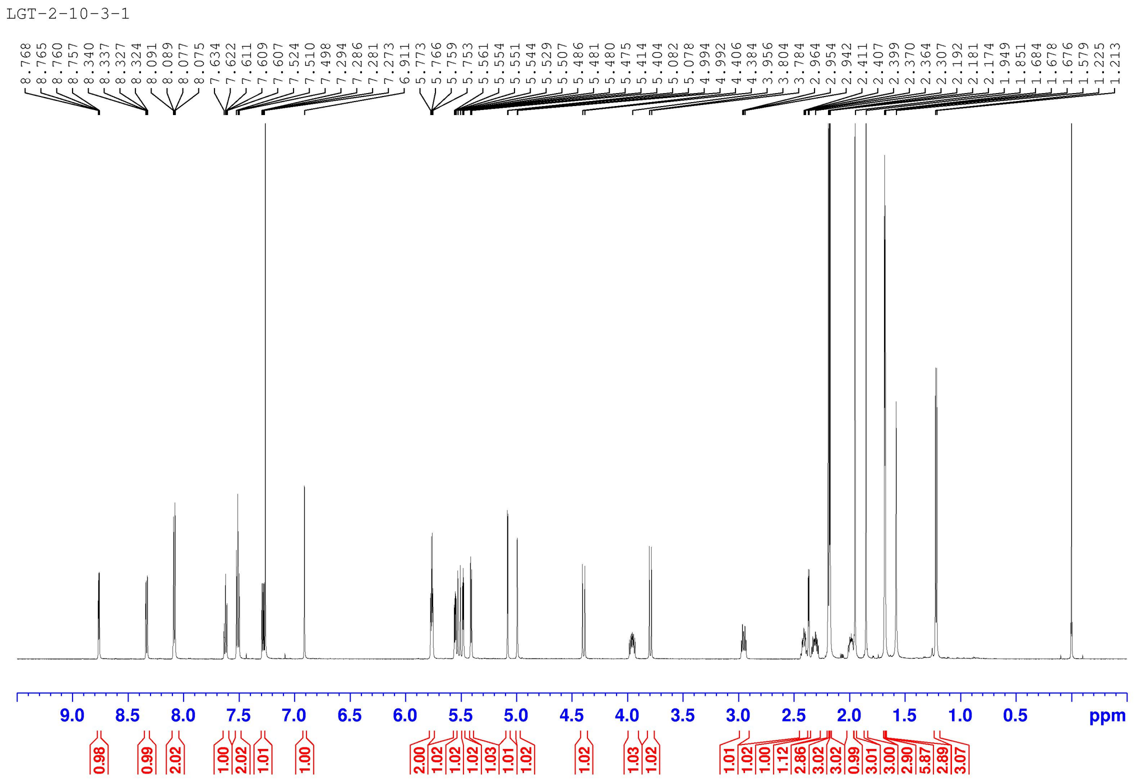

Figure S108. ^{1}H-NMR spectrum of compound **16** ($CDCl_3$, 600 MHz)

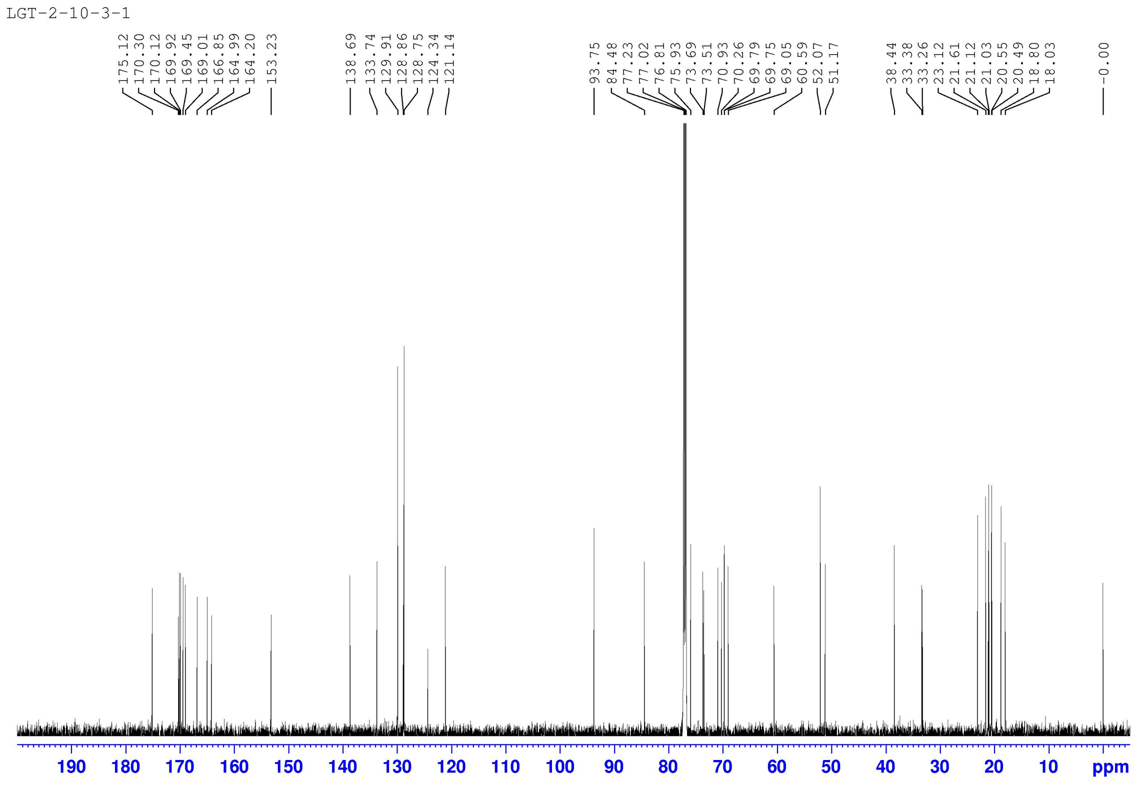

Figure S109. ^{13}C-NMR spectrum of compound **16** ($CDCl_3$, 150 MHz)

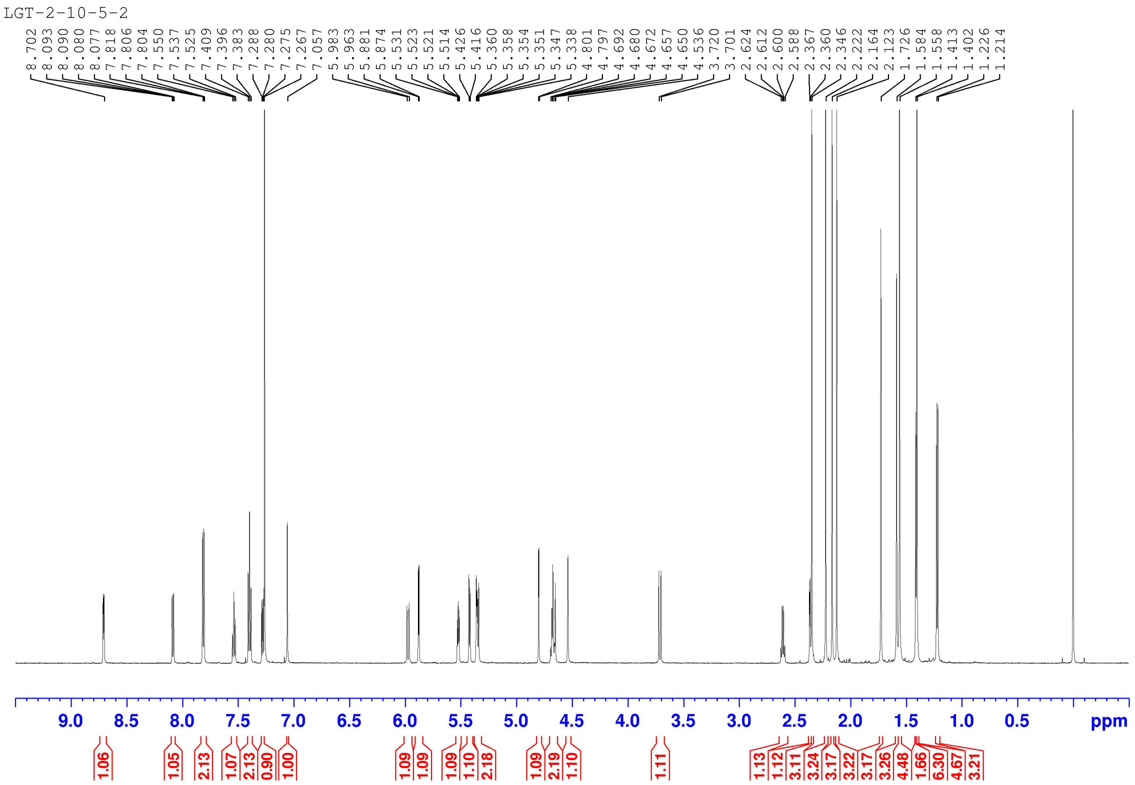

Figure S110. ^{1}H-NMR spectrum of compound **17** ($CDCl_3$, 600 MHz)

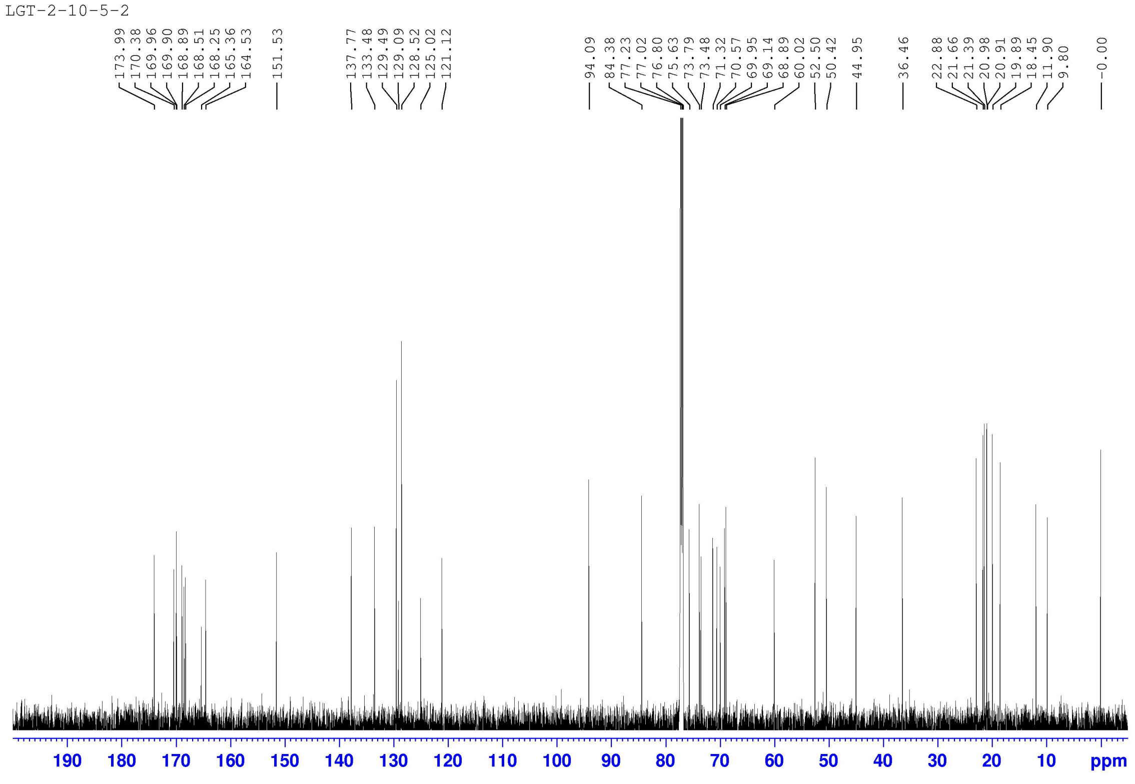

Figure S111. ^{13}C-NMR spectrum of compound **17** ($CDCl_3$, 150 MHz)

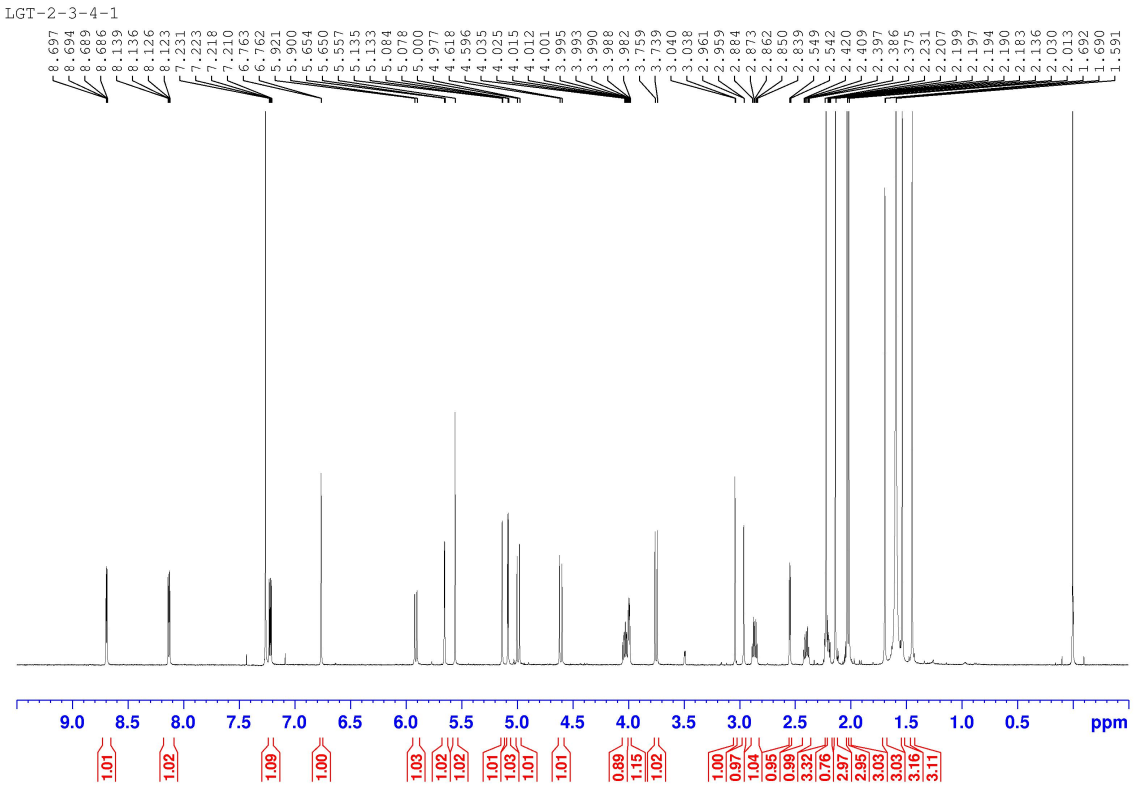

Figure S112. ^{1}H-NMR spectrum of compound **18** ($CDCl_3$, 600 MHz)

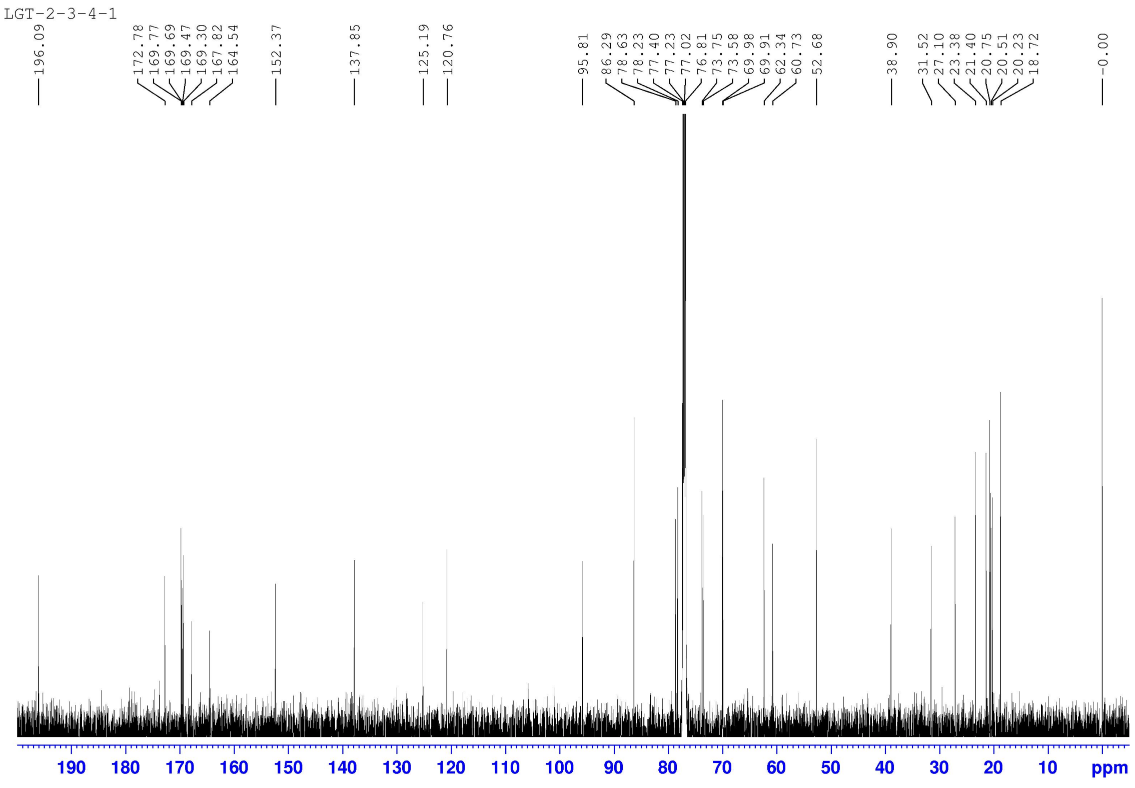

Figure S113. ^{13}C-NMR spectrum of compound **18** ($CDCl_3$, 150 MHz)

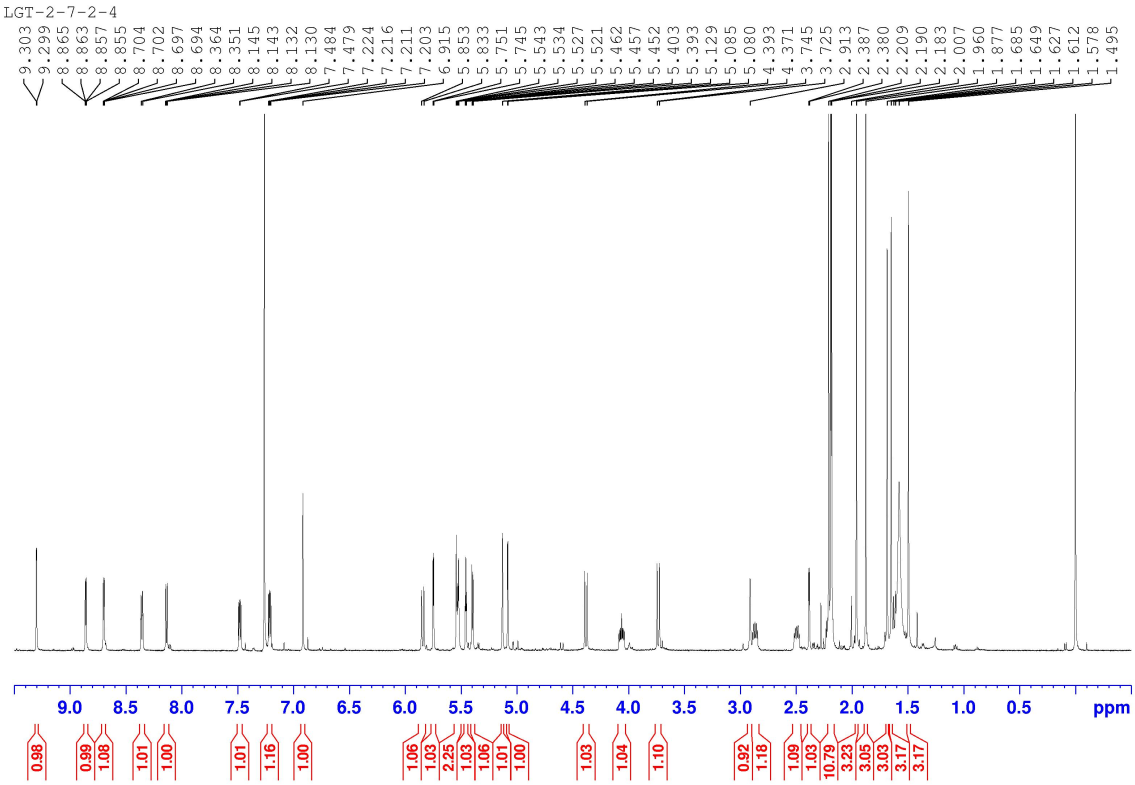

Figure S114. ^{1}H-NMR spectrum of compound **19** ($CDCl_3$, 600 MHz)

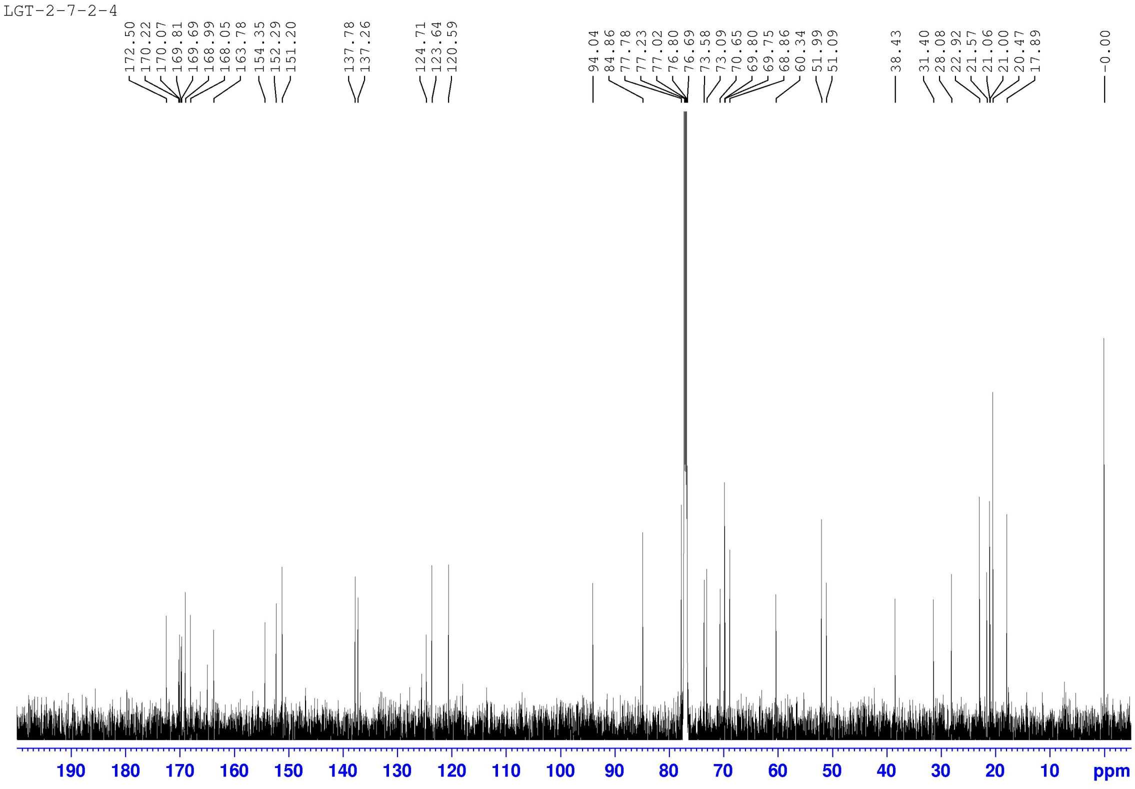

Figure S115. ^{13}C-NMR spectrum of compound **19** ($CDCl_3$, 150 MHz)

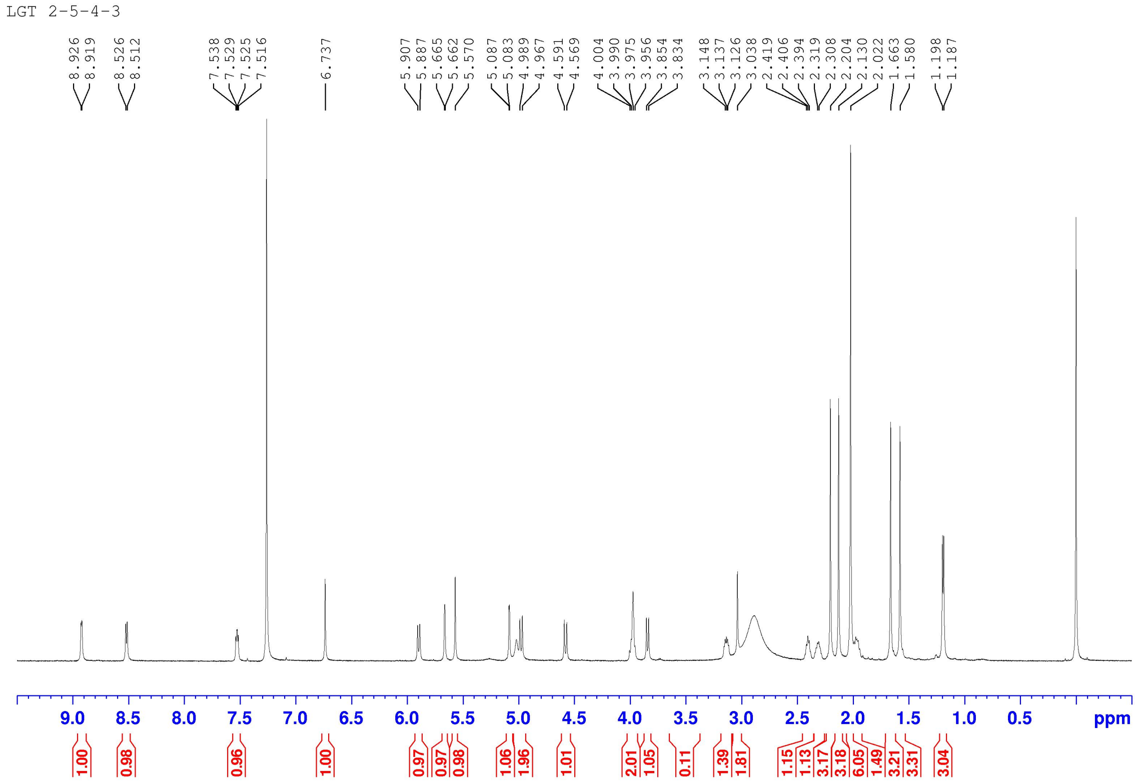

Figure S116. ^{1}H-NMR spectrum of compound **20** ($CDCl_3$, 600 MHz)

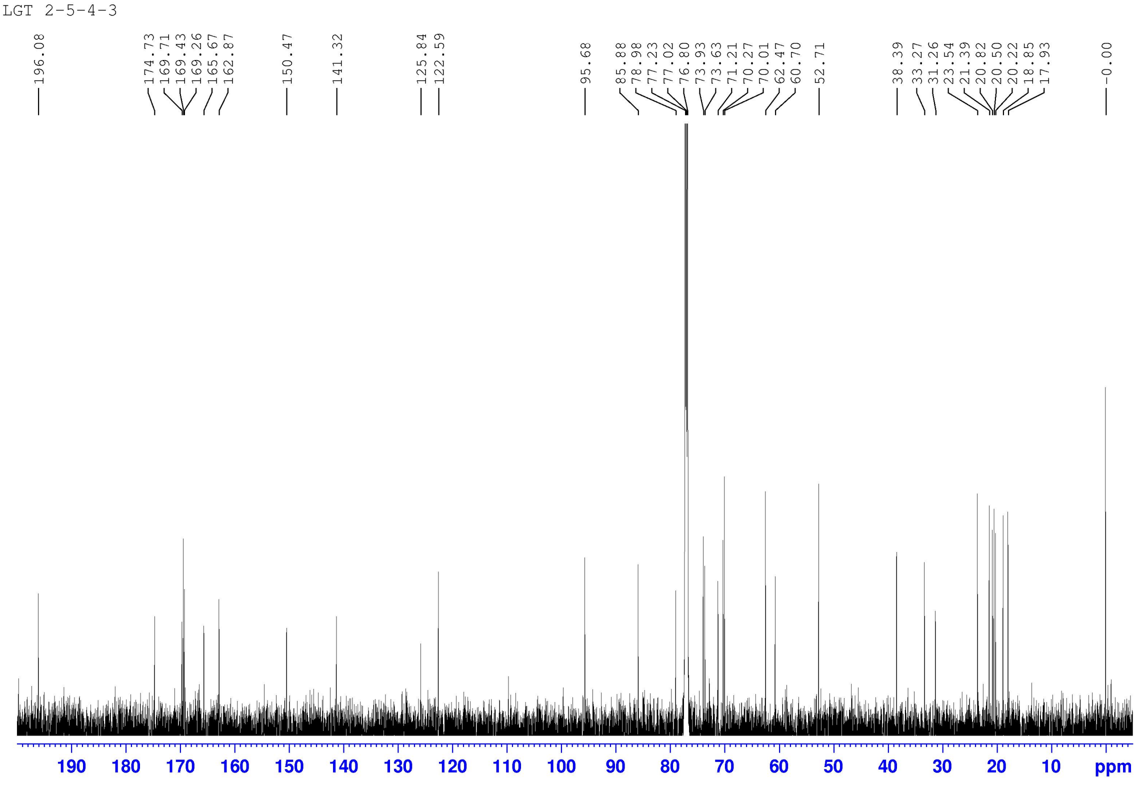

Figure S117. ^{13}C-NMR spectrum of compound **20** ($CDCl_3$, 150 MHz)

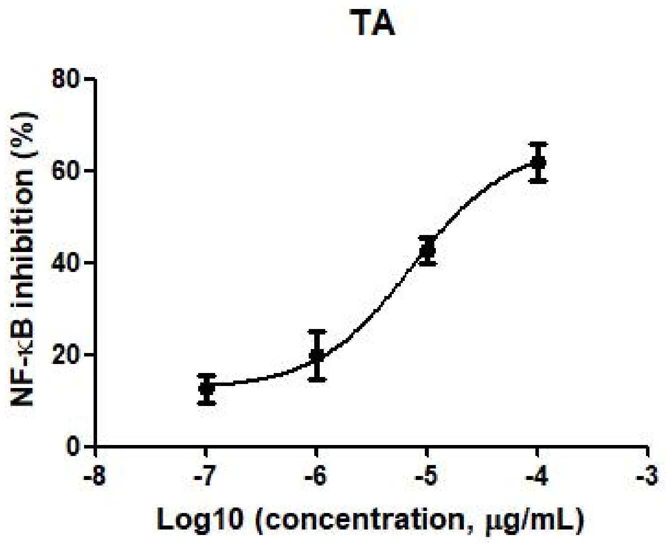

Figure S118. NF-κB inhibitory effect of Total alkaloids of *T. wilfordii* (TA)

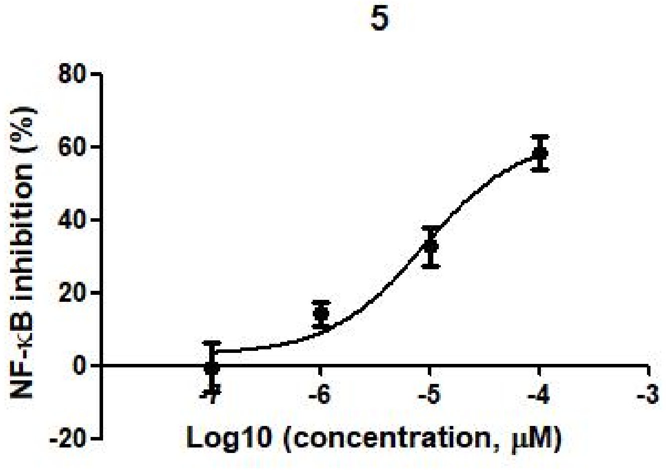

Figure S119. NF-κB inhibitory effect of compound **5**

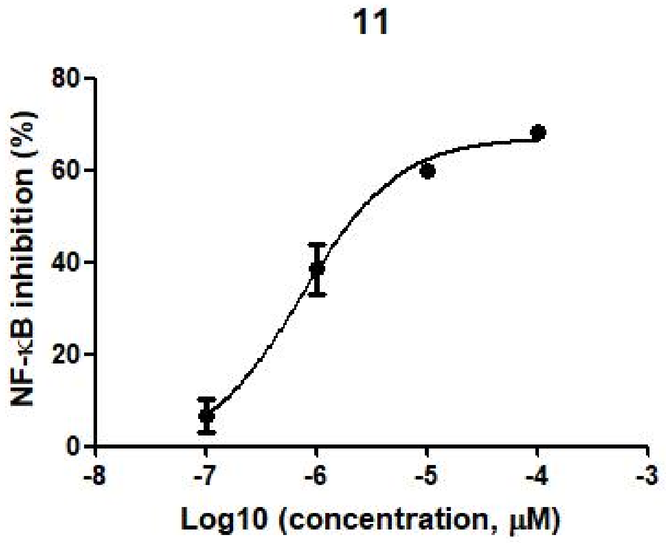

Figure S120. NF-κB inhibitory effect of compound **11**

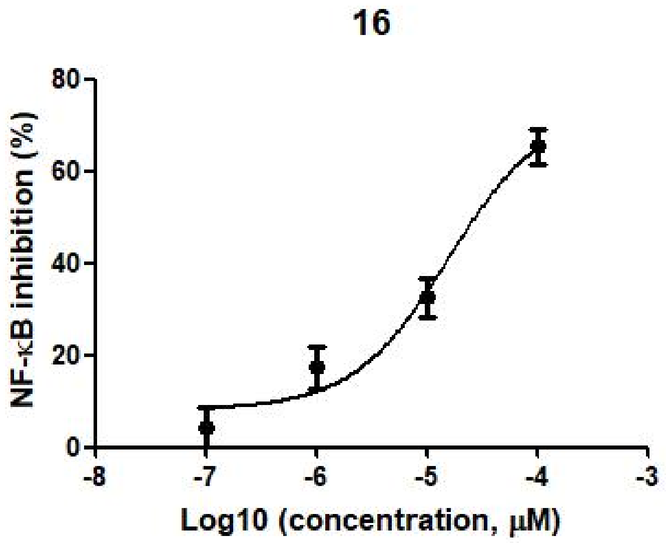

Figure S121. NF-κB inhibitory effect of compound **16**
